# Comparative cardiovascular outcomes in the era of novel anti-diabetic agents: a comprehensive network meta-analysis of 166,371 participants from 170 randomized controlled trials

**DOI:** 10.1186/s12933-018-0722-z

**Published:** 2018-06-05

**Authors:** Xiao-dong Zhuang, Xin He, Da-ya Yang, Yue Guo, Jian-gui He, Hai-peng Xiao, Xin-xue Liao

**Affiliations:** 1 Department of Cardiology, The First Affiliated Hospital of Sun Yat-sen University, Guangzhou, People’s Republic of China; 2 Key Laboratory on Assisted Circulation, Ministry of Health, No. 58 Zhongshan 2nd road, Guangzhou, 510080 People’s Republic of China; 3 Department of Endocrinology, The First Affiliated Hospital of Sun Yat-sen University, Guangzhou, People’s Republic of China

**Keywords:** Cardiovascular, Meta-analysis, Mortality, Diabetes, Agents

## Abstract

**Background:**

Cardiovascular (CV) safety of one anti-diabetic medication over another remains partially delineated. We sought to assess the comparative effect on CV outcomes among novel anti-diabetic agents.

**Methods:**

This study was registered with the International Prospective Register of Systematic Reviews (CRD 42016042063). MEDLINE, EMBASE, and Cochrane Library Central Register of Controlled Trials were searched between Jan 1, 1980, and June 30, 2016. Randomized controlled trials comparing anti-diabetic drugs with other comparators in adults with type 2 diabetes were included. We used network meta-analysis to obtain estimates for the outcomes of interests. In addition, post hoc correlation analysis of severe hypoglycemia and primary outcome as per ranking order was conducted. Outcomes were major adverse cardiovascular events (MACE) and all-cause mortality.

**Results:**

A total of 170 trials (166,371 participants) were included. By class and by individual, sulfonylureas (SU) ranked last. Therefore, with SU as reference, categorically sodium-glucose co-transporter 2 inhibitor (SGLT2i), insulin (INS), glucagon-like peptide-1 receptor agonist, and dipeptidyl peptidase 4 inhibitor were significantly superior in term of MACE; as were SGLT2i and INS in term of all-cause mortality. Moreover, ranking orders of MACE and all-cause mortality were both positively correlated with that of severe hypoglycemia risk (by individual: R^2^ = 0.3178, P = 0.018; by class: R^2^ = 0.2574, P = 0.038).

**Conclusions:**

Novel anti-diabetic agents possess favorable CV safety profile, despite small but robust differences between individuals. In addition, increase in CV risk was again shown to be partly attributable to a concomitant increase in the risk of severe hypoglycemia, for which SU performed the worst.

**Electronic supplementary material:**

The online version of this article (10.1186/s12933-018-0722-z) contains supplementary material, which is available to authorized users.

## Background

Cardiovascular (CV) safety of anti-diabetic medications had raised notable concern, so much so that, in December 2008, The US Food and Drug Administration (FDA) issued a guidance statement for industries requiring proof of CV safety for the recently approved novel anti-diabetic medications. In fact, the benefits and risks of using one anti-diabetic medication over another remain largely unknown. On the one hand, high-quality head-to-head comparison trials with important clinical endpoints, including long-term CV morbidity and mortality in particular, are still lacking. On the other, most systematic reviews and meta-analyses to date focused predominately on an individual agent or limited classes of agents [1–17]. In order to resolve this uncertainty, we performed a network meta-analysis to evaluate whether differences in CV outcomes exist between novel anti-diabetic medications, including dipeptidyl peptidase 4 inhibitors (DPP4i), glucagon-like peptide-1 receptor agonists (GLP1ra), and sodium-glucose co-transporter 2 inhibitors (SGLT2i), and the more traditional classes of drugs, including insulin (INS), metformin (MET), sulfonylureas (SU) and thiazolidinedione (TZD). In doing so, we aimed at providing evidence-based hierarchies of the comparative CV safety profiles among anti-diabetic agents.

## Methods

### Study design and protocol

We followed a pre-specified study protocol (Additional file 1: S1) and reported our results according to the Preferred Reporting Items of Systematic Reviews and Meta-Analyses (PRISMA) statement [18]. This study is registered with the International Prospective Register of Systematic Reviews (CRD 42016042063). Network meta-analysis integrates data from direct comparisons of treatments within trials and from indirect comparisons of interventions assessed against a common comparator in different trials, to compare all investigated treatments. The network meta-analysis was based on a frequentist model [19].

### Data sources and study selection

We searched MEDLINE, EMBASE, and the Cochrane Library Central Register of Controlled Trials between Jan 1, 1980, and June 30, 2016 (search strategy in Additional file 1: S2). In order to determine whether the study reported any event of interested outcomes, data on http://www.clinicaltrials.gov were also checked if registry number was provided.

Studies meeting the following criteria were included: randomized controlled trial; individuals with type 2 diabetes; comparison of anti-diabetic drugs with other positive comparator drugs or placebo (PLA); had at least one of incidence of outcomes mentioned in the next section, and reported number of patients and events in each treatment group; treatment durations of 24 weeks or longer. There is no limitation of baseline treatments as long as they are comparable in all of the study arms. Diabetic patients with concomitant diseases or CV risk factors were also included, but these studies would be excluded in sensitivity analysis. Studies that compared the different dosages or forms of the same drug were excluded. Studies were excluded if they were crossover trials, quasi experiments, non-randomized trials, or enrolled patients with type 1 diabetes or patients without diabetes but only INS resistance.

### Novel anti-diabetes agents and dosages

Novel anti-diabetic agents refer to the following three classes: DPP4i, GLP1ra and SGLT2i. We only included drugs that have been approved by either US FDA or European Medicines Agency. Comparators can be PLA, MET, SU, TZD, INS, and another novel anti-diabetic drug mentioned above. The treatment arm for these novel drugs that used recommended dosages was analyzed (Additional file 1: S3).

### Data extraction and quality assessment

Outcomes of interest were major adverse cardiovascular events (MACE), which consisted of CV death, non-fatal myocardial infarction (MI), non-fatal stroke, and unstable angina or hospitalization for unstable angina, and all-cause mortality. We included severe hypoglycemia as an outcome during the data extraction phase for post hoc analysis. Severe hypoglycemia was defined as hypoglycemia episode requiring the assistance of another person or medical assistance, regardless of documentation of blood glucose.

Two reviewers independently scanned the search results by reading the titles and abstracts. Data extracted included outcomes of interest, study characteristics (registry number, name the first author, whether it was international study, number of study centers, treatment duration), participant characteristics (mean age, concomitant high risk factor, proportion of male patients), intervention details (type of drug, its dosage in each arm and baseline drug used across arms).

The methodological quality of included RCTs was assessed using the tool described in the Cochrane collaboration handbook [20]. Briefly, this tool includes seven components, which are random sequence generation, allocation concealment, blinding of participants and personnel, blinding of outcome assessment, incomplete outcome data, selective reporting and other sources of bias. Each of these components of every included study received a rating of “low risk”, “unclear”, or “high risk”.

### Statistical analysis

Stata package (version 14) was applied for statistical analyses, using the network and mvmeta command and Stata routines described elsewhere [21]. For indirect and mixed comparisons, we used network meta-analysis to obtain estimates for the outcomes, and presented these estimates as odd ratios (OR) with 95% confidence intervals (CI). We then estimated the relative ranking probability of each treatment and obtained the treatment hierarchy of competing interventions using rankograms, surface under the cumulative ranking (SUCRA) curve, and mean ranks. Large SUCRA scores might indicate a more effective or safer intervention [22]. We showed the results using SU as reference in interval plot because it consistently ranked last. In addition, we chose not to present MET in the ranking as it was used as background treatment in most of the trials.

To check for the presence of inconsistency, we used the loop-specific approach that assesses the difference between direct and indirect estimates for a specific comparison in the loop (inconsistency factor) [23]. We assumed a common heterogeneity estimate within each loop. We used the previously described node-splitting method, which separates evidence for a particular comparison into direct and indirect, excluding one direct comparison at a time and estimating the indirect treatment effect for the excluded comparison [24]. A comparison adjusted funnel plot of treatment estimates for drug on CV outcomes was used to assess for evidence of small-study effects.

To investigate the generalizability of the findings, we assessed the effect of characteristics of trials and participants on the outcomes in sensitivity analyses by excluding studies with the following design characteristics: patients with high CV risk; patients with renal impairment; and sample size less than 100 in one arm.

Finally, to explore the potential impact of severe hypoglycemia on the association between anti-diabetic drugs and CV outcomes, additional correlation analysis of severe hypoglycemia and outcome of interest according to the ranking order was conducted.

## Results

The PRISMA flowchart showing electronic searching processes is shown in Additional file 1: S4. There were 170 randomized controlled trials including 166,371 adults eligible for the systematic review, reporting 8702 cases of MACE (33.5 per 1000 patient-year) and 4914 cases of all-cause mortality (18.3 per 1000 patient-year). Seven drug classes were compared with PLA or each other: DPP4i, GLP1ra, SGLT2i, MET, SU, TZD and INS. For individual comparison, 18 treatment groups were analyzed: alogliptin, linagliptin, saxagliptin, sitagliptin, vildagliptin, albiglutide, dulaglutide, exenatide, liraglutide, lixisenatide, canagliflozin, dapagliflozin, empagliflozin, MET, SU, TZD, INS and PLA. Most trials (159 [93.5%] of 170) were two-armed studies. Studies characteristic and outcomes were shown in Additional file 1: S5, S6. Treatment duration ranged from 24 to 208 weeks. Male patients ranged from 40.9 to 75%. Mean age of patients was ranged from 44.0 to 74.9 years. Ten studies enrolled subjects with high CV risk (65,650 patients), and 9 studies were exclusively of patients with renal impairment (1349 patients).

### Study quality assessment

The overall quality of studies was rated as good, even though some studies did not record details about randomization and allocation concealment and there were only few randomized trials at low risk of bias in every question-based entry (Additional file 1: S7). Moreover, no major tendency was noted for smaller studies to overestimate or underestimate active treatment effects on outcomes in the comparison-adjusted funnel plot for the network (Additional file 1: S8).

### Mace and all-cause mortality

**Fig. 1:**
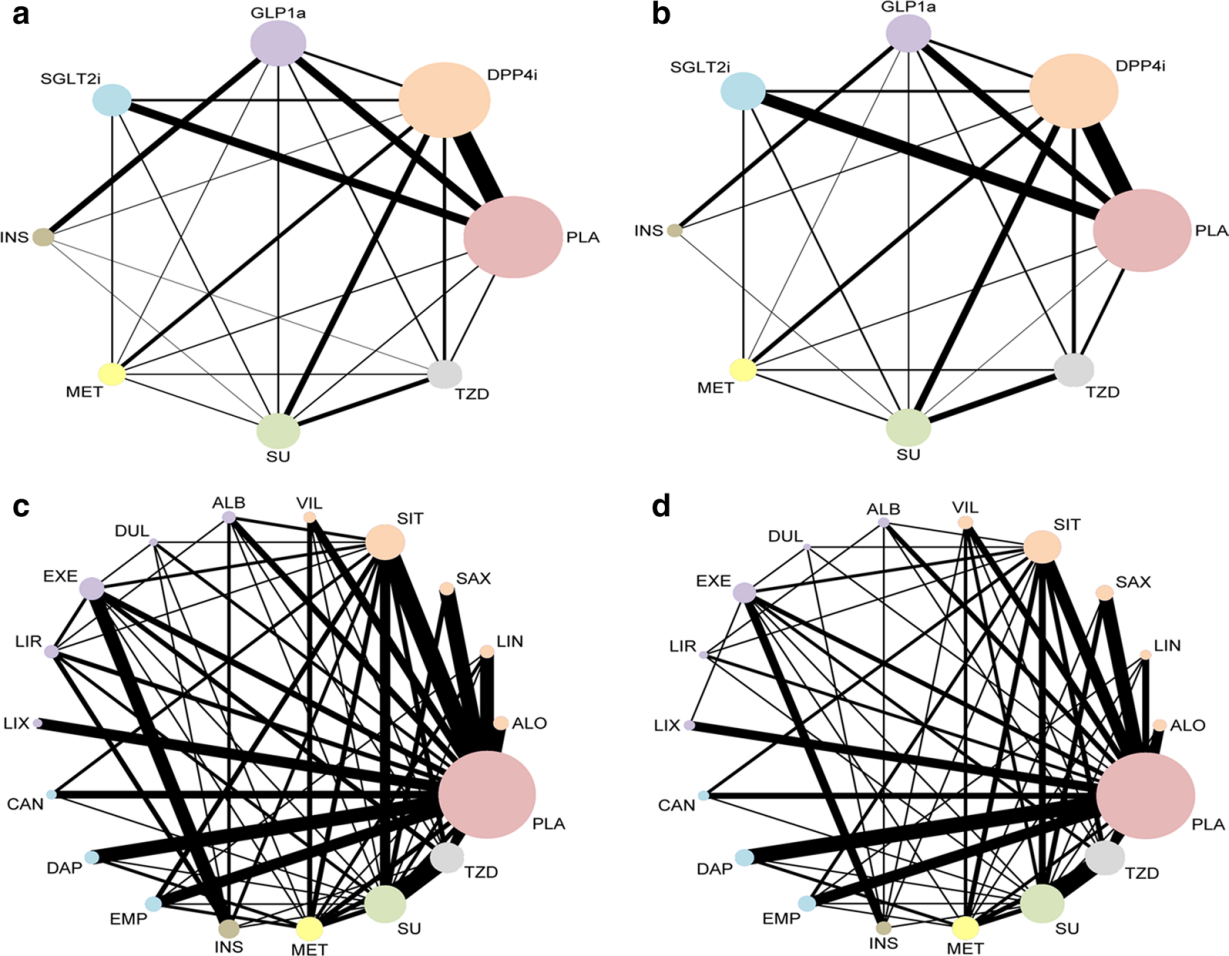
Network plot of treatment comparisons for major adverse cardiovascular events (MACE) and all-cause mortality. **a** Categorized drugs comparisons for MACE; **b** Categorized drugs comparisons for all-cause mortality; **c** Individual drugs comparisons for MACE; **d** Individual drugs comparisons for all-cause mortality. The size of the nodes represents the number of trials that study the treatments. Direct comparison of treatments is linked with a line, the thickness of which represents the number of trials that assess the comparison. *SGLT2i* sodium-glucose co-transporter 2 inhibitor(s), *GLP1ra* glucagon-like peptide-1 receptor agonist(s), *DPP4i* dipeptidyl peptidase 4 inhibitor(s), *TZD* thiazolidinedione, *MET* metformin, *SU* sulfonylurea, *INS* insulin, *PLA* placebo, *VIL* vildagliptin, *EMP* empagliflozin, *LIX* lixisenatide, *ALO* alogliptin, *EXE* exenatide, *LIR* liraglutide, *CAN* canagliflozin, *DAP* dapagliflozin, *DUL* dulaglutide, *SIT* sitagliptin, *LIN* linagliptin, *ALB* albiglutide, *SAX* saxagliptin

Networks of eligible comparisons (among individual agents or classes of agents) for the CV outcomes were presented in Fig. 1, showing predominantly pairwise comparisons of drugs with DDP4i or PLA in classed groups. Except for INS and SGLT2i, SGLT2i and GLP1ra, as well as INS and MET, direct evidence for MACE was available for all the possible pairwise treatment comparisons. However, such availability was lacking between individual drugs.

**Fig. 2:**
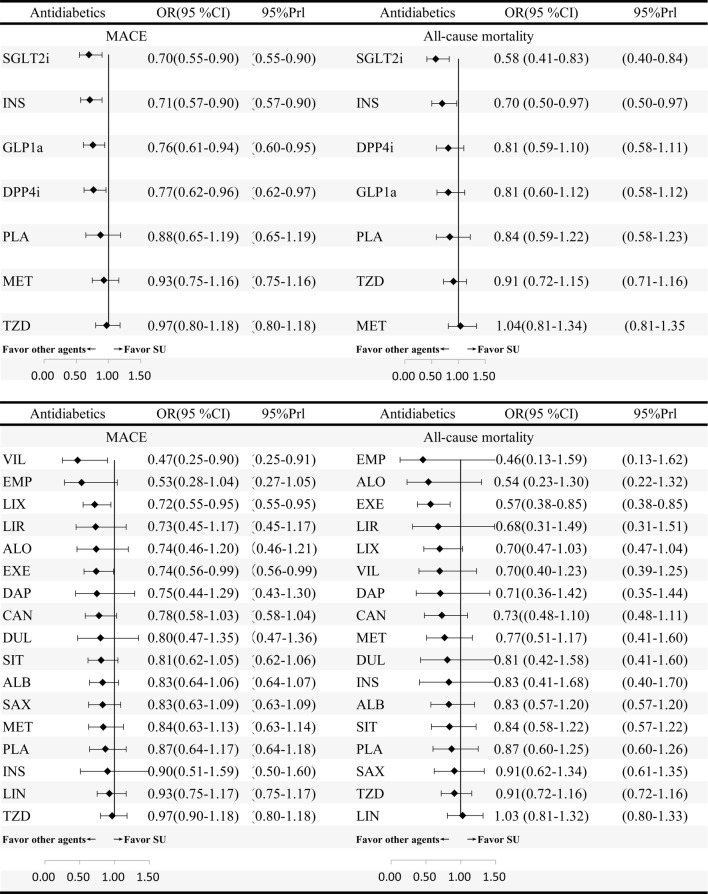
Forest plot for MACE and all-cause mortality of anti-diabetic agents compared with sulfonylurea (individual and categorized agents). Treatments are ranked by surface under the cumulative ranking (SUCRA) values. *OR* odds ratio, *CrI* credibility interval

**Fig. 3:**
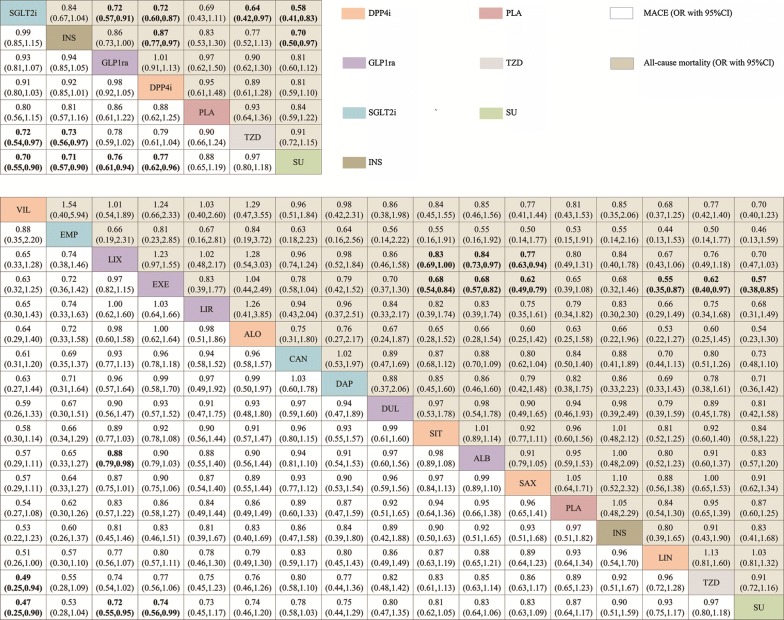
Mixed comparison results of anti-diabetic agents for MACE and all-cause mortality, both for individual (above table) and for categorized agents (below table). Agents are reported in order of MACE ranking. Treatment at the top left corner ranks first, while the one at the bottom right corner ranks last. OR lower than 1 favor the column-defining treatment. Anti-diabetic agents in one class are painted with the same color. Significant results are in bold

In the network meta-analyses, MACE were reported in 152 studies (158,786 patients with 8702 MACE). Comparative effects of all drugs were ranked with SUCRA probabilities (Additional file 1: S9). Mixed comparisons were in the interval plot with SU as reference (Fig. 2) and the comparisons table (Fig. 3). In term of MACE, mixed comparisons by drug class showed that SGLT2i (OR 0.70, 95% CI 0.55–0.90), INS (0.71, 95% CI 0.57–0.90), GLP1ra (OR 0.76, 95% CI 0.61–0.94), and DPP4i (OR 0.77, 95% CI 0.62–0.9) were significantly better than SU, and SGLT2i (OR 0.72, 95% CI 0.54–0.97) and INS (OR 0.73, 95% CI 0.56–0.97) were superior to TZD. Mixed comparisons by individual drug showed that vildagliptin (OR 0.47, 95% CI 0.25–0.90), lixisenatide (OR 0.72, 95%CI 0.55–0.95) and exenatide (OR 0.74, 95% CI 0.56–0.99) were significantly better than SU. Moreover, vildagliptin (OR 0.49, 95% CI 0.25–0.94) was significantly superior to TZD and lixisenatide (OR 0.49, 95% CI 0.25–0.94) was significantly superior to albiglutide. By applying the design-by-treatment inconsistency model, we detected inconsistency in only one loop of comparisons: SU-TZD (*P *= 0.042) (Additional file 1: S10).

All-cause mortality was reported in 139 studies (159,722 patients with 4914 death). Mixed comparisons were in the interval plot with SU as reference (Fig. 2) and the comparisons table (Fig. 3). In term of all-cause mortality, mixed comparisons by drug class showed that SGLT2i was significantly better than GLP1ra (OR 0.72, 95% CI 0.57–0.91), DPP4i (OR 0.72, 95% CI 0.60–0.87), TZD (OR 0.64, 95% CI 0.42–0.97) and SU (OR 0.58, 95% CI 0.41–0.83). Moreover, INS was significantly better than DPP4i (OR 0.87, 95% CI 0.77–0.97) and SU (OR 0.70, 95% CI 0.50–0.97). Mixed comparisons by individual drug showed that exenatide was significantly better than albiglutide (OR 0.68, 95% CI 0.57–0.82), saxagliptin (OR 0.62, 95% CI 0.49–0.79), linagliptin (OR 0.55, 95% CI 0.35–0.87), TZD (OR 0.62, 95% CI 0.40–0.97) and SU (OR 0.57, 95% CI 0.38–0.85), and lixisenatide was significantly superior to albiglutide (OR 0.84, 95% CI 0.73–0.97) and saxagliptin (OR 0.77, 95% CI 0.63–0.94). We did not observe any inconsistencies between evidence derived from direct to indirect comparisons for all-cause mortality using the design-by-treatment inconsistency model (Additional file 1: S10).

### Sensitivity analyses and post hoc correlation analysis

Results for MACE were generally robust in sensitivity analyses by excluding studies with the following design characteristics: patients with high CV risk; patients with renal impairment; and sample number less than 100 in one arm. After the sensitivity analyses, changes in ORs and rankings, either categorical drugs or individual drugs, did not alter the primary results appreciably (Additional file 1: S11).

**Fig. 4:**
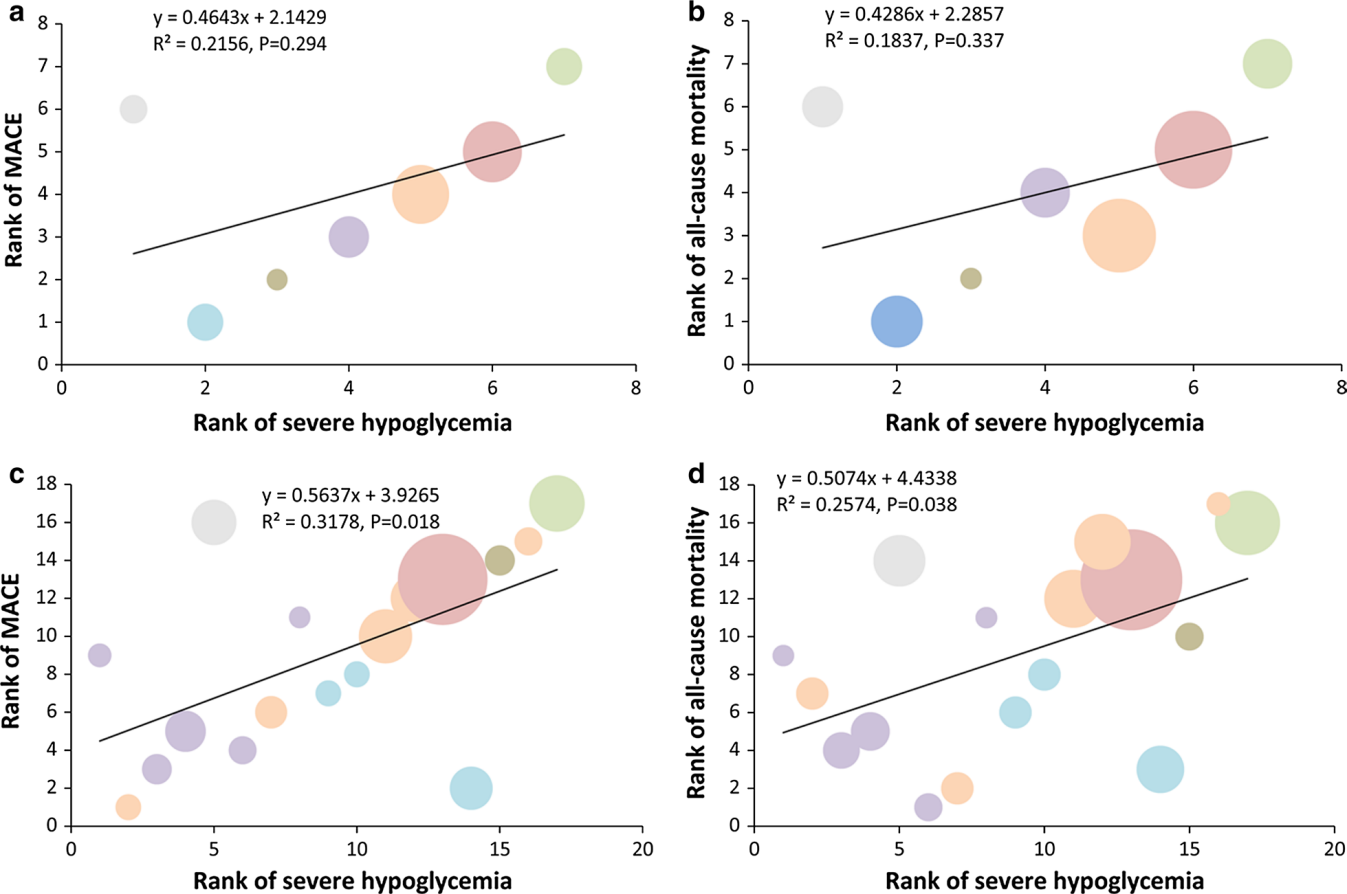
Correlation analyses showing correlation between the ranking order of MACE and all-cause mortality risk and the ranking order of severe hypoglycemia risk. Color of circle represents different drugs shown above. Area of a circle reflects sample size. **a**, **b**, **c**, **d** The panels indicates the correlation relationship between severe hypoglycemia and outcome of interest according to the ranking order

As is shown in the post hoc correlation analysis (Fig. 4), for individual drugs, the ranking order of MACE or all-cause mortality was positively correlated with the ranking order of severe hypoglycemia (R^2^ = 0.3178, *P *= 0.018; R^2^ = 0.2574, *P *= 0.038, respectively), whereas for drug classes, a similar trend was observed with TZD as an outlier.

## Discussion

To the best of our knowledge, our network meta-analysis represents the most comprehensive synthesis of data currently available with regard to CV outcomes in pharmacologically managed patients with type 2 diabetes. Our findings can be summarized as follows: first, among anti-diabetic agents included in the network, SGLT2i in class comparisons, and vildagliptin in individual comparisons, respectively ranked first in terms of MACE. Furthermore, when compared with other individual or classes of drugs, SU are associated with the highest risks of MACE and all-cause mortality. Finally, the ranking of CV risk was linearly correlated with the ranking of severe hypoglycemia risk by individual comparisons, with SU displaying the highest risks in both endpoints.

Our study found that the newer agents in general showed favorable CV safety, yet there are discrepancies between individual and class comparisons. In a recently published meta-analysis, the DPP4i vildagliptin was found to significantly reduce the risk of MI and stroke, while other agents in the same class seemed to perform less well in terms of CV outcomes [3]. In two recent studies, the use of DPP4i was found to be associated with improved long-term survival in diabetic patients surviving a myocardial infarction [15] whereas its increase in overall risks of heart failure or exhibit within-class differences remains unresolved [7]. These results were reiterated in our study, in that despite vildagliptin displayed the best CV safety profile individually, and in class comparisons the ranking of DPP4i actually dropped to the fifth place in order. Other discrepancies are also identifiable and can be resolved similarly. In light of the mixed results, therefore, a case can perhaps be made against a “class effect” in the era of novel anti-diabetic medications, namely, the fact that a well-documented better (or worse) CV safety profile of one individual agent does not necessarily justify extrapolation of such a benefit (or harm) to other agents in the same class.

These results have practical implications. Several appeals have recently been made for an appraisal of the current paradigm to evaluate CV risks of novel anti-diabetic medications via large-scale, long-term CV safety trials [25, 26]. The authors argued for alternative approaches that are more cost-effective, more externally valid, and better informed. Such a “targeted” strategy, nonetheless, ought not to have indiscreetly relied too much, if at all, on a “known class effect”, but rather should be individualized and outcome-specific, if (and only if) the signals of harm were detected in pre-approval package or post-approval monitoring of these newer agents.

On the other hand, albeit primary results being “mixed”, our analysis had once again confirmed that SU were associated with the highest risks of MACE and all-cause mortality. In fact, SU steadily brought up the rear in both individual and class ranking, even after sensitivity analysis. When plotting the ORs of MACE or all-cause mortality for all other comparator drugs against SU, individually, point estimates concordantly lie to the left of the “line of no effect”; collectively, by order of effect size, SGLT2i, INS, GLP1ra and DPP4i were significantly better than SU, indicating that SU in actuality possess the worst CV safety profile among these medications.

SU are currently the most widely used medications for type 2 diabetes second only to MET. However, the undesirable effect of weight gain [27], the greatest risk of iatrogenic hypoglycemia [28–31], a potential increase in CV morbidity and mortality [32–36], and adding to that, the advent of novel anti-diabetic medications with arguably equal glucose-lowering effectiveness [37], all render SU as less favorable [38]. In a recent commentary, the role of SU in the era of novel anti-diabetic medications was thoughtfully challenged [39]. And according to the latest management guideline jointly issued by the American Association of Clinical Endocrinologists and the American College of Endocrinologists, in combinational regimens, the strength of recommendation for SU to be added on top of MET is the weakest [40].

One of the major controversies about SU is their CV safety. For example, in the UK Prospective Diabetes Study (UKPDS), in patients treated with SU, there was a trend of a 16% decrease in MI at the end of the study, but at 10-year follow up there was a significant 15% decrease in events in the same arm [41]. In a recent network meta-analysis, the authors reported no significant differences in the associations between nine classes of anti-diabetic medications and the risk of CV or all-cause mortality [37]. Unfortunately, however, due to low event rates in general as well as the statistical power being diluted by multiple layers of analyses in particular, conclusions about true effects of the studied drugs on CV or all-cause mortality were far from precise. The contribution of our study, then, is having further clarified the current confusion by offering an evidence-based hierarchy of CV safety profile among all major anti-diabetic medications, which revealed the steady truth that SU are associated with the highest risks of MACE or all-cause mortality when compared with other individual or classes of agents.

In our post hoc correlation analysis, the ranking of MACE and mortality risk were both linearly correlated with the ranking of severe hypoglycemia risk by individual comparisons, with SU at the top of risks, corroborating the already well-founded connection, if not causality, between the elevated risk of iatrogenic hypoglycemia with the use of SU and the inferior CV safety of this class of traditional anti-diabetic agents, although the effect of “drug–drug interaction” cannot be completely ruled out, because many of these trials had multi-drug regimens. In addition, it is of note that TZD are shown to be an outlier in both correlations, with higher ranking of CV risk but lower ranking of hypoglycemia. Such a result could be explained by the previous observation that the use of TZD (especially rosiglitazone) is associated with potential increase of CV risks independent of hypoglycemia [42].

Our study is limited in several ways. First, although comprehensive systematic search strategies were employed, our analyses were limited by the modest amount of data in the included studies. To begin with, due to heterogeneity of study designs and reporting styles in the included studies, comparison of A1c levels as well as the degree of CV risk at baseline across different trials were unavailable. In addition, only a few studies reported outcomes such as acute coronary syndrome and CV death, and most had few or zero events. Some subgroup analyses had small numbers of participants, likely resulting in poor precision of estimates. Second, many of the international multicenter trials were conducted primarily in higher-income countries, which would possibly interfere with the external validity of these results for lower-income settings. Third, follow-up duration in most studies was relatively too short to draw any definitive conclusion for long-term CV outcome. Fourth, as first-line data from these trials being unaccessible to us, the present study was unable to account for other possible factors correlated with meaningful CV outcomes. Results from continuing trials would provide useful insight in answering these questions.

## Conclusions

Our network meta-analyses showed that all three classes of novel anti-diabetic medications, i.e. DPP4i, GLP1ra, and SGLT2i, possess favorable CV safety profile in general, notwithstanding that there are small but robust differences among individual drugs. These results refute a simplistic rationale of generalizing the CV benefit of one single agent to the others in the same class. In addition, we also observed that SU were associated with the highest risk of MACE and all-cause mortality, which could potentially explained by its concomitant increase in the risk of severe hypoglycemia. Such correlation should probably call for a reassessment of the role of SU as first-line additive to MET in the pharmacological management of type-2 diabetes. These findings should be considered in policy-making and the development of clinical practice guidelines.

## Additional file

**Additional file 1: S1.** Protocol. **S2.** Search strategy. **S3.** Novel drugs approved by FDA or European Medicines Agency. **S4.** Flow chart of the study selection process. **S5.** Studies characteristic of the included studies. **S6.** Outcomes of interest in each study. **S7.** Quality assessment of the included studies. **S8.** Comparison-adjusted funnel plot for the network. **S9.** Ranking ordered according to surface under the cumulative ranking values of outcomes. **S10.** Consistency analysis of direct verse indirect comparisons for outcomes. **S11.** Summaries of sensitivity analysis.

## Abbreviations

CV: cardiovascular
FDA: The US Food and Drug Administration
DPP4i: dipeptidyl peptidase 4 inhibitor
GLP1ra: glucagon-like peptide-1 receptor agonist
SGLT2i: sodium-glucose co-transporter 2 inhibitor
INS: insulin
MET: metformin
SU: sulfonylurea
TZD: thiazolidinedione
PLA: placebo
MACE: major adverse cardiovascular event
MI: myocardial infarction
OR: odds ratio
CI: confidence interval
SUCRA: surface under the cumulative ranking
