# Supplementary material for: Comparative cardiovascular outcomes in the era of novel anti-diabetic agents: a comprehensive network meta-analysis of 166,371 participants from 170 randomized controlled trials
Source: Cardiovasc Diabetol. 2018 Jun 5;17:79. doi: 10.1186/s12933-018-0722-z (PMC5989345; doi:10.1186/s12933-018-0722-z)
Supplement: Supplementary file 1 — Additional file 1: S1. Protocol. S2. Search strategy. S3. Novel drugs approved by FDA or European Medicines Agency. S4. Flow chart of the study selection process. S5. Studies characteristic of the included studies. S6. Outcomes of interest in each study. S7. Quality assessment of the included studies. S8. Comparison-adjusted funnel plot for the network. S9. Ranking ordered according to surface under the cumulative ranking values of outcomes. S10. Consistency analysis of direct verse indirect comparisons for outcomes. S11. Summaries of sensitivity analysis. [file 12933_2018_722_MOESM1_ESM.docx]

Contents

[S1. Protocol 2](#_Toc494412281)

[S2. Search strategy 9](#_Toc494412282)

[S3. Novel drugs approved by FDA or European Medicines Agency 12](#_Toc494412283)

[S4. Flow chart of the study selection process 14](#_Toc494412284)

[S5. Studies characteristic of the included studies 15](#_Toc494412285)

[S6. Outcomes of interest in each study 23](#_Toc494412286)

[S7. Quality assessment of the included studies 37](#_Toc494412287)

[S8. Comparison-adjusted funnel plot for the network 44](#_Toc494412288)

[S9. Ranking ordered according to surface under the cumulative ranking values of outcomes 45](#_Toc494412289)

[S10. Consistency analysis of direct verse indirect comparisons for outcomes 46](#_Toc494412290)

[S11. Summaries of sensitivity analysis 51](#_Toc494412291)

# Additional file 1: S1. Protocol

**BACKGROUND**

Diabetes mellitus (DM) is currently affecting over 380 million people globally, with an 85-95% majority of cases being type-2 DM^1^. It was estimated to account for approximately 1.5 million deaths annually^2^. DM is medically managed by therapeutic lifestyle modification and the judicial use of anti-diabetic medications, which include insulin, metformin, sulfonylureas (SUs), thiazolidinediones (TZDs), alpha-glucosidase inhibitors, glitinides, dipeptidyl peptidase 4 (DPP-4) inhibitors, glucagon-like peptide-1 (GLP-1) receptor agonists, and sodium-glucose co-transporter 2 (SGLT-2) inhibitors. Cardiovascular (CV) safety of anti-diabetic medications had raised notable concern among clinicians and researchers, so much so that, in December 2008, the US Food and Drug Administration issued a guidance statement for industries requiring proof of CV safety for the recently approved novel anti-diabetic medications. In fact, the benefits and risks of using one anti-diabetic medication over another remain largely unknown. On the one hand, high-quality head-to-head comparison trials with important clinical endpoints, including long-term CV morbidity and mortality in particular, are still lacking. On the other, most systematic reviews and meta-analyses to date focused predominately on an individual agent or class of agents^3-7^. In order to resolve this uncertainty, we performed a network meta-analysis to evaluate whether differences in terms of CV outcomes exist between novel anti-diabetic medications, including the DPP-4 inhibitors, the GLP-1 receptor agonists, and the SGLT-2 inhibitors, and the more traditional classes of agents, such as insulin, metformin, SUs and TZDs. By analyzing the total body of evidence, we aimed at providing evidence-based hierarchies of the comparative CV safety profiles among anti-diabetic agents.

Specifically, the objectives of this study can be stated as follows:

1) To compare, both individually and categorically, the cardiovascular outcomes, including major adverse cardiovascular events, all-cause mortality, and new-onset heart failure among anti-diabetic drugs, in patients with type-2 diabetes mellitus

2) To generate clinically meaningful hierarchies of anti-diabetic medications according to their relative cardiovascular risk profiles

**METHODS**

**Inclusion criteria**

Types of participants

Patients had to aged 18 or older of both sexes and be diagnosed with type 2 diabetes with inadequate glycemic control (defined by the study authors). Trials in type 2 diabetes patients with concomitant diseases or risk factors will also be included, but these studies will be excluded in sensitivity analysis. Studies will be excluded if they involved patients with type 1 diabetes or patients without diabetes but insulin resistance.

Types of interventions

Trials have to include at least one novel anti-diabetic agent in the following classes: dipeptidyl peptidase 4 (DPP-4) inhibitors, glucagon-like peptide 1 (GLP-1) receptor agonists, and sodium-glucose–linked cotransporter 2 (SGLT-2) inhibitors. Furthermore, we also require that the involved anti-diabetic agent has been approved by either FDA or European Medicines Agency. Comparators can be placebo, metformin, sulfonylureas, thiazolidinediones, insulin, and another antidiabetic agent mentioned above. Trials that compared the different dosages or forms of the same agent will be excluded. It is also required that novel anti-diabetic agents were used with recommended dosages. For agents approved by FDA, we obtained the recommended dosages from dailymed.nlm.nih.gov. For agents approved by European Medicines Agency, recommended dosages were obtained from www.ema.europa.eu. Novel anti-diabetic agents that have been approved and their recommended dosages were summarized in Appendix 2. Trials that allowed for switching of treatments between groups will be excluded. There will be no strict limitation of traditional anti-diabetic agent dosages.

Types of studies

Double-blind, single-blind, and open-label RCTs comparing one novel anti-diabetic agent with their eligible comparator will be included. There is no limitation of baseline treatments as long as they are comparable in all of the study arms. Treatment durations have to be 24 weeks or longer. Trials with a crossover design will be excluded.

Outcomes of interest

To be included, studies have to report at least one event of the following outcomes.

(1) Major cardiovascular adverse events (MACE)

MACE is consisted of cardiovascular death, non-fatal myocardial infarction, non-fatal stroke, and unstable angina or hospitalization for unstable angina. Adverse event reported as acute coronary syndrome is also included in MACE.

1) Cardiovascular death includes adverse events reported as sudden cardiac death, sudden death of unknown reason, death due to myocardial infarction, death due to stroke, death due to heart failure or cardiogenic shock, and death due to cardiac arrest or cardiopulmonary arrest.

2) Non-fatal myocardial infarction includes non-fatal adverse events reported as myocardial infarction and acute myocardial infarction. Adverse events reported coronary artery disease, coronary artery occlusion, or myocardial ischemia will not be included. Silent myocardial infarction will not be included either.

3) Non-fatal stroke includes non-fatal adverse event reported as cerebrovascular accident, cerebral infarction, cerebellar infarction, brain stem infarction, lacunar infarction, stroke, ischemic stroke, hemorrhagic stroke, and subarachnoid hemorrhage. Adverse events reported as cerebral artery insufficiency, cerebral ischemia will not be included. Transient ischemic attack, epidural hematoma, and subdural hematoma will not be included either.

(2) All-cause mortality

All-cause mortality is defined as any death occurring during the treatment period.

(3) Heart failure

Heart failure includes adverse events reported as heart failure, cardiac failure, left/righ heart failure, left/right ventricular failure, left/right ventricular dysfunction, systolic/diastolic dysfunction.

**Search strategy**

Search strategy is described in Supplemental 3.

**Study selection and data extraction**

Two reviewers will independently scan the search results by reading the titles and abstracts. After excluding obviously ineligible reports, the reviewers will scan the remaining reports again by full-text reading. In order to determine whether the study reports any event of interested outcomes, data on www.clinicaltrials.gov will also be checked if the registry number is provided.

Two reviewers will then extract the data into a standard form. Data extracted will include outcomes of interest, study characteristics (registry number, the first author, whether it is international study, number of study centers, treatment duration), participant characteristic (mean age, concomitant high risk factor, proportion of male patients), and intervention details (type of agent and its dosage in each arm and baseline agent used across arms). For studies that used different terms from those mentioned in ‘Outcome of interest’ section to report cardiovascular events, it will be determined by reviewers’ discussion and, if necessary, a senior reviewer adjudication whether this event will be included into analysis.

**Quality assessment**

Two reviewers will independently assess methodological quality of included RCTs using the tool described in the Cochrane Collaboration Handbook^8^. Briefly, this tool includes 7 components, which are random sequence generation, allocation concealment, blinding of participants and personnel, blinding of outcome assessment, incomplete outcome data, selective reporting and other sources of bias. Each of these components of every included study will receive a rating of “Low risk”, “Unclear”, or “High risk”.

**Statistical analysis**

Stata version 14 package will be applied for statistical analyses, using the network and mvmeta command and Stata routines described elsewhere^9^.

For indirect and mixed comparisons, we will use network meta-analysis to obtain estimates for primary and secondary outcomes, and presented these estimates as odds ratios (dichotomous outcomes) with 95% CIs. We will then estimate the relative ranking probability of each treatment and obtained the treatment hierarchy of competing interventions using rankograms, surface under the cumulative ranking (SUCRA) curve, and mean ranks. Large SUCRA scores might indicate a more effective or safer intervention^10^.

To test the assumption of consistency in the entire network, we will use the design-by-treatment model of Higgins and colleagues, which provides a single inference, using the χ2 test and the restricted maximum likelihood method to estimate heterogeneity, assuming a common estimate for heterogeneity variance across different comparisons^11^. To check for the presence of inconsistency, we will use the loop-specific approach that assesses the difference between direct and indirect estimates for a specific comparison in the loop (inconsistency factor). We assume a common heterogeneity estimate within each loop. We will use the previously described node-splitting method, which separates evidence for a particular comparison into direct and indirect, excluding one direct comparison at a time and estimating the indirect treatment effect for the excluded comparison^12^. The codes that will be used in the analyses are described as followed:

1. Plotting network graphs of studies: *network map*

2. Performing network meta-analysis: *network meta c* for consistent models or *network meta i* for inconsistent models

3. Testing the consistency of the network: *intervalplot*

4. Testing the inconsistency of the network: *network sidesplit*

5. Calculating SUCRA and generating ranking: *network rank* and *sucra*

**Sensitivity analyses and meta-regression**

We will perform sensitivity analyses by excluding studies that involve patients with high cardiovascular risk, studies that involve patients with renal impairment, and studies with 1 arm involving less than 100 patients (data of remaining arms will be kept if they consist eligible comparison in multiple-arm studies). We will also perform meta-regression to adjust the effect of treatment duration and mean age.

**Investigation of reporting biases**

We will do a full evaluation of the association between study size and result by examining the comparison-adjusted funnel plots.

**References:**

**1.** **IDF**. IDF DIABETES ATLAS: http://www.idf.org/files/idf_publications/idf_diabetes_atlas_EN/idf_diabetes_atlas_EN/assets/common/downloads/publication.pdf (accessed May 8, 2015).

**2.** WHO. Global Health Estimates 2014 Summary Tables: http://www.who.int/healthinfo/global_burden_disease/en/ (accessed June 21, 2016).

**3.** **Sonesson C, Johansson PA, Johnsson E, Gause-Nilsson I.** Cardiovascular effects of dapagliflozin in patients with type 2 diabetes and different risk categories: a meta-analysis. Cardiovasc Diabetol. 2016;15:37.

**4.** **Fisher M, Petrie MC, Ambery PD, Donaldson J, Ye J, McMurray JJ.** Cardiovascular safety of albiglutide in the Harmony programme: a meta-analysis. Lancet Diabetes Endocrinol. 2015;3:697-703.

**5.** **Savarese G, D'Amore C, Federici M, De Martino F, Dellegrottaglie S, Marciano C, et al.** Effects of Dipeptidyl Peptidase 4 Inhibitors and Sodium-Glucose Linked coTransporter-2 Inhibitors on cardiovascular events in patients with type 2 diabetes mellitus: A meta-analysis. Int J Cardiol. 2016;220:595-601.

**6.** **Monami M, Dicembrini I, Nardini C, Fiordelli I, Mannucci E.** Effects of glucagon-like peptide-1 receptor agonists on cardiovascular risk: a meta-analysis of randomized clinical trials. Diabetes Obes Metab. 2014;16:38-47.

**7.** **Wu JH, Foote C, Blomster J, Toyama T, Perkovic V, Sundstrom J, et al.** Effects of sodium-glucose cotransporter-2 inhibitors on cardiovascular events, death, and major safety outcomes in adults with type 2 diabetes: a systematic review and meta-analysis. Lancet Diabetes Endocrinol. 2016;4:411-419.

**8.** Green S, Higgins JPT. *Cochrane handbook for systematic reviews of interventions*. Chichester, England: Wiley-Blackwell; 2008.

**9.** **White IR.** Multivariate random-effects meta-regression: Updates to mvmeta. Stata J. 2011;11:255-270.

**10.** **Salanti G, Ades AE, Ioannidis JPA.** Graphical methods and numerical summaries for presenting results from multiple-treatment meta-analysis: an overview and tutorial. J Clin Epidemiol. 2011;64:163-171.

**11.** **White IR, Barrett JK, Jackson D, Higgins JPT.** Consistency and inconsistency in network meta-analysis: model estimation using multivariate meta-regression. RESEARCH SYNTHESIS METHODS. 2012;3:111-125.

**12.** **Veroniki AA, Vasiliadis HS, Higgins JP, Salanti G.** Evaluation of inconsistency in networks of interventions. Int J Epidemiol. 2013;42:332-345.

# Additional file 1: S2. Search strategy

MEDLINE, EMBASE, Cochrane Library Central Register of Controlled Trials between Jan 1, 1980, and June 30, 2017 will be searched for identification of potential studies. We will also screen the reference lists of relevant reviews. Only articles published in English will be included. There will be no restriction of publication period. Conference abstracts will be excluded. In order to determine whether the study reported any event of interested outcomes, data on www.clinicaltrials.gov were also checked if the registry number was provided.

| **MEDLINE** | **Embase** |
| --- | --- |
| 1. exp Diabetes Mellitus Type 2/ 2. diabetes mellitus/ 3. non-insulin dependent diabetes mellitus 4. ((diabetes or diabetes mellitus or diabetic*) adj1 (type 2 or type ii or type ii or non-insulin dependent or noninsulin dependent or adult onset or mature onset or late onset)).tw) 5. NIDDD.tw. 6. or/1-4 7. glucagon like peptide 1 receptor agonist/ 8. (glp-1 or glp-1 receptor inhibitor* or glp-1 agonist*).tw. 9. albiglutide/ 10. dulaglutide/ 11. liraglutide/ 12. lixisenatide/ 13. albiglutide.tw. 14. dulaglutide.tw. 15. liraglutide.tw. 16. lixisenatide.tw. 17. dipeptidyl peptidase iv inhibitor/ 18. (dpp4 or dpp 4 or dpp iv).tw. 19. alogliptin/ 20. saxagliptin/ 21. sitagliptin/ 22. vildagliptin/ 23. alogliptin.tw. 24. saxagliptin.tw. 25. sitagliptin.tw. 26. vildagliptin.tw. 27. sodium glucose cotransporter 2 inhibitor/ 28. (sodium glucose cotransporter 2 inhibitor* or sodium glucose cotransporter ii inhibitor or sglt 2 inhibitor*).tw. 29. canagliflozin/ 30. dapagliflozin/ 31. empagliflozin/ 32. canagliflozin.tw. 33. dapagliflozin.tw. 34. empagliflozin.tw. 35. antidiabetic agent/ 36. oral antidiabetic agent/ 37. exp insulin, Long acting/ 38. ((long acting or longer acting or intermediate acting) adj insulin*).tw. 39. insulin degludec/ 40. insulin detemir/ 41. insulin glargine/ 42. insulin zinc suspension 43. insulin aspart/ 44. insulin lispro/ 45. isophane insulin/ 46. meglitinide/ 47. mitiglinide/ 48. nateglinide/ 49. repaglinide/ 50. amylin derivative/ 51. pramlintide/ 52. biguanide derivative/ 53. metformin.tw. 54. sulphonylurea*.tw. 55. acetohexamide/ 56. carbutamide/ 57. chlorpropamide/ 58. glibornuride/ 59. glibenclamide/ 60. gliclazide/ 61. glimepiride.tw. 62. glipizide/ 63. gliquidone/ 64. tolazamide/ 65. acetohexamide.tw. 66. carbutamide.tw. 67. chlorpropamide.tw. 68. glibenclamide.tw. 69. gliclazide.tw. 70. glyburide.tw. 71. glitazone derivative/ 72. thiazolidinedione*.tw. 73. pioglitazone/ 74. rivoglitazone/ 75. rosiglitazone/ 76. pioglitazone.tw. 77. rivoglitazone.tw. 78. rosiglitazone.tw. 79. or/6-77 80. and/5,78 81. randomized controlled trial.pt. 82. controlled clinical trial.pt. 83. pragmatic clinical trial.pt. 84. double-blind.ab. 85. single-blind.ab. 86. randomized.ab. 87. placebo.ab. 88. trial.ti. 89. or/81-88 90. animals/not(humans/and animal/) 91. 89 not 90 | 1. diabetes mellitus/ 2. non-insulin dependent diabetes mellitus 3. ((diabetes or diabetes mellitus or diabetic*) adj1 (type 2 or type ii or type ii or non-insulin dependent or noninsulin dependent or adult onset or mature onset or late onset)).tw) 4. NIDDD.tw. 5. or/1-4 6. glucagon like peptide 1 receptor agonist/ 7. (glp-1 or glp-1 receptor inhibitor* or glp-1 agonist*).tw. 8. albiglutide/ 9. dulaglutide/ 10. liraglutide/ 11. lixisenatide/ 12. albiglutide.tw. 13. dulaglutide.tw. 14. liraglutide.tw. 15. lixisenatide.tw. 16. dipeptidyl peptidase iv inhibitor/ 17. (dpp4 or dpp 4 or dpp iv).tw. 18. alogliptin/ 19. saxagliptin/ 20. sitagliptin/ 21. vildagliptin/ 22. alogliptin.tw. 23. saxagliptin.tw. 24. sitagliptin.tw. 25. vildagliptin.tw. 26. sodium glucose cotransporter 2 inhibitor/ 27. (sodium glucose cotransporter 2 inhibitor* or sodium glucose cotransporter ii inhibitor or sglt 2 inhibitor*).tw. 28. canagliflozin/ 29. dapagliflozin/ 30. empagliflozin/ 31. canagliflozin.tw. 32. dapagliflozin.tw. 33. empagliflozin.tw. 34. antidiabetic agent/ 35. oral antidiabetic agent/ 36. long acting insulin/ 37. ((long acting or longer acting or intermediate acting) adj insulin*).tw. 38. insulin degludec/ 39. insulin detemir/ 40. insulin glargine/ 41. insulin zinc suspension 42. insulin aspart/ 43. insulin lispro/ 44. isophane insulin/ 45. meglitinide/ 46. mitiglinide/ 47. nateglinide/ 48. repaglinide/ 49. amylin derivative/ 50. pramlintide/ 51. biguanide derivative/ 52. metformin.tw. 53. sulphonylurea*.tw. 54. acetohexamide/ 55. carbutamide/ 56. chlorpropamide/ 57. glibornuride/ 58. glibenclamide/ 59. gliclazide/ 60. glimepiride.tw. 61. glipizide/ 62. gliquidone/ 63. tolazamide/ 64. acetohexamide.tw. 65. carbutamide.tw. 66. chlorpropamide.tw. 67. glibenclamide.tw. 68. gliclazide.tw. 69. glyburide.tw. 70. glitazone derivative/ 71. thiazolidinedione*.tw. 72. pioglitazone/ 73. rivoglitazone/ 74. rosiglitazone/ 75. pioglitazone.tw. 76. rivoglitazone.tw. 77. rosiglitazone.tw. 78. or/6-77 79. and/5,78 80. randomized controlled trial/ 81. double-blind/ 82. single-blind/ 83. random$.tw. 84. factorial$.tw. 85. assign$.tw. 86. allocate$.tw. 87. or/80-86 88. and/79,87 89. MEDLINE.cr 90. 88 not 89 91. (mouse or mice or murine or rat or rats or dog or animal*).ti. 92. 90 not 91 |

# Additional file 1: S3. Novel drugs approved by FDA or European Medicines Agency

| **Drugs** | **Recommended Dosage** | **Dosage in Patients with Renal Impairment** |
| --- | --- | --- |
| **DPP-4 inhibitors** | | |
| Sitagliptin  (JANUVIA) | 100 mg once daily | Mild: no dosage adjustment  Moderate: 50 mg once daily  Severe or end-stage: 25 mg once daily |
| Saxagliptin (ONGLYZA) | 2.5 mg or 5 mg once daily | Mild: no dosage adjustment  Moderate, severe or end-stage: 2.5 mg once daily |
| Vildagliptin  (Galvus^*^) | 100 mg once daily or 50mg twice daily  50 mg once daily if combined use with a sulphonylurea | Mild: no dosage adjustment  Moderate, severe or end-stage: 50 mg once daily |
| Alogliptin  (NESINA) | 25 mg once daily | Mild: no dosage adjustment  Moderate: 12.5mg once daily  Severe or end-stage: 6.25 mg once daily |
| Linagliptin (TRADJENTA) | 5 mg once daily | No dosage adjustment |
| **GLP-1 receptor agonists** | | |
| Liraglutide  (VICTOZA) | 1.2 mg once daily  1.8 mg once daily for inadequate glycemic control | No dosage adjustment |
| Dulaglutide (TRULICITY) | 0.75 mg once weekly  1.5 mg once weekly for inadequate glycemic control | No dosage adjustment |
| Albiglutide (TANZEUM) | 30 mg once weekly  50 mg once weekly for inadequate glycemic control | No dosage adjustment |
| Lixisenatide (LYXUMIA^*^) | 20 μg once daily | Mild and moderate: No dosage adjustment  Severe or end-stage: not recommended |
| Exenatide (BYDUREON) | 2mg once weekly | Moderate: use with caution  Severe or end-stage: not recommended |
| Exenatide  (BYETTA) | 5 μg bid or 10 μg bid based on clinical response | Moderate: use with caution  Severe or end-stage: not recommended |
| **SGLT-2 inhibitors** | | |
| Empagliflozin (JARDIANCE) | 10 mg once daily  25 mg once daily if JARDIANCE is tolerated | eGFR > 45 mL/min/1.73 m2: No dosage adjustment  eGFR < 45 mL/min/1.73 m2: Not recommended |
| Dapagliflozin (FARXIGA) | 5 mg once daily  10 mg once daily if FARXIGA is tolerated | eGFR > 60 mL/min/1.73 m2: No dosage adjustment  eGFR < 60 mL/min/1.73 m2: Not recommended |
| Canagliflozin (INVOKANA) | 100 mg once daily  300 mg once daily if INVOKANA is tolerated | eGFR 45~60 mL/min/1.73 m2: 100 mg once daily  eGFR < 45 mL/min/1.73 m2: Not recommended |
| ^*^ Still not approved by FDA, but already approved by the European Medicines Agency | | |

# Additional file 1: S4. Flow chart of the study selection process


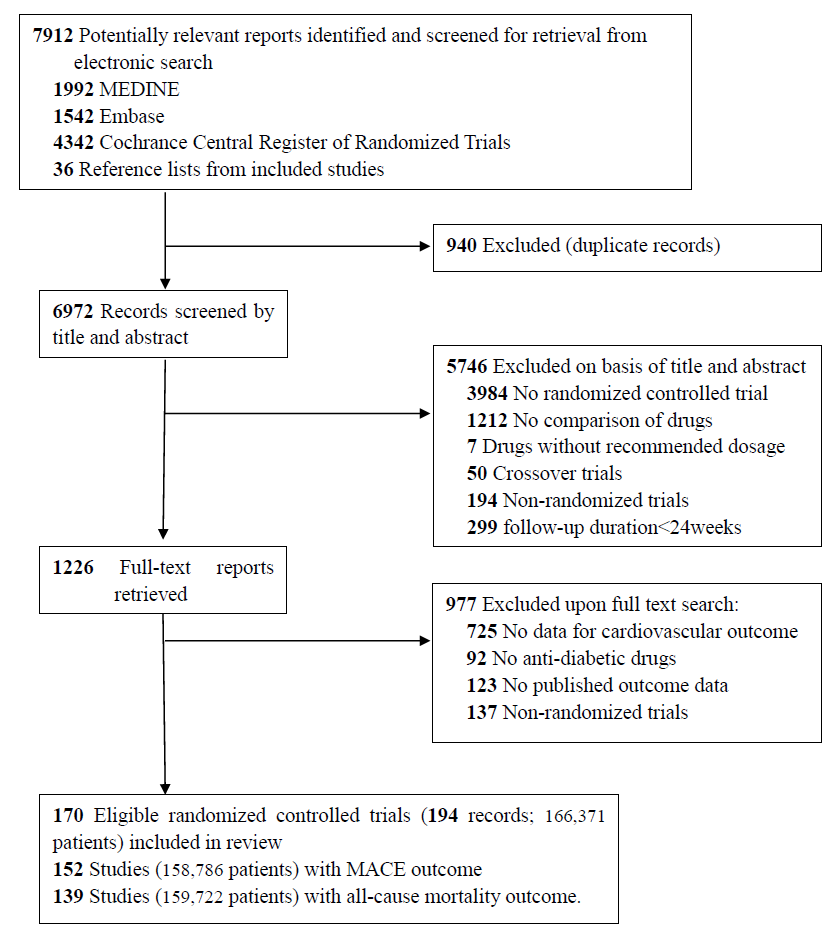


# Additional file 1: S5. Studies characteristic of the included studies

| Study | International | Centers | Special character of population | Antidabetic | Comparator | Drug used across groups | Treatment duration (weeks) | Mean age (years) | Male Patients (%) |
| --- | --- | --- | --- | --- | --- | --- | --- | --- | --- |
| NCT00968708^1, 2^ | Yes | 898 | High cardiovascular risk | Alogliptin | Placebo | OAD or Insulin | 76(Mean) | 61^#^ | 67.9 |
| NCT00856284^3^ | Yes | 310 | None | Alogliptin | Glipizide | Metformin | 104 | 55.5 | 50.8 |
| NCT01023581^4^ | Yes | 198 | None | Alogliptin | Metformin,Placebo | None | 26 | 53.2 | 48.2 |
| NCT00707993^5^ | Yes | 110 | Age≥65 | Alogliptin | Glipizide | None | 52 | 70.0 | 44.9 |
| NCT00328627^6^ | Yes | 327 | None | Alogliptin | Pioglitazone, Placebo | Metformin | 26 | 54.7 | 44.5 |
| NCT00432276^7^ | Yes | NR | None | Alogliptin | Pioglitazone | Metformin + Pioglitazone | 52 | 55.1 | 51.6 |
| NCT01318070^8^ | No(Japan) | 33 | None | Alogliptin | Placebo | Pioglitazone | 52 | 59.7 | 64.0 |
| NCT00286442^9^ | Yes | 115 | None | Alogliptin | Placebo | Metformin | 26 | 55.0 | 52.2 |
| NCT00286494^10^ | Yes | 125 | None | Alogliptin | Placebo | Pioglitazone | 26 | 55.3 | 60.1 |
| NCT00286429^11^ | Yes | 110 | None | Alogliptin | Placebo | Insulin ± Metformin | 26 | 55.4 | 40.9 |
| NCT00395512^12^ | NR | NR | None | Alogliptin | Pioglitazone | None | 26 | 53.0 | 48.9 |
| NCT00996658^12^ | Yes | 52 | None | Linagliptin | Placebo | Metformin + Pioglitazone | 24 | 53.8 | 48.5 |
| NCT01084005^14^ | Yes | 33 | Age≥70 | Linagliptin | Placebo | OAD or Insulin | 24 | 74.9 | 68.5 |
| NCT00800683^15^ | Yes | 53 | Severe renal impairment | Linagliptin | Placebo | OAD or Insulin | 52 | 64.4 | 60.2 |
| NCT00954447^16^ | Yes | 167 | None | Linagliptin | Placebo | Insulin ±Metformin/Pioglitazone | 52 | 60.0 | 52.1 |
| NCT00622284^17^ | Yes | 209 | None | Linagliptin | Glimepiride | Metformin | 104 | 59.8 | 60.0 |
| NCT00641043^18^ | Yes | 43 | None | Linagliptin | Placebo | Pioglitazone | 24 | 57.5 | 60.9 |
| NCT00798161^19^ | Yes | 133 | None | Linagliptin | Metformin, Placebo | None | 24 | 54.9 | 54.7 |
| NCT01422876^20, 21*^ | Yes | 197 | None | Linagliptin, Empagliflozin |  | None or Metformin | 52 | 54.6 | 54.3 |
| NCT01204294^22^ | No(Japan) | 43 | None | Linagliptin | Metformin | Sulfonylurea or α-glucosidase inhibitors | 52 | 60.5 | 60.8 |
| NCT00602472^23^ | Yes | 100 | None | Linagliptin | Placebo | Meformin + Sulfonylurea | 24 | 58.1 | 47.2 |
| NCT00601250^24^ | Yes | 82 | None | Linagliptin | Placebo | Metformin | 24 | 56.5 | 54.0 |
| NCT01006603^25^ | Yes | 152 | Age≥65 | Saxagliptin | Glimepiride | Metformin | 52 | 72.6 | 61.8 |
| NCT00757588^26, 27^ | Yes | 72 | None | Saxagliptin | Placebo | Insulin ± Metformin | 52 | 57.2 | 41.3 |
| NCT00575588^28, 29^ | Yes | NR | None | Saxagliptin | Glipizide | Metformin | 104 | 57.5 | 51.6 |
| NCT00121641^30, 31^ | NR | NR | None | Saxagliptin | Placebo | None | 208 | 53.7 | 52.5 |
| NCT01107886^32^ | Yes | 788 | High cardiovascular risk | Saxagliptin | Placebo | OAD or Insulin | 104(Median) | 65.1 | 64.9 |
| NCT00698932^33^ | Yes | 40 | None | Saxagliptin | Placebo | None | 24 | 51.4 | 55.5 |
| NCT00661362^34^ | Yes | 40 | None | Saxagliptin | Placebo | Metformin | 24 | 53.1 | 48.3 |
| NCT00313313^35^ | Yes | NR | None | Saxagliptin | Glyburide | Glyburide | 24 | 55.1 | 45.1 |
| NCT00121667^36^ | Yes | NR | None | Saxagliptin | Placebo | Metformin | 24 | 54.7 | 50.2 |
| NCT00295633^37, 38^ | NR | 172 | None | Saxagliptin | Placebo | Thiazolidinedione | 76 | 54.0 | 49.6 |
| NCT00327015^39, 40^ | Yes | NR | None | Saxagliptin | Placebo | Metformin | 76 | 52.5 | 51.2 |
| NCT00614939^41^ | Yes | NR | Renal impairment | Saxagliptin | Placebo | OAD or Insulin | 52 | 66.5 | 42.9 |
| NCT01006590^42^ | Yes | NR | None | Saxagliptin | Metformin | Metformin | 24 | 58.7 | 57.3 |
| NCT01289990^43, 44^(1) | Yes | 124 | None | Sitagliptin, Empagliflozin | Placebo | None | 76 | 55.0 | 61.0 |
| NCT00790205^45^ | Yes | 673 | High cardiovascular risk | Sitagliptin | Placebo | OAD or Insulin | 144(Mean) | 65.5 | 70.7 |
| NCT00722371^46^ | NR | NR | None | Sitagliptin | Pioglitazone | None | 54 | 51.4 | 46.6 |
| NCT01098539^47^ | Yes | 134 | Renal impairment | Sitagliptin, Albiglutide |  | OAD | 52 | 63.3 | 53.7 |
| NCT00734474^48, 49^ | Yes | 111 | None | Sitagliptin, Dulaglutide |  | Metformin | 104 | 54.0 | 46.8 |
| NCT01137812^50^ | Yes | 140 | None | Sitagliptin, Canagliflozin |  | Metformin + Sulfonylurea | 52 | 56.7 | 55.9 |
| NCT01177813^51^ | Yes | 124 | None | Sitagliptin, Empagliflozin | Placebo | None | 24 | 55.0 | 61.0 |
| NCT00509236^52^ | Yes | 31 | Severe renal impairment | Sitagliptin | Glipizide | None | 54 | 60.0 | 59.7 |
| NCT00509262^53^ | Yes | NR | Moderate to severe renal impairment | Sitagliptin | Glipizide | None | 54 | 64.5 | 57.0 |
| NCT00350779^54^ | Yes | 41 | None | Sitagliptin | Placebo | Metformin + Rosiglitazone | 54 | 54.5 | 42.4 |
| NCT00885352^55^ | Yes | 58 | None | Sitagliptin | Placebo | Metformin + Pioglitazone | 26 | 56.1 | 62.3 |
| NCT01046110^56^ | Yes | 78 | None | Sitagliptin | Insulin degludec | OAD | 26 | 55.7 | 58.6 |
| NCT00751114^57^ | Yes | NR | None | Sitagliptin | Insulin glargine | Metformin | 24 | 53.6 | 51.0 |
| NCT00701090^58^ | Yes | NR | None | Sitagliptin | Glimepiride | Metformin | 30 | 56.2 | 54.4 |
| NCT00700817^59, 60^ | Yes | 158 | None | Sitagliptin, Liraglutide |  | Metformin | 52 | 55.3 | 53.0 |
| NCT00637273^61^ | Yes | 72 | None | Sitagliptin, Exenatide | Pioglitazone | Metformin | 26 | 52.0 | 51.7 |
| NCT00094770^62, 63^ | Yes | NR | None | Sitagliptin | Glipizide | Metformin | 104 | 56.7 | 59.2 |
| NCT00395343^64^ | Yes | 100 | None | Sitagliptin | Placebo | Insulin | 24 | 57.8 | 50.9 |
| NCT00337610^65^ | Yes | NR | None | Sitagliptin | Placebo | Metformin | 30 | 54.9 | 53.7 |
| NCT00103857^66-68^（1） | Yes | 117 | None | Sitagliptin | Metformin | None | 104 | 53.3 | 48.6 |
| NCT00103857^66-68^（2） | Yes | 140 | None | Sitagliptin | Placebo | None | 24 | 53.4 | 52.4 |
| NCT01189890^69^ | Yes | 85 | Age≥65 | Sitagliptin | Glimepiride | None | 30 | 70.7 | 43.8 |
| NCT00838903^70^ | Yes | 289 | None | Sitagliptin, Albiglutide | Placebo, Glimepiride | Metformin | 156 | 54.5 | 47.6 |
| NCT01106677^71^ | Yes | 169 | None | Sitagliptin, Canagliflozin | Canagliflozin | Metformin | 52 | 55.4 | 46.4 |
| NCT00676338^72^ | Yes | NR | None | Sitagliptin, Exenatide, | Metformin, Pioglitazone | None | 26 | 54.0 | 59.0 |
| NCT00397631^73^ | Yes | NR | None | Sitagliptin | Placebo | Pioglitazone | 24 | 50.9 | 54.2 |
| NCT00449930^74^ | Yes | 113 | None | Sitagliptin | Metformin | None | 24 | 56.0 | 56.0 |
| NCT00106704^75^ | Yes | NR | None | Sitagliptin | Placebo | Glimepiride ± Metformin | 24 | 56.1 | 53.1 |
| NCT00881530^76^（1） | Yes | 132 | None | Sitagliptin, Empagliflozin |  | Metformin | 78 | 59.0^#^ | 49.4 |
| NCT01357252^77^ | Yes | NR | None | Vildagliptin | Placebo | Glimepiride | 24 | 58.5 | 56.6 |
| NCT01257451^78^ | Yes | 43 | Age≥70 | Vildagliptin | Placebo | None or OAD | 24 | 74.8 | 45.3 |
| 2012Pan^79^ | No(China) | NR | None | Vildagliptin | Placebo | Metformin | 24 | 54.3 | 47.9 |
| NCT00237237^80, 81^ | Yes | NR | None | Vildagliptin | Pioglitazone | Metformin | 52 | 56.6 | 63.0 |
| NCT00382096 and NCT00468039^82^ | Yes | 250 | None | Vildagliptin | Metformin | None | 24 | 59.1 | 53.0 |
| NCT00106340^83, 84^ | Yes | 402 | None | Vildagliptin | Glimepiride | Metformin | 52 | 57.5 | 53.4 |
| NCT00383578^85^ | Yes | 113 | Age≥65 | Vildagliptin | Metformin | None | 24 | 70.9 | 49.1 |
| NCT00099892^86^ | Yes | 109 | None | Vildagliptin | Placebo | Metformin | 24 | 54.2 | 57.3 |
| NCT00099931^87^ | Yes | 68 | None | Vildagliptin | Placebo | Insulin | 24 | 59.2 | 51.4 |
| NCT00099866^88^ | Yes | 183 | None | Vildagliptin | Metformin | None | 52 | 53.2 | 55.0 |
| NCT01649466^89^ | Yes | 47 | None | Vildagliptin | NPH insulin | Sulfonylurea | 24 | 66.7 | 55.0 |
| 2013Kothny^90^ | NR | NR | None | Vildagliptin | Placebo | Insulin ± Metformin | 24 | 59.2 | 49.9 |
| 2011Lukashevich^91^ | NR | NR | Renal impairment | Vildagliptin | Placebo | None,OAD or Insulin | 24 | 66.7 | 57.3 |
| NCT00102388^92^ | Yes | 151 | None | Vildagliptin | Gliclazide | None | 104 | 54.7 | 55.8 |
| NCT00099918^93^ | Yes | 202 | None | Vildagliptin | Rosiglitazone | None | 24 | 54.4 | 57.5 |
| NCT00849017^94^ | Yes | 143 | None | Albiglutide | Placebo | None | 156 | 52.9 | 55.1 |
| NCT00839527^95^ | Yes | 234 | None | Albiglutide | Pioglitazone, Placebo | Metformin and Glimepiride | 156 | 55.2 | 53.2 |
| NCT00849056^96^ | Yes | 158 | None | Albiglutide | Placebo | Pioglitazone ± Metformin | 156 | 55.0 | 59.8 |
| NCT00976391^97^ | NR | NR | None | Albiglutide | Insulin lispro | Insulin glargine ± OAD | 26 | 55.6 | 47.0 |
| NCT00838916^98^ | Yes | 222 | None | Albiglutide | Insulin glargine | Metformin ± Sulfonylurea | 52 | 55.5 | 56.1 |
| NCT01128894^99^ | Yes | 162 | None | Albiglutide, Liraglutide |  | None | 32 | 55.6 | 50.0 |
| NCT01191268^100^ | Yes | 105 | None | Dulaglutide | Insulin glargine | Metformin | 52 | 59.0 | 53.5 |
| NCT01149421^101^ | Yes | 76 | None | Dulaglutide | Placebo | Insulin | 26 | 56.5 | 51.9 |
| NCT01126580^102^ | Yes | NR | None | Dulaglutide | Metformin | OAD | 52 | 44.0 | 56.0 |
| NCT01064687^103^ | Yes | NR | None | Dulaglutide, Exenatide | Placebo | None | 26 | 56.0 | 55.6 |
| NCT00765817^104, 105^ | Yes | 59 | None | Exenatide | Placebo | OAD | 30 | 59.0 | 57.1 |
| NCT00359762^106, 107^ | Yes | 128 | None | Exenatide | Glimepiride | Insulin glargine | 108(Mean) | 57.2 | 53.8 |
| NCT01147627^108^ | Yes | 25 | None | Exenatide | Insulin lispro 75/25, Pioglitazone | Metformin | 48 | 50.3 | 50.2 |
| NCT00960661^109^ | Yes | 108 | None | Exenatide | Insulin lispro | None | 30 | 59.7 | 48.0 |
| NCT00641056^110-112^ | Yes | 72 | None | Exenatide | Insulin glargine | Insulin glargine + Metformin | 26 | 58.0 | 53.3 |
| NCT00082407^113^ | Yes | NR | None | Exenatide | Insulin aspart | Metformin ± Sulfonylurea | 52 | 59.0 | 51.0 |
| NCT00935532^114^ | Yes | NR | None | Exenatide | Insulin glargine | Metformin + Sulfonylurea | 26 | 56.8 | 67.9 |
| NCT00434954^115^ | Yes | 68 | None | Exenatide | Insulin aspart 70/30 | OAD | 26 | 57.0 | 57.6 |
| NCT00603239^116^ | Yes | 28 | None | Exenatide | Placebo | Metformin ± Sulfonylurea | 26 | 55.0 | 59.4 |
| NCT00097877^117^ | Yes | 102 | None | Exenatide | Insulin aspart 70/30 | Thiazolidinedione ±Metformin | 24 | 52.6 | 48.1 |
| 2009Davies^118^ | Yes | 36 | High cardiovascular risk | Exenatide | Insulin glargine | Metformin + Sulfonylurea | 26 | 56.5 | 68.4 |
| 2004Buse^119^ | Yes | 101 | None | Exenatide | Placebo | OAD | 30 | 55.0 | 75.0 |
| NCT01029886^120^ | Yes | 105 | None | Exenatide, Liraglutide |  | Sulfonylurea | 26 | 57.0 | 55.0 |
| NCT00518882^121^ | Yes | 132 | None | Exenatide, Liraglutide |  | None or OAD | 26 | 56.7 | 52.0 |
| NCT01179048^122^ | Yes | 410 | High cardiovascular risk | Liraglutide | Placebo | OAD | 182(Median) | 64.3 | 64.3 |
| NCT01117350^123^ | Yes | NR | None | Liraglutide | Insulin glargine | None, OAD or Insulin | 24 | 57.3 | 55.9 |
| NCT01336023^124^ | Yes | 271 | None | Liraglutide | Insulin degludec | Insulin | 26 | 55.0 | 49.0 |
| NCT01388361^125^ | Yes | 119 | None | Liraglutide | Insulin aspart | Metformin ± Pioglitazone | 26 | 60.4 | 65.6 |
| NCT00294723^126-128^ | Yes | 138 | None | Liraglutide | Glimepiride | Insulin degludec + Metformin | 104 | 53.0 | 50.0 |
| NCT00318461^129^ | Yes | 170 | None | Liraglutide | Placebo, Glimepiride | None | 26 | 57.0 | 57.0 |
| NCT01272232^130^ | Yes | 126 | None | Liraglutide | Placebo | Metformin | 56 | 54.8 | 48.5 |
| NCT01147250^131^ | Yes | NR | High cardiovascular risk | Lixisenatide | Placebo | None or OAD | 100(Median) | 60.3 | 69.3 |
| NCT00713830^132^ | NR | NR | None | Lixisenatide | Placebo | None,OAD or Insulin | 24 | 57.3 | 50.5 |
| NCT01169779^133^ | Yes | 37 | None | Lixisenatide | Placebo | Sulfonylurea ± Metformin | 24 | 54.8 | 49.2 |
| NCT00763815^134^ | Yes | 150 | None | Lixisenatide | Placebo | Metformin ± Sulfonylurea | 24 | 55.8 | 47.0 |
| NCT00715624^135^ | Yes | 111 | None | Lixisenatide | Placebo | Pioglitazone ± Metformin | 24 | 57.0 | 46.0 |
| NCT00975286^136^ | Yes | 140 | None | Lixisenatide | Placebo | Insulin ± Metformin | 24 | 56.0 | 50.0 |
| NCT00866658^137^ | Yes | 57 | None | Lixisenatide | Placebo | Insulin+Metformin ± Thiazolidinedione | 24 | 58.3 | 47.9 |
| NCT00763451^138^ | Yes | 75 | None | Lixisenatide | Placebo | Insulin ± Sulfonylurea | 72 | 56.1 | 45.0 |
| NCT00707031^139^ | Yes | 122 | None | Lixisenatide, Exenatide |  | Metformin | 24 | 57.4 | 53.5 |
| NCT01106651^140, 141^ | Yes | 90 | Age≥55 | Canagliflozin | Placebo | Metformin | 104 | 63.6 | 55.5 |
| NCT00968812^142, 143^ | Yes | 157 | None | Canagliflozin | Glimepiride | None,OAD or Insulin | 104 | 56.2 | 52.0 |
| NCT01081834^144, 145^ | Yes | NR | None | Canagliflozin | Placebo | Metformin | 26 | 55.4 | 44.2 |
| NCT01064414^146, 147^ | NR | NR | Renal impairment | Canagliflozin | Placebo | None or OAD | 52 | 68.5 | 60.6 |
| NCT01106625^148^ | Yes | 85 | None | Canagliflozin | Placebo | None,OAD or Insulin | 52 | 56.8 | 51.0 |
| NCT01106690^149^ | Yes | 72 | None | Canagliflozin | Placebo | Metformin + Sulfonylurea | 26 | 57.4 | 63.2 |
| NCT01032629^150^ | Yes | 386 | High cardiovascular risk | Canagliflozin | Placebo | Metformin + Pioglitazone | 52 | 63.0^#^ | 66.0 |
| NCT00528372^151, 152^ | Yes | 85 | None | Dapagliflozin | Placebo | Insulin | 102 | 52.2 | 46.5 |
| NCT01031680^153^ | Yes | NR | High cardiovascular risk | Dapagliflozin | Placebo | None | 52 | 62.9 | 68.3 |
| NCT00660907^154-156^ | Yes | 95 | None | Dapagliflozin | Glipizide | OAD or Insulin | 52 | 59.0 | 55.1 |
| NCT00984867^157^ | Yes | NR | None | Dapagliflozin | Placebo | Metformin | 48 | 54.9 | 54.9 |
| NCT00663260^158^ | Yes | 111 | Renal impairment | Dapagliflozin | Placebo | Sitagliptin ± Metformin | 104 | 67.0 | 55.0 |
| NCT01042977^159^ | Yes | 173 | High cardiovascular risk | Dapagliflozin | Placebo | None, OAD or Insulin | 52 | 63.7 | 67.0 |
| NCT00673231^160, 161^ | Yes | 126 | None | Dapagliflozin | Placebo | OAD or Insulin | 104 | 59.1 | 46.2 |
| NCT00528879^162, 163^ | Yes | 80 | None | Dapagliflozin | Placebo | OAD or Insulin | 102 | 53.6 | 54.3 |
| NCT00643851^164^ | Yes | 105 | None | Dapagliflozin | Metformin XR | None | 24 | 52.1 | 53.7 |
| NCT00859898^164^ | Yes | 131 | None | Dapagliflozin | Metformin XR | None | 24 | 51.9 | 52.7 |
| NCT00855166^165^ | Yes | 40 | None | Dapagliflozin | Placebo | None | 50 | 60.7 | 55.6 |
| NCT00683878^166^ | Yes | 105 | None | Dapagliflozin | Placebo | Metformin | 48 | 53.5 | 49.5 |
| NCT00680745^167^ | Yes | 84 | None | Dapagliflozin | Placebo | Pioglitazone | 24 | 59.8 | 52.0 |
| NCT01131676^168^ | Yes | 590 | High cardiovascular risk | Empagliflozin | Placebo | Pioglitazone and Metformin | 135(Median) | 63.1 | 71.4 |
| NCT01210001^169^ | Yes | 69 | None | Empagliflozin | Placebo | OAD or Insulin | 76 | 54.5 | 48.4 |
| NCT01368081^170^ | No(Japan) | 86 | None | Empagliflozin | Metformin | None or Pioglitazone ± Metformin | 52 | 61.2 | 71.4 |
| NCT01164501^171^ | Yes | 127 | Renal impairment | Empagliflozin | Placebo | Sulfonylurea | 52 | 64.7 | 57.8 |
| NCT01289990^43, 44^（2）^*^ | Yes | 148 | None | Empagliflozin | Placebo | OAD or Insulin | 76 | 56.4 | 53.7 |
| NCT01167881^172^ | Yes | 173 | None | Empagliflozin | Glimepiride | None or OAD | 104 | 56.0 | 55.0 |
| NCT01306214^173^ | Yes | 104 | None | Empagliflozin | Placebo | Metformin | 52 | 56.7 | 45.0 |
| NCT00881530^76^（2） | Yes | 132 | None | Empagliflozin | Metformin | Insulin ± Metformin | 78 | 59^#^ | 49.4 |
| NCT00521742^174^ | No(USA) | NR | Early heart failure | Glyburide | Pioglitazone | None | 52 | 64 | 56 |
| NCT00379769^175^ | Yes | 338 | None | Glyburide, Gliclazide, or Glimepiride | Rosiglitazone | Metformin | 195 | 58.6 | 51.5 |
| NCT00225264^176^ | No(USA) | 28 | None | Glimepiride | Pioglitazone | None, OAD or Insulin | 72 | 59.6 | 63.1 |
| 1998UKPDS^177^ | No(UK) | 23 | None | Glibenclamide | Insulin | None | 520 | 54 | 62.2 |
| 2008Hamann^178^ | Yes | 118 | None | Glibenclamide or Gliclazide | Rosiglitazone | Metformin | 52 | 58.9 | 52 |
| NCT00513630^179^ | No(China) | 15 | Coronary heart disease | Glipizide | Metformin | None | 260 | 63.3 | 77.6 |
| NCT00484198^180^ | Yes | 254 | None | Pioglitazone | Placebo | None or OAD | 26 | 55.1 | 52.4 |
| NCT00116831^181^ | Yes | 92 | Coronary heart disease | Glipizide | Rosiglitazone | None | 78 | 61.0 | 67.9 |
| NCT00494312^182^ | No(USA) | 171 | None | Glibenclamide | Pioglitazone | None or Metformin | 144 | 54^#^ | 56.4 |
| NCT00225277^183^ | Yes | 97 | Coronary heart diesase | Glimepiride | Pioglitazone | None | 156 | 59.8 | 67.4 |
| 2006Bakris^184^ | Yes | 88 | None | Glyburide | Rosiglitazone | Metformin | 32 | 59.4 | 66 |
| 2006Jain^185^ | No(USA) | 65 | None | Glyburide | Pioglitazone | None | 56 | 52.1 | 54.6 |
| NCT00279045^186^ | Yes | 488 | None | Glyburide, Rosiglitazone | Metformin | None | 408(median) | 56.9 | 57.7 |
| 2006Rosenstock^187^ | Yes | 90 | None | Rosiglitazone | Metformin | None | 32 | 51.0 | 57 |
| 2005Charbonnel^188^ | Yes | 91 | None | Pioglitazone | Metformin | Sulfonylurea | 104 | 60 | 54.1 |
| 2004Dailay^189^ | No(USA) | 61 | None | Rosiglitazone | Placebo | Metformin + Glyburide | 24 | 57 | 60 |
| 2004Schernthaner^190^ | Yes | 167 | None | Pioglitazone | Metformin | None | 52 | 56.5 | 55.2 |
| 2000Fonseca^191^ | No(USA) | 36 | None | Rosiglitazone | Placebo | None | 26 | 58.2 | 68.2 |
| 2000Horton^192^ | NR | NR | None | Metformin | Placebo | None | 24 | 58.2 | 64.3 |
| 1998Johnston^193^ | No(USA) | 30 | None | Glyburide | Placebo | None | 56 | 68.1 | 63 |
| 1995DeFronzo^194^ | No(USA) | >1 | None | Glyburide | Metformin | None | 29 | 55 | 47.5 |
| ^*^ Baseline information was only available for a part of the population; ^#^ Mean was estimated by median; ± with or without | | | | | | | | | |

# Additional file 1: S6. Outcomes of interest in each study

| Study | Arm | Total number | MACE | Cardiovascular death | Non-fatal myocardial infarction | Non-fatal stroke | Unstable angina | Acute coronary syndrome | All-cause mortality | Severe hypoglycemia |
| --- | --- | --- | --- | --- | --- | --- | --- | --- | --- | --- |
| NCT00968708^1, 2^ | Alogliptin | 2701 | 344 | 89 | 187 | 29 | 43 | 0 | 153 | 18 |
|  | Placebo | 2679 | 359 | 111 | 173 | 32 | 47 | 0 | 173 | 16 |
| NCT00856284^3^ | Alogliptin | 878 | 8 | 2 | 4 | 2 | 0 | 0 | 3 | 0 |
|  | Sulfonylurea | 869 | 12 | 4 | 4 | 3 | 0 | 1 | 5 | 5 |
| NCT01023581^4^ | Alogliptin | 112 | 1 | 0 | 1 | 0 | 0 | 0 | 0 | 0 |
|  | Metformin | 220 | 0 | 0 | 0 | 0 | 0 | 0 | 0 | 0 |
|  | Placebo | 106 | 0 | 0 | 0 | 0 | 0 | 0 | 0 | 0 |
| NCT00707993^5^ | Alogliptin | 222 | 1 | 0 | 0 | 0 | 1 | 0 | 0 | 0 |
|  | Sulfonylurea | 219 | 2 | 0 | 1 | 1 | 0 | 0 | 0 | 3 |
| NCT00328627^6^ | Alogliptin | 129 | 0 | 0 | 0 | 0 | 0 | 0 | 0 | NR |
|  | Thiazolidinedione | 388 | 1 | 0 | 0 | 1 | 0 | 0 | 1 | NR |
|  | Placebo | 129 | 1 | 0 | 0 | 1 | 0 | 0 | 0 | NR |
| NCT00432276^7^ | Alogliptin | 404 | 3 | 1 | 0 | 1 | 1 | 0 | 1 | 2 |
|  | Thiazolidinedione | 399 | 4 | 0 | 2 | 1 | 1 | 0 | 0 | 0 |
| NCT01318070^8^ | Alogliptin | 113 | 1 | 1 | 0 | 0 | 0 | 0 | 1 | 0 |
|  | Placebo | 115 | 1 | 0 | 0 | 1 | 0 | 0 | 0 | 0 |
| NCT00286442^9^ | Alogliptin | 210 | 0 | 0 | 0 | 0 | 0 | 0 | 0 | 0 |
|  | Placebo | 104 | 1 | 0 | 0 | 0 | 1 | 0 | 0 | 0 |
| NCT00286494^10^ | Alogliptin | 199 | 2 | 0 | 2 | 0 | 0 | 0 | 0 | 0 |
|  | Placebo | 97 | 0 | 0 | 0 | 0 | 0 | 0 | 0 | 0 |
| NCT00286429^11^ | Alogliptin | 129 | 1 | 0 | 0 | 0 | 1 | 0 | 0 | 1 |
|  | Placebo | 130 | 2 | 0 | 0 | 1 | 1 | 0 | 0 | 2 |
| NCT00395512^12^ | Alogliptin | 164 | 1 | 0 | 0 | 0 | 1 | 0 | NR | 0 |
|  | Thiazolidinedione | 163 | 0 | 0 | 0 | 0 | 0 | 0 | NR | 0 |
| NCT00996658^13^ | Linagliptin | 193 | 0 | 0 | 0 | 0 | 0 | 0 | 0 | 0 |
|  | Placebo | 89 | 1 | 1 | 0 | 0 | 0 | 0 | 1 | 0 |
| NCT01084005^14^ | Linagliptin | 162 | 2 | 0 | 0 | 1 | 1 | 0 | 0 | 1 |
|  | Placebo | 79 | 0 | 0 | 0 | 0 | 0 | 0 | 0 | 0 |
| NCT00800683^15^ | Linagliptin | 68 | 8 | 2 | 4 | 1 | 0 | 1 | 3 | 3 |
|  | Placebo | 65 | 9 | 3 | 3 | 2 | 1 | 0 | 3 | 3 |
| NCT00954447^16^ | Linagliptin | 631 | 9 | 5 | 0 | 0 | 3 | 1 | 5 | 11 |
|  | Placebo | 630 | 6 | 1 | 0 | 3 | 1 | 1 | 5 | 7 |
| NCT00622284^17^ | Linagliptin | 776 | 10 | 2 | 5 | 2 | 1 | 0 | 4 | 1 |
|  | Sulfonylurea | 775 | 23 | 2 | 9 | 10 | 1 | 1 | 4 | 12 |
| NCT00641043^18^ | Linagliptin | 259 | 1 | 0 | 0 | 0 | 0 | 1 | NR | 0 |
|  | Placebo | 130 | 0 | 0 | 0 | 0 | 0 | 0 | NR | 0 |
| NCT00798161^19^ | Linagliptin | 142 | 2 | 0 | 1 | 0 | 1 | 0 | 0 | 0 |
|  | Metformin | 291 | 1 | 1 | 0 | 0 | 0 | 0 | 1 | 1 |
|  | Placebo | 72 | 0 | 0 | 0 | 0 | 0 | 0 | 0 | 0 |
| NCT01422876^20, 21^ | Linagliptin | 267 | 2 | 0 | 1 | 1 | 0 | 0 | 0 | 0 |
|  | Empagliflozin | 551 | 2 | 0 | 1 | 0 | 0 | 1 | 3 | 0 |
| NCT01204294^22^ | Linagliptin | 228 | 2 | 0 | 0 | 2 | 0 | 0 | 0 | 0 |
|  | Metformin | 124 | 1 | 0 | 0 | 1 | 0 | 0 | 0 | 0 |
| NCT00602472^23^ | Linagliptin | 792 | 1 | 0 | 1 | 0 | 0 | 0 | NR | 21 |
|  | Placebo | 263 | 1 | 0 | 0 | 0 | 1 | 0 | NR | 13 |
| NCT00601250^24^ | Linagliptin | 524 | 1 | 0 | 1 | 0 | 0 | 0 | NR | 0 |
|  | Placebo | 177 | 0 | 0 | 0 | 0 | 0 | 0 | NR | 0 |
| NCT01006603^25^ | Saxagliptin | 360 | 4 | 1 | 2 | 0 | 1 | 0 | 1 | NR |
|  | Sulfonylurea | 360 | 4 | 0 | 3 | 1 | 0 | 0 | 1 | NR |
| NCT00757588^26, 27^ | Saxagliptin | 304 | 5 | 1 | 3 | 0 | 0 | 1 | 2 | 2 |
|  | Placebo | 151 | 2 | 0 | 1 | 1 | 0 | 0 | 0 | 3 |
| NCT00575588^28, 29^ | Saxagliptin | 428 | 3 | 1 | 1 | 1 | 0 | 0 | 4 | NR |
|  | Sulfonylurea | 430 | 5 | 2 | 0 | 1 | 2 | 0 | 2 | NR |
| NCT00121641^30, 31^ | Saxagliptin | 208 | 4 | 0 | 1 | 2 | 1 | 0 | 0 | 0 |
|  | Placebo | 95 | 3 | 1 | 1 | 0 | 1 | 0 | 1 | 0 |
| NCT01107886^32^ | Saxagliptin | 8280 | 710 | 269 | 265 | 157 | 97 | 0 | 420 | 177 |
|  | Placebo | 8212 | 690 | 260 | 278 | 141 | 81 | 0 | 378 | 140 |
| NCT00698932^33^ | Saxagliptin | 284 | 4 | 1 | 0 | 1 | 2 | 0 | 1 | 0 |
|  | Placebo | 284 | 0 | 0 | 0 | 0 | 0 | 0 | 0 | 0 |
| NCT00661362^34^ | Saxagliptin | 283 | 1 | 0 | 0 | 1 | 0 | 0 | 0 | 0 |
|  | Placebo | 287 | 1 | 0 | 0 | 1 | 0 | 0 | 0 | 0 |
| NCT00313313^35^ | Saxagliptin | 501 | 3 | 0 | 2 | 1 | 0 | 0 | 0 | 0 |
|  | Sulfonylurea | 267 | 6 | 1 | 3 | 2 | 0 | 0 | 1 | 0 |
| NCT00121667^36^ | Saxagliptin | 383 | 3 | 0 | 1 | 2 | 0 | 0 | 0 | 0 |
|  | Placebo | 179 | 5 | 1 | 3 | 0 | 1 | 0 | 1 | 0 |
| NCT00295633^37, 38^ | Saxagliptin | 381 | 4 | 0 | 1 | 3 | 0 | 0 | 2 | 0 |
|  | Placebo | 184 | 1 | 0 | 1 | 0 | 0 | 0 | 0 | 0 |
| NCT00327015^39, 40^ | Saxagliptin | 320 | 2 | 0 | 1 | 1 | 0 | 0 | 1 | 0 |
|  | Placebo | 328 | 5 | 4 | 1 | 0 | 0 | 0 | 5 | 0 |
| NCT00614939^41^ | Saxagliptin | 85 | 6 | 3 | 3 | 0 | 0 | 0 | 3 | 0 |
|  | Placebo | 85 | 5 | 3 | 1 | 1 | 0 | 0 | 4 | 2 |
| NCT01006590^42^ | Saxagliptin | 147 | 0 | 0 | 0 | 0 | 0 | 0 | 1 | 0 |
|  | Metformin | 139 | 0 | 0 | 0 | 0 | 0 | 0 | 1 | 0 |
| NCT01289990^43, 44^(1) | Placebo | 228 | 3 | 0 | 2 | 1 | 0 | 0 | 1 | 0 |
|  | Sitagliptin | 223 | 1 | 1 | 0 | 0 | 0 | 0 | 1 | 0 |
|  | Empagliflozin | 448 | 7 | 0 | 2 | 5 | 0 | 0 | 0 | 1 |
| NCT00790205^45^ | Sitagliptin | 7332 | 839 | 311 | 275 | 145 | 108 | 0 | 547 | 144 |
|  | Placebo | 7339 | 851 | 291 | 286 | 157 | 117 | 0 | 537 | 125 |
| NCT00722371^46^ | Sitagliptin | 172 | 3 | 1 | 2 | 0 | 0 | 0 | 1 | 2 |
|  | Thiazolidinedione | 515 | 2 | 0 | 0 | 2 | 0 | 0 | 1 | 1 |
| NCT01098539^47^ | Sitagliptin | 246 | 6 | 2 | 1 | 2 | 1 | 0 | 4 | 4 |
|  | Albiglutide | 249 | 5 | 2 | 1 | 2 | 0 | 0 | 4 | 1 |
| NCT00734474^48, 49^ | Sitagliptin | 315 | 4 | 1 | 2 | 1 | 0 | 0 | 2 | 0 |
|  | Dulaglutide | 606 | 5 | 1 | 3 | 1 | 0 | 0 | 1 | 0 |
| NCT01137812^50^ | Sitagliptin | 378 | 2 | 0 | 2 | 0 | 0 | 0 | 0 | 13 |
|  | Canagliflozin | 377 | 4 | 2 | 0 | 1 | 1 | 0 | 2 | 15 |
| NCT01177813^51^ | Sitagliptin | 223 | 0 | 0 | 0 | 0 | 0 | 0 | 0 | 0 |
|  | Empagliflozin | 448 | 3 | 0 | 1 | 1 | 1 | 0 | 0 | 0 |
|  | Placebo | 228 | 2 | 0 | 1 | 1 | 0 | 0 | 1 | 0 |
| NCT00509236^52^ | Sitagliptin | 64 | 1 | 0 | 1 | 0 | 0 | 0 | 4 | 0 |
|  | Sulfonylurea | 65 | 2 | 0 | 2 | 0 | 0 | 0 | 6 | 5 |
| NCT00509262^53^ | Sitagliptin | 210 | 7 | 2 | 3 | 2 | 0 | 0 | 3 | 3 |
|  | Sulfonylurea | 212 | 7 | 3 | 1 | 1 | 1 | 1 | 7 | 6 |
| NCT00350779^54^ | Sitagliptin | 170 | 3 | 0 | 2 | 1 | 0 | 0 | 0 | 0 |
|  | Placebo | 92 | 0 | 0 | 0 | 0 | 0 | 0 | 0 | 0 |
| NCT00885352^55^ | Sitagliptin | 157 | 0 | 0 | 0 | 0 | 0 | 0 | 0 | 0 |
|  | Placebo | 156 | 1 | 1 | 0 | 0 | 0 | 0 | 1 | 2 |
| NCT01046110^56^ | Sitagliptin | 226 | 3 | 0 | 0 | 1 | 1 | 1 | 0 | 0 |
|  | Insulin | 228 | 3 | 1 | 1 | 1 | 0 | 0 | 1 | 1 |
| NCT00751114^57^ | Sitagliptin | 264 | 1 | 0 | 1 | 0 | 0 | 0 | NR | 1 |
|  | Insulin | 237 | 2 | 0 | 0 | 0 | 2 | 0 | NR | 3 |
| NCT00701090^58^ | Sitagliptin | 516 | 2 | 0 | 2 | 0 | 0 | 0 | 0 | 1 |
|  | Sulfonylurea | 518 | 3 | 1 | 0 | 1 | 0 | 1 | 1 | 8 |
| NCT00700817^59, 60^ | Sitagliptin | 219 | 2 | 2 | 0 | 0 | 0 | 0 | 2 | 0 |
|  | Liraglutide | 446 | 2 | 0 | 2 | 0 | 0 | 0 | 1 | 1 |
| NCT00637273^61^ | Sitagliptin | 166 | 2 | 1 | 0 | 0 | 1 | 0 | 1 | 0 |
|  | Exenatide | 160 | 0 | 0 | 0 | 0 | 0 | 0 | 0 | 0 |
|  | Thiazolidinedione | 165 | 2 | 0 | 0 | 1 | 1 | 0 | 0 | 0 |
| NCT00094770^62, 63^ | Sitagliptin | 588 | 1 | 0 | 0 | 0 | 1 | 0 | 1 | 2 |
|  | Sulfonylurea | 584 | 5 | 2 | 3 | 0 | 0 | 0 | 8 | 18 |
| NCT00395343^64^ | Sitagliptin | 322 | 1 | 0 | 1 | 0 | 0 | 0 | 0 | 2 |
|  | Placebo | 319 | 3 | 0 | 2 | 0 | 1 | 0 | 0 | 1 |
| NCT00337610^65^ | Sitagliptin | 96 | 0 | 0 | 0 | 0 | 0 | 0 | 2 | NR |
|  | Placebo | 94 | 1 | 1 | 0 | 0 | 0 | 0 | 1 | NR |
| NCT00103857^66-68^（1） | Sitagliptin | 179 | 1 | 0 | 1 | 0 | 0 | 0 | 0 | 0 |
|  | Metformin | 364 | 2 | 0 | 1 | 0 | 1 | 0 | 0 | 2 |
| NCT00103857^66-68^（2） | Sitagliptin | 179 | 0 | 0 | 0 | 0 | 0 | 0 | 0 | 0 |
|  | Placebo | 176 | 1 | 1 | 0 | 0 | 0 | 0 | 1 | 0 |
| NCT01189890^69^ | Sitagliptin | 241 | 0 | 0 | 0 | 0 | 0 | 0 | 0 | 1 |
|  | Sulfonylurea | 236 | 1 | 0 | 0 | 0 | 1 | 0 | 0 | 3 |
| NCT00838903^70^ | Sitagliptin | 302 | 2 | 0 | 2 | 0 | 0 | 0 | NR | 0 |
|  | Placebo | 101 | 5 | 0 | 2 | 2 | 1 | 0 | NR | 0 |
|  | Albiglutide | 302 | 8 | 0 | 5 | 2 | 1 | 0 | NR | 0 |
|  | Sulfonylurea | 307 | 2 | 0 | 2 | 0 | 0 | 0 | NR | 0 |
| NCT01106677^71^ | Sitagliptin | 366 | 3 | 0 | 1 | 1 | 1 | 0 | 1 | 1 |
|  | Canagliflozin | 735 | 2 | 0 | 1 | 1 | 0 | 0 | 1 | 1 |
| NCT00676338^72^ | Sitagliptin | 163 | 0 | 0 | 0 | 0 | 0 | 0 | 0 | 0 |
|  | Exenatide | 248 | 0 | 0 | 0 | 0 | 0 | 0 | 0 | 0 |
|  | Metformin | 246 | 2 | 0 | 0 | 1 | 1 | 0 | 1 | 0 |
|  | Thiazolidinedione | 163 | 1 | 0 | 1 | 0 | 0 | 0 | 0 | 0 |
| NCT00397631^73^ | Sitagliptin | 261 | 0 | 0 | 0 | 0 | 0 | 0 | 0 | 0 |
|  | Placebo | 259 | 1 | 0 | 0 | 1 | 0 | 0 | 0 | 0 |
| NCT00449930^74^ | Sitagliptin | 528 | 0 | 0 | 0 | 0 | 0 | 0 | 1 | 2 |
|  | Metformin | 522 | 1 | 0 | 1 | 0 | 0 | 0 | 0 | 0 |
| NCT00106704^75^ | Sitagliptin | 222 | 2 | 0 | 0 | 2 | 0 | 0 | 1 | 0 |
|  | Placebo | 219 | 2 | 0 | 1 | 1 | 0 | 0 | 0 | 0 |
| NCT00881530^76^（1） | Sitagliptin | 56 | 4 | 0 | 0 | 3 | 1 | 0 | 0 | 0 |
|  | Empagliflozin | 332 | 4 | 0 | 1 | 1 | 2 | 0 | 0 | 0 |
| NCT01357252^77^ | Vildagliptin | 143 | 0 | 0 | 0 | 0 | 0 | 0 | 0 | 0 |
|  | Placebo | 135 | 1 | 0 | 0 | 1 | 0 | 0 | 0 | 0 |
| NCT01257451^78^ | Vildagliptin | 139 | 2 | 1 | 1 | 0 | 0 | 0 | 1 | 0 |
|  | Placebo | 139 | 1 | 0 | 1 | 0 | 0 | 0 | 1 | 0 |
| 2012Pan^79^ | Vildagliptin | 146 | 0 | 0 | 0 | 0 | 0 | 0 | 0 | 0 |
|  | Placebo | 144 | 1 | 0 | 0 | 1 | 0 | 0 | 0 | 0 |
| NCT00237237^80, 81^ | Vildagliptin | 295 | 2 | 0 | 0 | 1 | 0 | 1 | 0 | 0 |
|  | Thiazolidinedione | 280 | 2 | 0 | 0 | 1 | 0 | 1 | 0 | 0 |
| NCT00382096 and NCT00468039^82^ | Vildagliptin | 300 | 0 | 0 | 0 | 0 | 0 | 0 | 0 | 0 |
|  | Metformin | 294 | 2 | 0 | 2 | 0 | 0 | 0 | 0 | 1 |
| NCT00106340^83, 84^ | Vildagliptin | 1389/1533^*^ | 7 | 2 | 0 | 0 | 0 | 5 | 7 | 0 |
|  | Sulfonylurea | 1383/1546^*^ | 15 | 1 | 0 | 7 | 0 | 7 | 6 | 15 |
| NCT00383578^85^ | Vildagliptin | 167 | 1 | 1 | 0 | 0 | 0 | 0 | 1 | 0 |
|  | Metformin | 165 | 2 | 0 | 1 | 0 | 0 | 1 | 0 | 0 |
| NCT00099892^86^ | Vildagliptin | 185 | 1 | 0 | 0 | 1 | 0 | 0 | 0 | 0 |
|  | Placebo | 182 | 0 | 0 | 0 | 0 | 0 | 0 | 0 | 0 |
| NCT00099931^87^ | Vildagliptin | 144 | 0 | 0 | NR | NR | NR | NR | 1 | 0 |
|  | Placebo | 152 | 1 | 1 | NR | NR | NR | NR | 1 | 4 |
| NCT00099866^88^ | Vildagliptin | 519 | 0 | 0 | 0 | NR | NR | NR | 2 | 0 |
|  | Metformin | 252 | 3 | 1 | 2 | NR | NR | NR | 3 | 0 |
| NCT01649466^89^ | Vildagliptin | 82 | 0 | 0 | 0 | 0 | 0 | 0 | 0 | 0 |
|  | Insulin | 79 | 0 | 0 | 0 | 0 | 0 | 0 | 1 | 0 |
| 2013Kothny^90^ | Vildagliptin | 227 | NR | NR | NR | NR | NR | NR | 0 | 2 |
|  | Placebo | 221 | NR | NR | NR | NR | NR | NR | 1 | 2 |
| 2011Lukashevich^91^ | Vildagliptin | 287 | NR | NR | NR | NR | NR | NR | 4 | 4 |
|  | Placebo | 226 | NR | NR | NR | NR | NR | NR | 5 | 4 |
| NCT00102388^92^ | Vildagliptin | 546 | NR | NR | NR | NR | NR | NR | 6 | 0 |
|  | Sulfonylurea | 546 | NR | NR | NR | NR | NR | NR | 9 | 0 |
| NCT00099918^93^ | Vildagliptin | 515 | NR | NR | NR | NR | NR | NR | 1 | 0 |
|  | Thiazolidinedione | 267 | NR | NR | NR | NR | NR | NR | 0 | 0 |
| NCT00849017^94^ | Albiglutide | 200 | 1 | 0 | 0 | 1 | 0 | 0 | 3 | 0 |
|  | Placebo | 101 | 4 | 0 | 2 | 1 | 1 | 0 | 0 | 0 |
| NCT00839527^95^ | Albiglutide | 271 | 5 | 1 | 1 | 0 | 3 | 0 | 1 | 1 |
|  | Thiazolidinedione | 277 | 6 | 0 | 1 | 4 | 0 | 0 | 3 | 3 |
|  | Placebo | 115 | 4 | 0 | 1 | 2 | 1 | 0 | 1 | 0 |
| NCT00849056^96^ | Albiglutide | 150 | 3 | 0 | 2 | 0 | 1 | 0 | 0 | 2 |
|  | Placebo | 151 | 1 | 0 | 1 | 0 | 0 | 0 | 3 | 0 |
| NCT00976391^97^ | Albiglutide | 285 | 4 | 0 | 3 | 0 | 1 | 0 | NR | 0 |
|  | Insulin | 281 | 3 | 0 | 1 | 0 | 2 | 0 | NR | 2 |
| NCT00838916^98^ | Albiglutide | 504 | 11 | 0 | 6 | 2 | 2 | 1 | 3 | 2 |
|  | Insulin | 241 | 7 | 1 | 2 | 0 | 3 | 1 | 3 | 1 |
| NCT01128894^99^ | Albiglutide | 404 | 2 | 0 | 1 | 1 | 0 | 0 | 0 | 0 |
|  | Liraglutide | 408 | 4 | 1 | 2 | 1 | 0 | 0 | 1 | 0 |
| NCT01191268^100^ | Dulaglutide | 588 | 11 | 0 | 2 | 5 | 4 | 0 | 2 | 17 |
|  | Insulin | 296 | 12 | 2 | 6 | 2 | 2 | 0 | 3 | 15 |
| NCT01149421^101^ | Dulaglutide | 505 | 1 | 0 | 0 | 1 | 0 | 0 | 0 | NR |
|  | Placebo | 250 | 1 | 0 | 0 | 1 | 0 | 0 | 0 | NR |
| NCT01126580^102^ | Dulaglutide | 539 | 3 | 0 | 1 | 1 | 1 | 0 | 0 | 0 |
|  | Metformin | 268 | 0 | 0 | 0 | 0 | 0 | 0 | 0 | 0 |
| NCT01064687^103^ | Dulaglutide | 559 | 2 | 1 | 0 | 1 | 0 | 0 | 2 | 0 |
|  | Exenatide | 278 | 0 | 0 | 0 | 0 | 0 | 0 | 0 | 2 |
|  | Placebo | 141 | 0 | 0 | 0 | 0 | 0 | 0 | 0 | 0 |
| NCT00765817^104, 105^ | Exenatide | 137 | 0 | 0 | 0 | 0 | 0 | 0 | 0 | 0 |
|  | Placebo | 122 | 2 | 1 | 0 | 0 | 1 | 0 | 1 | 1 |
| NCT00359762^106, 107^ | Exenatide | 511 | 6 | 1 | 1 | 3 | 0 | 1 | 5 | 1 |
|  | Sulfonylurea | 508 | 5 | 0 | 4 | 0 | 0 | 1 | 5 | 0 |
| NCT01147627^108^ | Exenatide | 142 | 1 | 0 | 0 | 1 | 0 | 0 | NR | 0 |
|  | Insulin | 138 | 1 | 0 | 0 | 1 | 0 | 0 | NR | 0 |
|  | Thiazolidinedione | 136 | 1 | 0 | 0 | 1 | 0 | 0 | NR | 0 |
| NCT00960661^109^ | Exenatide | 315 | 4 | 1 | 0 | 1 | 2 | 0 | 1 | 2 |
|  | Insulin | 312 | 4 | 0 | 2 | 1 | 1 | 0 | 0 | 7 |
| NCT00641056^110-112^ | Exenatide | 233 | 2 | 0 | 0 | 1 | 0 | 1 | 1 | 0 |
|  | Insulin | 223 | 0 | 0 | 0 | 0 | 0 | 0 | 1 | 0 |
| NCT00082407^113^ | Exenatide | 253 | 2 | 0 | 2 | 0 | 0 | 0 | 2 | 0 |
|  | Insulin | 248 | 2 | 0 | 0 | 1 | 0 | 1 | 1 | 0 |
| NCT00935532^114^ | Exenatide | 215 | 2 | 1 | 1 | 0 | 0 | 0 | 1 | 0 |
|  | Insulin | 212 | 0 | 0 | 0 | 0 | 0 | 0 | 0 | 0 |
| NCT00434954^115^ | Exenatide | 247 | 1 | 0 | 1 | 0 | 0 | 0 | NR | 0 |
|  | Insulin | 233 | 0 | 0 | 0 | 0 | 0 | 0 | NR | 0 |
| NCT00603239^116^ | Exenatide | 111 | 1 | 0 | 0 | 1 | 0 | 0 | 0 | 0 |
|  | Placebo | 54 | 0 | 0 | 0 | 0 | 0 | 0 | 0 | 0 |
| NCT00097877^117^ | Exenatide | 124 | 0 | 0 | 0 | 0 | 0 | 0 | 0 | 0 |
|  | Insulin | 248 | 1 | 1 | 0 | 0 | 0 | 0 | 1 | 0 |
| 2009Davies^118^ | Exenatide | 118 | 1 | NR | 1 | NR | NR | NR | NR | 5 |
|  | Insulin | 116 | 0 | NR | 0 | NR | NR | NR | NR | 6 |
| 2004Buse^119^ | Exenatide | 254 | 1 | 0 | 1 | 0 | 0 | 0 | NR | 0 |
|  | Placebo | 123 | 1 | 0 | 1 | 0 | 0 | 0 | NR | 0 |
| NCT01029886^120^ | Exenatide | 461 | 2 | 0 | 1 | 1 | 0 | 0 | 0 | 0 |
|  | Liraglutide | 450 | 2 | 0 | 0 | 2 | 0 | 0 | 0 | 0 |
| NCT00518882^121^ | Liraglutide | 235 | 1 | 0 | 0 | 1 | 0 | 0 | NR | 0 |
|  | Exenatide | 232 | 2 | 0 | 1 | 1 | 0 | 0 | NR | 2 |
| NCT01179048^122^ | Placebo | 4672 | 818 | 277 | 304 | 163 | 124 | 0 | 447 | 153 |
|  | Liraglutide | 4668 | 730 | 181 | 275 | 152 | 122 | 0 | 381 | 114 |
| NCT01117350^123^ | Liraglutide | 489 | 2 | 0 | 0 | 2 | 0 | 0 | 0 | 2 |
|  | Insulin | 489 | 2 | 0 | 0 | 2 | 0 | 0 | 0 | 0 |
| NCT01336023^124^ | Liraglutide | 413 | 1 | 0 | 1 | 0 | 0 | 0 | 0 | 0 |
|  | Insulin | 413 | 1 | 0 | 1 | 0 | 0 | 0 | 0 | 1 |
| NCT01388361^125^ | Liraglutide | 87 | 0 | 0 | 0 | 0 | 0 | 0 | 0 | 0 |
|  | Insulin | 86 | 1 | 0 | 0 | 1 | 0 | 0 | 0 | 0 |
| NCT00294723^126-128^ | Liraglutide | 497 | 4 | 0 | 3 | 0 | 1 | 0 | 1 | 0 |
|  | Sulfonylurea | 248 | 2 | 0 | 2 | 0 | 0 | 0 | 1 | 0 |
| NCT00318461^129^ | Liraglutide | 484 | 4 | 0 | 2 | 2 | 0 | 0 | 0 | 0 |
|  | Sulfonylurea | 242 | 3 | 0 | 3 | 0 | 0 | 0 | 0 | 0 |
|  | Placebo | 121 | 0 | 0 | 0 | 0 | 0 | 0 | 0 | 0 |
| NCT01272232^130^ | Liraglutide | 210 | 3 | 1 | 2 | 0 | 0 | 0 | 1 | 2 |
|  | Placebo | 211 | 3 | 0 | 1 | 1 | 1 | 0 | 0 | 0 |
| NCT01147250^131^ | Lixisenatide | 3034 | 406 | 88 | 255 | 54 | 9 | 0 | 211 | 14 |
|  | Placebo | 3034 | 399 | 93 | 247 | 49 | 10 | 0 | 223 | 16 |
| NCT00713830^132^ | Lixisenatide | 574 | 1 | 1 | NR | NR | NR | NR | 1 | 1 |
|  | Placebo | 285 | 0 | 0 | NR | NR | NR | NR | 0 | 0 |
| NCT01169779^133^ | Lixisenatide | 196 | 2 | 0 | 1 | 1 | 0 | 0 | 0 | 0 |
|  | Placebo | 194 | 1 | 0 | 0 | 1 | 0 | 0 | 0 | 0 |
| NCT00763815^134^ | Lixisenatide | 323 | 0 | 0 | NR | NR | NR | NR | 0 | 0 |
|  | Placebo | 161 | 1 | 1 | NR | NR | NR | NR | 1 | 0 |
| NCT00715624^135^ | Lixisenatide | 328 | 1 | 1 | NR | NR | NR | NR | 1 | 4 |
|  | Placebo | 167 | 0 | 0 | NR | NR | NR | NR | 0 | 0 |
| NCT00975286^136^ | Lixisenatide | 223 | 0 | 0 | NR | NR | NR | NR | 0 | 1 |
|  | Placebo | 223 | 1 | 1 | NR | NR | NR | NR | 2 | 0 |
| NCT00866658^137^ | Lixisenatide | 154 | 2 | NR | NR | 2 | NR | NR | 0 | 0 |
|  | Placebo | 157 | 0 | NR | NR | 0 | NR | NR | 1 | 0 |
| NCT00763451^138^ | Exenatide | 322 | NR | NR | NR | NR | NR | NR | 3 | 0 |
|  | Placebo | 160 | NR | NR | NR | NR | NR | NR | 2 | 0 |
| NCT00707031^139^ | Lixisenatide | 318 | NR | NR | NR | NR | NR | NR | 1 | 0 |
|  | Exenatide | 316 | NR | NR | NR | NR | NR | NR | 1 | 0 |
| NCT01106651^140, 141^ | Canagliflozin | 477 | 12 | 2 | 5 | 3 | 2 | 0 | 2 | 11 |
|  | Placebo | 237 | 5 | 0 | 3 | 0 | 2 | 0 | 0 | 8 |
| NCT00968812^142, 143^ | Canagliflozin | 868 | 14 | 2 | 4 | 5 | 2 | 1 | 6 | 4 |
|  | Sulfonylurea | 482 | 8 | 1 | 3 | 2 | 2 | 0 | 2 | 16 |
| NCT01081834^144, 145^ | Canagliflozin | 392 | 0 | 0 | 0 | 0 | 0 | 0 | 0 | 0 |
|  | Placebo | 192 | 1 | 0 | 0 | 1 | 0 | 0 | 1 | 0 |
| NCT01064414^146, 147^ | Canagliflozin | 179 | 6 | 0 | 3 | 2 | 1 | 0 | 4 | 7 |
|  | Placebo | 90 | 4 | 0 | 3 | 0 | 0 | 1 | 2 | 1 |
| NCT01106625^148^ | Canagliflozin | 313 | 1 | 0 | 0 | 1 | 0 | 0 | 0 | 2 |
|  | Placebo | 156 | 1 | 0 | 1 | 0 | 0 | 0 | 0 | 1 |
| NCT01106690^149^ | Canagliflozin | 227 | 0 | 0 | 0 | 0 | 0 | 0 | 0 | 0 |
|  | Placebo | 115 | 1 | 0 | 0 | 0 | 0 | 1 | 0 | 0 |
| NCT01032629^150^ | Canagliflozin | 1382 | NR | NR | NR | NR | NR | NR | 13 | 69 |
|  | Placebo | 690 | NR | NR | NR | NR | NR | NR | 10 | 27 |
| NCT00528372^151, 152^ | Dapagliflozin | 278 | 2 | 0 | 1 | 0 | 0 | 1 | 1 | 0 |
|  | Placebo | 75 | 0 | 0 | 0 | 0 | 0 | 0 | 0 | 0 |
| NCT01031680^153^ | Dapagliflozin | 460 | 12 | 5 | 1 | 2 | 3 | 1 | 7 | 0 |
|  | Placebo | 462 | 12 | 2 | 2 | 1 | 7 | 0 | 2 | 0 |
| NCT00660907^154-156^ | Dapagliflozin | 406 | 2 | 0 | 1 | 0 | 0 | 1 | 2 | 0 |
|  | Sulfonylurea | 408 | 5 | 2 | 3 | 0 | 0 | 0 | 5 | 3 |
| NCT00984867^157^ | Dapagliflozin | 225 | 1 | 0 | 0 | 0 | 1 | 0 | 0 | 1 |
|  | Placebo | 226 | 0 | 0 | 0 | 0 | 0 | 0 | 1 | 1 |
| NCT00663260^158^ | Dapagliflozin | 168 | 4 | 4 | NR | NR | NR | NR | 5 | 2 |
|  | Placebo | 84 | 3 | 3 | NR | NR | NR | NR | 5 | 4 |
| NCT01042977^159^ | Dapagliflozin | 482 | 11 | 2 | 3 | 1 | 3 | 2 | 5 | 0 |
|  | Placebo | 483 | 7 | 1 | 1 | 3 | 2 | 0 | 4 | 0 |
| NCT00673231^160, 161^ | Dapagliflozin | 414 | 4 | 3 | 0 | 1 | 0 | 0 | 3 | 2 |
|  | Placebo | 197 | 1 | 0 | 0 | 1 | 0 | 0 | 0 | 1 |
| NCT00528879^162, 163^ | Dapagliflozin | 272 | 1 | 0 | 0 | 0 | 1 | 0 | 0 | 0 |
|  | Placebo | 137 | 5 | 0 | 3 | 1 | 1 | 0 | 1 | 0 |
| NCT00643851^164^ | Dapagliflozin | 203 | 1 | 1 | NR | NR | NR | NR | 1 | 0 |
|  | Metformin | 201 | 0 | 0 | NR | NR | NR | NR | 0 | 0 |
| NCT00859898^164^ | Dapagliflozin | 219 | 0 | 0 | NR | NR | NR | NR | 0 | 0 |
|  | Metformin | 208 | 1 | 1 | NR | NR | NR | NR | 1 | 0 |
| NCT00855166^165^ | Dapagliflozin | 91 | 1 | 0 | 1 | 0 | 0 | 0 | 1 | 0 |
|  | Placebo | 91 | 0 | 0 | 0 | 0 | 0 | 0 | 0 | 0 |
| NCT00683878^166^ | Dapagliflozin | 281 | 0 | 0 | 0 | 0 | 0 | 0 | 1 | 0 |
|  | Placebo | 139 | 0 | 0 | 0 | 0 | 0 | 0 | 0 | 0 |
| NCT00680745^167^ | Dapagliflozin | 296 | 1 | 1 | 0 | 0 | 0 | 0 | 1 | 0 |
|  | Placebo | 146 | 1 | 0 | 1 | 0 | 0 | 0 | 0 | 0 |
| NCT01131676^168^ | Placebo | 2333 | 333 | 137 | 121 | 60 | 66 | 0 | 194 | 36 |
|  | Empagliflozin | 4687 | 629 | 172 | 213 | 150 | 133 | 0 | 269 | 63 |
| NCT01210001^169^ | Empagliflozin | 333 | 3 | 3 | 0 | 0 | 0 | 0 | 4 | 1 |
|  | Placebo | 165 | 1 | 1 | 0 | 0 | 0 | 0 | 1 | 1 |
| NCT01368081^170^ | Empagliflozin | 273 | 1 | 0 | 1 | 0 | 0 | 0 | 0 | 0 |
|  | Metformin | 63 | 0 | 0 | 0 | 0 | 0 | 0 | 0 | 0 |
| NCT01164501^171^ | Empagliflozin | 321 | 5 | 1 | 0 | 4 | 0 | 0 | 1 | 5 |
|  | Placebo | 319 | 7 | 1 | 2 | 2 | 2 | 0 | 3 | 6 |
| NCT01289990^43, 44^（2） | Empagliflozin | 1202 | 13 | 1 | 5 | 3 | 3 | 1 | 4 | 1 |
|  | Placebo | 599 | 11 | 0 | 2 | 7 | 1 | 1 | 1 | 1 |
| NCT01167881^172^ | Empagliflozin | 765 | 11 | 1 | 1 | 7 | 2 | 0 | 5 | NR |
|  | Sulfonylurea | 780 | 16 | 0 | 7 | 7 | 2 | 0 | 5 | NR |
| NCT01306214^173^ | Empagliflozin | 375 | 2 | 0 | 1 | 1 | 0 | 0 | 1 | 2 |
|  | Placebo | 188 | 1 | 0 | 0 | 1 | 0 | 0 | 0 | 1 |
| NCT00881530^76^（2） | Empagliflozin | 215 | 2 | 0 | 1 | 1 | 0 | 0 | 0 | 0 |
|  | Metformin | 56 | 0 | 0 | 0 | 0 | 0 | 0 | 1 | 0 |
| NCT00521742^174^ | Glyburide | 152 | 2 | 2 | NR | NR | NR | NR | 2 | NR |
|  | Pioglitazone | 149 | 0 | 0 | NR | NR | NR | NR | 1 | NR |
| NCT00379769^175^ | Glyburide, Gliclazide, or Glimepiride | 2227 | 96 | 35 | 37 | NR | NR | NR | 80 | NR |
|  | Rosiglitazone | 2220 | 93 | 29 | 43 | NR | NR | NR | 70 | NR |
| NCT00225264^176^ | Glimepiride | 228 | 2 | 0 | 1 | 1 | 0 | 0 | 1 | NR |
|  | Pioglitazone | 230 | 0 | 0 | 0 | 0 | 0 | 0 | 0 | NR |
| 1998UKPDS^177^ | Glibenclamide | 619 | 149 | 69 | 46 | 34 | NR | 0 | 121 | 9 |
|  | Insulin | 911 | 211 | 102 | 79 | 30 | NR | 0 | 184 | 16 |
| 2008Hamann^178^ | Glibenclamide or Gliclazide | 301 | 1 | 1 | NR | NR | NR | NR | 2 | NR |
|  | Rosiglitazone | 294 | 2 | 2 | NR | NR | NR | NR | 2 | NR |
| NCT00513630^179^ | Glipizide | 148 | 35 | 17 | 6 | 15 | NR | 0 | 14 | 4 |
|  | Metformin | 156 | 22 | 4 | 5 | 10 | NR | 0 | 7 | 3 |
| NCT00484198^180^ | Pioglitazone | 751 | 5 | 1 | 1 | 2 | 1 | 0 | 2 | NR |
|  | Placebo | 137 | 0 | 0 | 0 | 0 | 0 | 0 | 0 | NR |
| NCT00116831^181^ | Glipizide | 339 | 17 | 3 | 6 | 1 | 7 | 0 | 7 | 3 |
|  | Rosiglitazone | 333 | 25 | 4 | 7 | 5 | 11 | 0 | 8 | 0 |
| NCT00494312^182^ | Glibenclamide | 1057 | 22 | 5 | 8 | 9 | NR | 0 | 6 | NR |
|  | Pioglitazone | 1063 | 17 | 1 | 6 | 10 | NR | 0 | 1 | NR |
| NCT00225277^183^ | Glibenclamide | 273 | 8 | 1 | 4 | 1 | 2 | 0 | 2 | NR |
|  | Pioglitazone | 270 | 9 | 3 | 2 | 0 | 4 | 0 | 3 | NR |
| 2006Bakris^184^ | Glyburide | 123 | 0 | 0 | NR | NR | NR | NR | 0 | NR |
|  | Rosiglitazone | 133 | 1 | 1 | NR | NR | NR | NR | 1 | NR |
| 2006Jain^185^ | Glyburide | 251 | 3 | 1 | 2 | NR | NR | 0 | 2 | NR |
|  | Pioglitazone | 251 | 2 | 0 | 2 | NR | NR | 0 | 0 | NR |
| NCT00279045^186^ | Glyburide | 1441 | 32 | 3 | 12 | 17 | NR | 0 | 31 | NR |
|  | Rosiglitazone | 1456 | 41 | 2 | 23 | 16 | NR | 0 | 34 | NR |
|  | Metformin | 1454 | 40 | 2 | 19 | 19 | NR | 0 | 31 | NR |
| 2006Rosenstock^187^ | Rosiglitazone | 159 | 1 | 0 | 1 | 0 | 0 | 0 | 0 | 0 |
|  | Metformin | 154 | 0 | 0 | 0 | 0 | 0 | 0 | 0 | 0 |
| 2005Charbonnel^188^ | Pioglitazone | 319 | 0 | 0 | 0 | 0 | 0 | 0 | 2 | 0 |
|  | Metformin | 320 | 1 | 0 | 1 | 0 | 0 | 0 | 0 | 0 |
| 2004Dailay^189^ | Rosiglitazone | 184 | NR | NR | NR | NR | NR | NR | 0 | 0 |
|  | Placebo | 184 | NR | NR | NR | NR | NR | NR | 1 | 0 |
| 2004Schernthaner^190^ | Pioglitazone | 597 | NR | NR | NR | NR | NR | NR | 3 | NR |
|  | Metformin | 597 | NR | NR | NR | NR | NR | NR | 2 | NR |
| 2000Fonseca^191^ | Rosiglitazone | 232 | 1 | 1 | NR | NR | NR | NR | 1 | 0 |
|  | Placebo | 116 | 0 | 0 | NR | NR | NR | NR | 0 | 0 |
| 2000Horton^192^ | Metformin | 178 | 1 | 1 | NR | NR | NR | NR | 1 | 0 |
|  | Placebo | 172 | 0 | 0 | NR | NR | NR | NR | 0 | 0 |
| 1998Johnston^193^ | Glyburide | 104 | 2 | 1 | NR | 1 | NR | NR | 2 | NR |
|  | Placebo | 101 | 0 | 0 | NR | 0 | NR | NR | 0 | NR |
| 1995DeFronzo^194^ | Glyburide | 209 | 0 | 0 | NR | NR | NR | NR | 0 | 0 |
|  | Metformin | 210 | 1 | 1 | NR | NR | NR | NR | 1 | 0 |

# Additional file 1: S7. Quality assessment of the included studies

| Study | Random sequence generation | Allocation concealment | Blinding of participants and personnel | Blinding of outcome assessment | Incomplete data | Selective reporting | Other source of bias |
| --- | --- | --- | --- | --- | --- | --- | --- |
| NCT00968708^1, 2^ | Unknown | Unknown | Low risk | Low risk | Low risk | Low risk | Low risk |
| NCT00856284^3^ | Unknown | Unknown | Low risk | Low risk | Low risk | Low risk | High risk |
| NCT01023581^4^ | Unknown | Unknown | Low risk | Low risk | Low risk | Low risk | Low risk |
| NCT00707993^5^ | Unknown | Unknown | Low risk | High risk | Low risk | Low risk | High risk |
| NCT00328627^6^ | Unknown | Unknown | Low risk | High risk | Low risk | Low risk | Low risk |
| NCT00432276^7^ | Unknown | Unknown | Low risk | High risk | Low risk | Low risk | High risk |
| NCT01318070^8^ | Low risk | Low risk | Low risk | High risk | Low risk | Low risk | Low risk |
| NCT00286442^9^ | Low risk | Low risk | Low risk | High risk | Low risk | Low risk | Low risk |
| NCT00286494^10^ | Low risk | Low risk | Low risk | High risk | Low risk | Low risk | Low risk |
| NCT00286429^11^ | Low risk | Low risk | Low risk | High risk | Low risk | Low risk | Low risk |
| NCT00395512^12^ | Unknown | Unknown | Low risk | High risk | Low risk | Low risk | High risk |
| NCT00996658^13^ | Low risk | Low risk | Low risk | Low risk | Low risk | Low risk | Low risk |
| NCT01084005^14^ | Low risk | Low risk | Low risk | Low risk | Low risk | Low risk | Low risk |
| NCT00800683^15^ | Unknown | Unknown | Low risk | Low risk | Low risk | Low risk | Low risk |
| NCT00954447^16^ | Low risk | Low risk | Low risk | Low risk | Low risk | Low risk | Low risk |
| NCT00622284^17^ | Low risk | Low risk | Low risk | Low risk | Low risk | Low risk | Low risk |
| NCT00641043^18^ | Low risk | Unknown | Low risk | High risk | Low risk | Low risk | Low risk |
| NCT00798161^19^ | Unknown | Unknown | Low risk | High risk | Low risk | Low risk | High risk |
| NCT01422876^20, 21^ | Unknown | Low risk | Low risk | High risk | Low risk | Low risk | Low risk |
| NCT01204294^22^ | Unknown | Unknown | High risk | High risk | Low risk | Low risk | High risk |
| NCT00602472^23^ | Unknown | Unknown | Low risk | High risk | Low risk | Low risk | Low risk |
| NCT00601250^24^ | Unknown | Unknown | Low risk | High risk | Low risk | Low risk | Low risk |
| NCT01006603^25^ | Unknown | Low risk | Low risk | High risk | Low risk | Low risk | Low risk |
| NCT00757588^26, 27^ | Unknown | Low risk | Low risk | High risk | Low risk | Low risk | Low risk |
| NCT00575588^28, 29^ | Unknown | Low risk | Low risk | High risk | Low risk | Low risk | High risk |
| NCT00121641^30, 31^ | Unknown | Unknown | Low risk | High risk | Low risk | Low risk | High risk |
| NCT01107886^32^ | Unknown | Low risk | Low risk | Low risk | Low risk | Low risk | Low risk |
| NCT00698932^33^ | Low risk | Unknown | Low risk | High risk | Low risk | Low risk | Low risk |
| NCT00661362^34^ | Low risk | Unknown | Low risk | High risk | Low risk | Low risk | Low risk |
| NCT00313313^35^ | Unknown | Low risk | Low risk | High risk | Low risk | Low risk | High risk |
| NCT00121667^36^ | Unknown | Low risk | Low risk | High risk | Low risk | Low risk | Low risk |
| NCT00295633^37, 38^ | Unknown | Low risk | Low risk | High risk | Low risk | Low risk | High risk |
| NCT00327015^39, 40^ | Unknown | Low risk | Low risk | High risk | Low risk | Low risk | High risk |
| NCT00614939^41^ | Unknown | Low risk | Low risk | High risk | Low risk | Low risk | Low risk |
| NCT01006590^42^ | Unknown | Unknown | Low risk | High risk | Low risk | Low risk | High risk |
| NCT01289990^43, 44^(1) | Low risk | Low risk | Low risk | High risk | Low risk | Low risk | Low risk |
| NCT00790205^45^ | Unknown | Low risk | Low risk | Low risk | Low risk | Low risk | High risk |
| NCT00722371^46^ | Unknown | Unknown | Low risk | High risk | Low risk | Low risk | High risk |
| NCT01098539^47^ | Unknown | Low risk | Low risk | Low risk | Low risk | Low risk | Low risk |
| NCT00734474^48, 49^ | Low risk | Low risk | Low risk | Low risk | Low risk | Low risk | High risk |
| NCT01137812^50^ | Low risk | Low risk | Low risk | High risk | Low risk | Low risk | High risk |
| NCT01177813^51^ | Low risk | Low risk | Low risk | High risk | Low risk | Low risk | Low risk |
| NCT00509236^52^ | Low risk | Unknown | Low risk | Low risk | Low risk | Low risk | Low risk |
| NCT00509262^53^ | Low risk | Unknown | Low risk | Low risk | Low risk | Low risk | Low risk |
| NCT00350779^54^ | Low risk | Unknown | Low risk | High risk | Low risk | Low risk | High risk |
| NCT00885352^55^ | Unknown | Unknown | Low risk | Low risk | Low risk | Low risk | High risk |
| NCT01046110^56^ | Unknown | Low risk | High risk | Low risk | Low risk | Low risk | Low risk |
| NCT00751114^57^ | Unknown | Low risk | High risk | High risk | Low risk | Low risk | Low risk |
| NCT00701090^58^ | Low risk | Unknown | Low risk | High risk | Low risk | Low risk | High risk |
| NCT00700817^59, 60^ | Low risk | Low risk | High risk | High risk | Low risk | Low risk | Low risk |
| NCT00637273^61^ | Low risk | Low risk | Low risk | High risk | Low risk | Low risk | Low risk |
| NCT00094770^62, 63^ | Low risk | Unknown | Low risk | High risk | Low risk | Low risk | Low risk |
| NCT00395343^64^ | Low risk | Unknown | Low risk | High risk | Low risk | Low risk | High risk |
| NCT00337610^65^ | Low risk | Unknown | Low risk | High risk | Low risk | Low risk | High risk |
| NCT00103857^66-68^（1） | Unknown | Unknown | Low risk | High risk | Low risk | Low risk | High risk |
| NCT00103857^66-68^（2） | Unknown | Unknown | Low risk | High risk | Low risk | Low risk | High risk |
| NCT01189890^69^ | Low risk | Low risk | Low risk | High risk | Low risk | Low risk | High risk |
| NCT00838903^70^ | Unknown | Unknown | Low risk | Low risk | Low risk | Low risk | Low risk |
| NCT01106677^71^ | Low risk | Unknown | Low risk | High risk | Low risk | Low risk | Low risk |
| NCT00676338^72^ | Low risk | Low risk | Low risk | High risk | Low risk | Low risk | Low risk |
| NCT00397631^73^ | Unknown | Unknown | Low risk | High risk | Low risk | Low risk | High risk |
| NCT00449930^74^ | Low risk | Unknown | Low risk | High risk | Low risk | Low risk | High risk |
| NCT00106704^75^ | Unknown | Low risk | Low risk | High risk | Low risk | Low risk | Low risk |
| NCT00881530^76^（1） | Low risk | Low risk | High risk | High risk | Low risk | Low risk | Low risk |
| NCT01357252^77^ | Unknown | Unknown | Low risk | High risk | Low risk | Low risk | Low risk |
| NCT01257451^78^ | Unknown | Low risk | Low risk | High risk | Low risk | Low risk | Low risk |
| 2012Pan^79^ | Unknown | Unknown | Low risk | High risk | Low risk | Low risk | High risk |
| NCT00237237^80, 81^ | Unknown | Low risk | Low risk | Low risk | Low risk | Low risk | High risk |
| NCT00382096 and NCT00468039^82^ | Unknown | Unknown | Low risk | High risk | Low risk | Low risk | High risk |
| NCT00106340^83, 84^ | Unknown | Unknown | Low risk | Low risk | Low risk | Low risk | High risk |
| NCT00383578^85^ | Unknown | Unknown | Low risk | Low risk | Low risk | Low risk | High risk |
| NCT00099892^86^ | Unknown | Unknown | Low risk | High risk | Low risk | Low risk | High risk |
| NCT00099931^87^ | Unknown | Unknown | Low risk | High risk | Low risk | High risk | High risk |
| NCT00099866^88^ | Unknown | Unknown | Low risk | High risk | Low risk | High risk | High risk |
| NCT01649466^89^ | Unknown | Unknown | High risk | High risk | Low risk | High risk | High risk |
| 2013Kothny^90^ | Unknown | Low risk | Low risk | High risk | Low risk | High risk | Low risk |
| 2011Lukashevich^91^ | Unknown | Unknown | Low risk | High risk | Low risk | High risk | High risk |
| NCT00102388^92^ | Unknown | Unknown | Low risk | High risk | Low risk | High risk | High risk |
| NCT00099918^93^ | Unknown | Unknown | Low risk | High risk | Low risk | High risk | High risk |
| NCT00849017^94^ | Unknown | Low risk | Low risk | Low risk | Low risk | Low risk | Low risk |
| NCT00839527^95^ | Unknown | Low risk | Low risk | Low risk | Low risk | Low risk | Low risk |
| NCT00849056^96^ | Unknown | Low risk | Low risk | Low risk | Low risk | Low risk | Low risk |
| NCT00976391^97^ | Unknown | Unknown | High risk | Low risk | Low risk | Low risk | Low risk |
| NCT00838916^98^ | Low risk | Low risk | High risk | Low risk | Low risk | Low risk | Low risk |
| NCT01128894^99^ | Unknown | Low risk | High risk | High risk | Low risk | Low risk | Low risk |
| NCT01191268^100^ | Low risk | Low risk | High risk | Low risk | Low risk | Low risk | Low risk |
| NCT01149421^101^ | Low risk | Unknown | Low risk | High risk | Low risk | Low risk | Low risk |
| NCT01126580^102^ | Unknown | Low risk | Low risk | High risk | Low risk | Low risk | Low risk |
| NCT01064687^103^ | Low risk | Low risk | High risk | High risk | Low risk | Low risk | Low risk |
| NCT00765817^104, 105^ | Low risk | Low risk | Low risk | High risk | Low risk | Low risk | Low risk |
| NCT00359762^106, 107^ | Low risk | Unknown | High risk | High risk | Low risk | Low risk | Low risk |
| NCT01147627^108^ | Unknown | Low risk | High risk | High risk | Low risk | Low risk | High risk |
| NCT00960661^109^ | Low risk | Unknown | High risk | High risk | Low risk | Low risk | Low risk |
| NCT00641056^110-112^ | Low risk | Low risk | High risk | High risk | Low risk | Low risk | Low risk |
| NCT00082407^113^ | Low risk | Low risk | High risk | High risk | Low risk | Low risk | Low risk |
| NCT00935532^114^ | Low risk | Low risk | High risk | High risk | Low risk | Low risk | Low risk |
| NCT00434954^115^ | Unknown | Unknown | High risk | High risk | Low risk | Low risk | Low risk |
| NCT00603239^116^ | Unknown | Unknown | Low risk | High risk | Low risk | Low risk | Low risk |
| NCT00097877^117^ | Unknown | Low risk | High risk | High risk | Low risk | Low risk | High risk |
| 2009Davies^118^ | Unknown | Unknown | High risk | High risk | Low risk | High risk | Low risk |
| 2004Buse^119^ | Unknown | Unknown | Low risk | High risk | Low risk | Low risk | Low risk |
| NCT01029886^120^ | Unknown | Low risk | High risk | High risk | Low risk | Low risk | Low risk |
| NCT00518882^121^ | Unknown | Low risk | High risk | High risk | Low risk | Low risk | Low risk |
| NCT01179048^122^ | Unknown | Low risk | Low risk | Low risk | Low risk | Low risk | Low risk |
| NCT01117350^123^ | Unknown | Low risk | High risk | High risk | Low risk | Low risk | Low risk |
| NCT01336023^124^ | Unknown | Low risk | High risk | Low risk | Low risk | Low risk | Low risk |
| NCT01388361^125^ | Unknown | Unknown | High risk | Low risk | Low risk | Low risk | High risk |
| NCT00294723^126-128^ | Unknown | Low risk | Low risk | High risk | Low risk | Low risk | Low risk |
| NCT00318461^129^ | Unknown | Low risk | Low risk | High risk | Low risk | Low risk | Low risk |
| NCT01272232^130^ | Unknown | Low risk | Low risk | Low risk | Low risk | Low risk | Low risk |
| NCT01147250^131^ | Unknown | Low risk | Low risk | Low risk | Low risk | Low risk | High risk |
| NCT00713830^132^ | Unknown | Unknown | Low risk | High risk | Low risk | High risk | Low risk |
| NCT01169779^133^ | Unknown | Low risk | Low risk | High risk | Low risk | Low risk | Low risk |
| NCT00763815^134^ | Unknown | Low risk | Low risk | High risk | Low risk | High risk | Low risk |
| NCT00715624^135^ | Unknown | Low risk | Low risk | Low risk | Low risk | High risk | Low risk |
| NCT00975286^136^ | Unknown | Low risk | Low risk | Low risk | Low risk | High risk | Low risk |
| NCT00866658^137^ | Unknown | Low risk | Low risk | High risk | Low risk | High risk | Low risk |
| NCT00763451^138^ | Unknown | Unknown | Low risk | High risk | Low risk | High risk | High risk |
| NCT00707031^139^ | Unknown | Low risk | High risk | High risk | Low risk | High risk | Low risk |
| NCT01106651^140, 141^ | Low risk | Low risk | Low risk | High risk | Low risk | Low risk | Low risk |
| NCT00968812^142, 143^ | Low risk | Low risk | Low risk | High risk | Low risk | Low risk | Low risk |
| NCT01081834^144, 145^ | Unknown | Unknown | Low risk | High risk | Low risk | Low risk | Low risk |
| NCT01064414^146, 147^ | Unknown | Low risk | Low risk | High risk | Low risk | Low risk | Low risk |
| NCT01106625^148^ | Low risk | Low risk | Low risk | High risk | Low risk | Low risk | Low risk |
| NCT01106690^149^ | Unknown | Low risk | Low risk | High risk | Low risk | Low risk | Low risk |
| NCT01032629^150^ | Low risk | Low risk | Low risk | Low risk | Low risk | High risk | Low risk |
| NCT00528372^151, 152^ | Unknown | Unknown | Low risk | High risk | Low risk | Low risk | High risk |
| NCT01031680^153^ | Unknown | Unknown | Low risk | High risk | Low risk | Low risk | Low risk |
| NCT00660907^154-156^ | Low risk | Low risk | Low risk | High risk | Low risk | Low risk | High risk |
| NCT00984867^157^ | Unknown | Unknown | Low risk | High risk | Low risk | Low risk | Low risk |
| NCT00663260^158^ | Unknown | Unknown | Low risk | High risk | Low risk | High risk | Low risk |
| NCT01042977^159^ | Unknown | Low risk | Low risk | High risk | Low risk | Low risk | Low risk |
| NCT00673231^160, 161^ | Low risk | Unknown | Low risk | High risk | Low risk | Low risk | Low risk |
| NCT00528879^162, 163^ | Low risk | Low risk | Low risk | High risk | Low risk | Low risk | Low risk |
| NCT00643851^164^ | Unknown | Low risk | Low risk | High risk | Low risk | High risk | Low risk |
| NCT00859898^164^ | Unknown | Low risk | Low risk | High risk | Low risk | High risk | Low risk |
| NCT00855166^165^ | Low risk | Low risk | Low risk | High risk | Low risk | Low risk | Low risk |
| NCT00683878^166^ | Unknown | Unknown | Low risk | High risk | Low risk | Low risk | Low risk |
| NCT00680745^167^ | Low risk | Unknown | Low risk | High risk | Low risk | Low risk | High risk |
| NCT01131676^168^ | Low risk | Low risk | Low risk | Low risk | Low risk | Low risk | Low risk |
| NCT01210001^169^ | Low risk | Low risk | Low risk | High risk | Low risk | Low risk | Low risk |
| NCT01368081^170^ | Low risk | Low risk | High risk | High risk | Low risk | Low risk | Low risk |
| NCT01164501^171^ | Low risk | Low risk | Low risk | High risk | Low risk | Low risk | Low risk |
| NCT01289990^43, 44^（2） | Unknown | Low risk | Low risk | High risk | Low risk | Low risk | Low risk |
| NCT01167881^172^ | Low risk | Low risk | Low risk | High risk | Low risk | Low risk | Low risk |
| NCT01306214^173^ | Unknown | Low risk | Low risk | High risk | Low risk | Low risk | Low risk |
| NCT00881530^76^（2） | Low risk | Low risk | High risk | High risk | Low risk | Low risk | Low risk |
| NCT00521742^174^ | Unknown | Unknown | Low risk | Low risk | Low risk | High risk | High risk |
| NCT00379769^175^ | Low risk | Low risk | High risk | High risk | Low risk | Low risk | Low risk |
| NCT00225264^176^ | Unknown | Unknown | Low risk | Low risk | Low risk | Low risk | High risk |
| 1998UKPDS^177^ | Low risk | Low risk | High risk | High risk | Low risk | Low risk | Low risk |
| 2008Hamann^178^ | Low risk | Low risk | Low risk | High risk | Low risk | High risk | Low risk |
| NCT00513630^179^ | Low risk | Low risk | Low risk | High risk | Low risk | Low risk | High risk |
| NCT00484198^180^ | Unknown | Unknown | Low risk | Low risk | Low risk | Low risk | High risk |
| NCT00116831^181^ | Unknown | Unknown | Low risk | Low risk | Low risk | Low risk | Low risk |
| NCT00494312^182^ | Unknown | Low risk | Low risk | High risk | Low risk | Low risk | Low risk |
| NCT00225277^183^ | Low risk | Low risk | Low risk | Low risk | Low risk | Low risk | Low risk |
| 2006Bakris^184^ | Unknown | Unknown | Low risk | High risk | Low risk | High risk | Low risk |
| 2006Jain^185^ | Unknown | Unknown | Low risk | High risk | Low risk | High risk | Low risk |
| NCT00279045^186^ | Low risk | Low risk | Low risk | Low risk | Low risk | High risk | Low risk |
| 2006Rosenstock^187^ | Unknown | Unknown | Low risk | High risk | Low risk | Low risk | Low risk |
| 2005Charbonnel^188^ | Unknown | Low risk | Low risk | High risk | Low risk | High risk | High risk |
| 2004Dailay^189^ | Unknown | Unknown | Low risk | High risk | Low risk | High risk | High risk |
| 2004Schernthaner^190^ | Low risk | Low risk | Low risk | High risk | Low risk | High risk | High risk |
| 2000Fonseca^191^ | Low risk | Unknown | Low risk | High risk | Low risk | High risk | High risk |
| 2000Horton^192^ | Low risk | Unknown | Low risk | High risk | Low risk | High risk | High risk |
| 1998Johnston^193^ | Unknown | Unknown | Low risk | High risk | Low risk | High risk | High risk |
| 1995DeFronzo^194^ | Unknown | Unknown | Low risk | High risk | Low risk | High risk | High risk |

# Additional file 1: S8. Comparison-adjusted funnel plot for the network


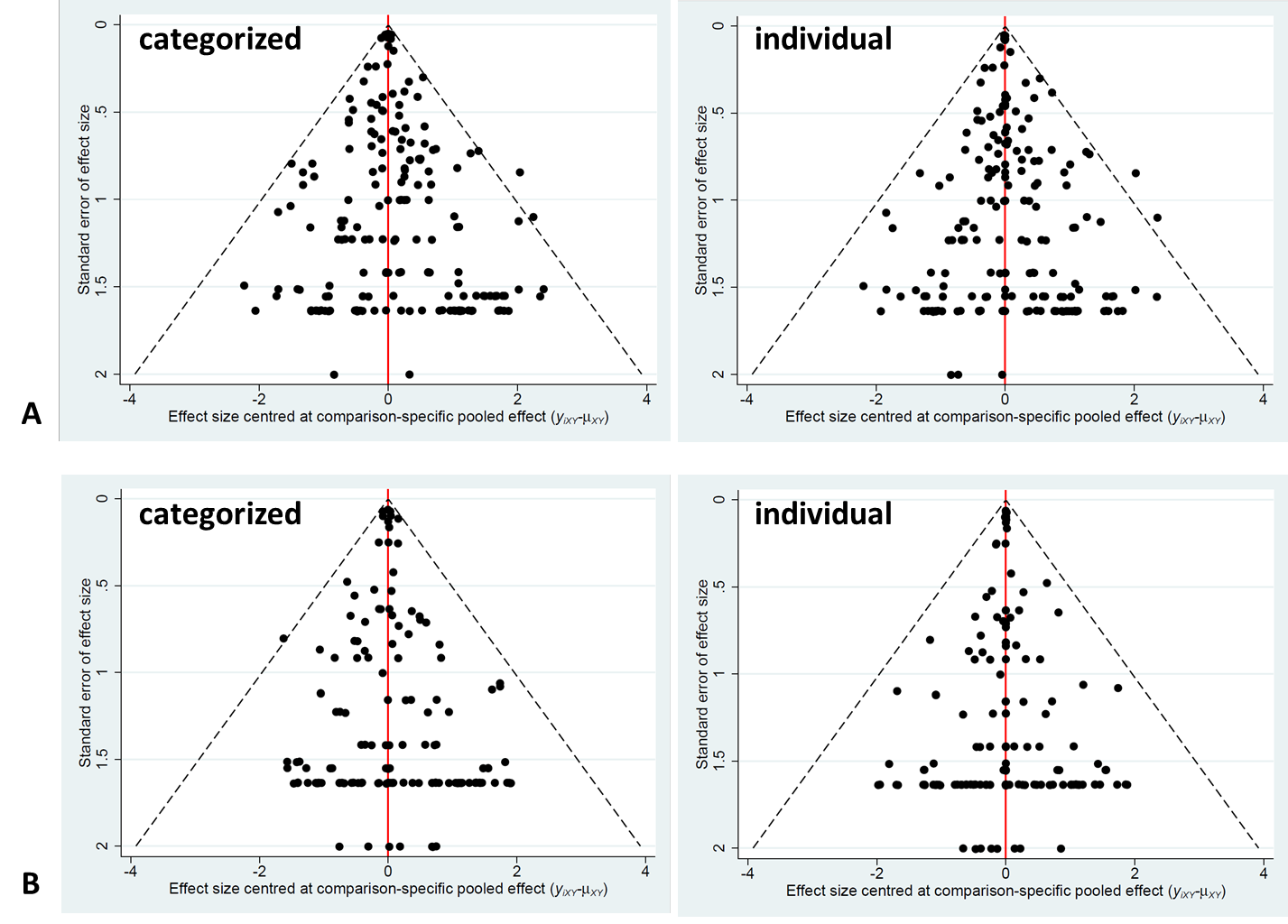


Figure: Comparison-adjusted funnel plot for the network for the outcome of MACE (A) and all-cause mortality (B) are shown.

Each observation is the difference between the study estimate and its direct meta-analysis mean effect. Studies on the right hand side ‘over-estimate’ the effect of active treatment. The green line is the regression line for small-study effect.

# Supplemental 9. Ranking ordered according to surface under the cumulative ranking values of outcomes


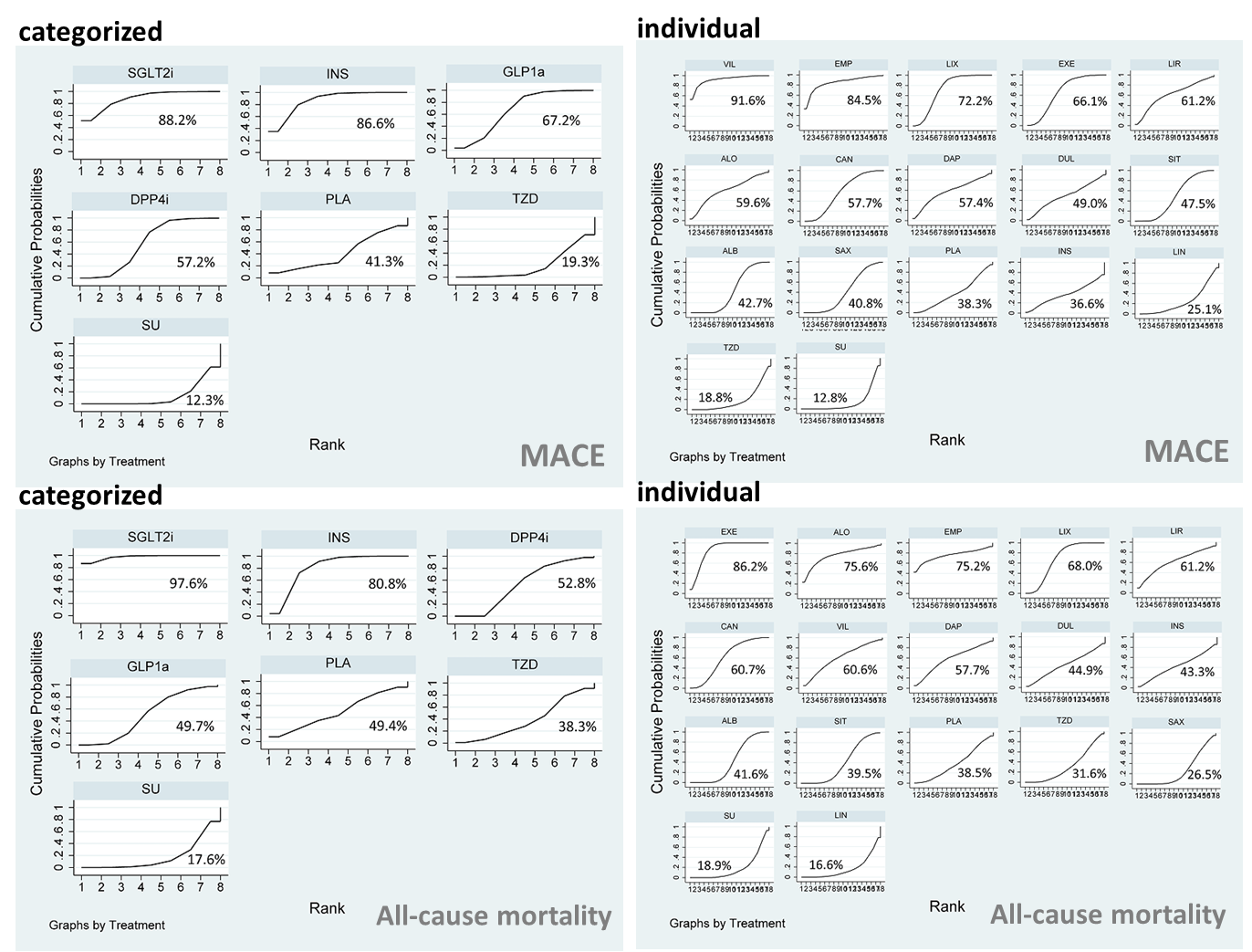


# Additional file 1: S10. Consistency analysis of direct verse indirect comparisons for outcomes

| Table A. Consistency analysis Direct vs. Indirect for MACE | | | | | | | | | |
| --- | --- | --- | --- | --- | --- | --- | --- | --- | --- |
| **Side**  **.** | | **Events/Total** | **Direct** | | **Indirect** | | **Difference** | | |
|  |  |  | **Coef** | **Std.Err.** | **Coef.** | **Std.Err..** | **Coef.** | **Std.Err** | **P>\|z\|** |
| Placebo | Saxagliptin | 1,451/20,333 | 0.0149 | 0.0550 | -0.377 | 0.447 | 0.392 | 0.451 | 0.384 |
| Placebo | Sitagliptin | 1,718/18,698 | -0.0305 | 0.0513 | 0.184 | 0.239 | -0.215 | 0.244 | 0.379 |
| Placebo | Sulfonylurea | 12,/976 | -1.050 | 0.664 | 0.377 | 0.161 | -1.427 | 0.684 | **0.0370** |
| Placebo | Thiazolidinedione | 18/2,145 | -0.602 | 0.568 | -0.140 | 0.362 | -0.462 | 0.682 | 0.498 |
| Placebo | Vildagliptin | 7/1,509 | -0.174 | 0.682 | -0.703 | 0.399 | 0.529 | 0.790 | 0.503 |
| Albiglutide | Placebo | 31/1,391 | 0.528 | 0.387 | -0.0761 | 0.338 | 0.605 | 0.522 | 0.247 |
| Albiglutide | Insulin | 25/1,311 | 0.128 | 0.413 | 0.250 | 0.429 | -0.122 | 0.595 | 0.838 |
| Albiglutide | Liraglutide | 6/812 | 0.688 | 0.869 | -0.00482 | 0.266 | 0.693 | 0.909 | 0.446 |
| Albiglutide | Sitagliptin | 21/1,099 | -0.367 | 0.468 | 0.425 | 0.317 | -0.791 | 0.586 | 0.177 |
| Albiglutide | Sulfonylurea | 10/609 | -0.995 | 0.726 | 0.810 | 0.322 | -1.805 | 0.814 | **0.0270** |
| Albiglutide | Thiazolidinedione | 11/548 | 0.148 | 0.611 | -0.214 | 0.443 | 0.362 | 0.756 | 0.632 |
| Alogliptin | Placebo | 713/6,953 | 0.0622 | 0.0802 | 0.145 | 0.395 | -0.0825 | 0.403 | 0.838 |
| Alogliptin | Metformin | 1/332 | -1.823 | 1.587 | 0.125 | 0.387 | -1.948 | 1.623 | 0.230 |
| Alogliptin | Sulfonylurea | 23/2,188 | 0.456 | 0.430 | 0.344 | 0.184 | 0.112 | 0.468 | 0.810 |
| Alogliptin | Thiazolidinedione | 9/1,647 | -0.0350 | 0.633 | -0.268 | 0.356 | 0.233 | 0.733 | 0.751 |
| Canagliflozin | Placebo | 31/2,378 | 0.250 | 0.378 | -0.133 | 0.380 | 0.383 | 0.536 | 0.475 |
| Canagliflozin | Sitagliptin | 11/1,856 | 0.157 | 0.630 | 0.0111 | 0.299 | 0.146 | 0.698 | 0.834 |
| Canagliflozin | Sulfonylurea | 22/1,350 | 0.0291 | 0.447 | 0.569 | 0.362 | -0.540 | 0.575 | 0.348 |
| Dapagliflozin | Placebo | 66/4,587 | -0.0314 | 0.258 | 0.459 | 0.702 | -0.491 | 0.748 | 0.512 |
| Dapagliflozin | Metformin | 2/831 | 0.0307 | 1.157 | -0.0184 | 0.462 | 0.0491 | 1.246 | 0.969 |
| Dapagliflozin | Sulfonylurea | 7/814 | 0.919 | 0.840 | 0.248 | 0.297 | 0.671 | 0.891 | 0.452 |
| Dulaglutide | Placebo | 4/1,455 | 0.0869 | 1.021 | 0.582 | 0.398 | -0.495 | 1.103 | 0.654 |
| Dulaglutide | Exenatide | 2/837 | -0.932 | 1.472 | 0.682 | 0.449 | -1.614 | 1.543 | 0.296 |
| Dulaglutide | Insulin | 23/884 | 0.796 | 0.424 | -0.0272 | 0.587 | 0.823 | 0.724 | 0.255 |
| Dulaglutide | Metformin | 3/807 | -1.254 | 1.514 | 0.690 | 0.534 | -1.944 | 1.605 | 0.226 |
| Dulaglutide | Sitagliptin | 9/921 | 0.436 | 0.674 | 0.517 | 0.439 | -0.0813 | 0.805 | 0.920 |
| Empagliflozin | Placebo | 1,020/11,874 | 0.0849 | 0.0705 | 0.425 | 0.332 | -0.340 | 0.339 | 0.316 |
| Empagliflozin | Linagliptin | 4/818 | 0.728 | 1.003 | -0.0450 | 0.255 | 0.773 | 1.035 | 0.455 |
| Empagliflozin | Metformin | 3/607 | 0.0225 | 1.129 | 0.0659 | 0.399 | -0.0434 | 1.197 | 0.971 |
| Empagliflozin | Sitagliptin | 19/1,730 | 0.556 | 0.518 | 0.0645 | 0.0860 | 0.491 | 0.526 | 0.350 |
| Empagliflozin | Sulfonylurea | 27/1,545 | 0.362 | 0.395 | 0.403 | 0.182 | -0.0414 | 0.435 | 0.924 |
| Exenatide | Placebo | 5/1,220 | 0.723 | 0.808 | -0.157 | 0.338 | 0.880 | 0.876 | 0.315 |
| Exenatide | Insulin | 21/3,377 | -0.353 | 0.430 | 0.394 | 0.488 | -0.747 | 0.650 | 0.251 |
| Exenatide | Liraglutide | 7/1,378 | -0.269 | 0.777 | -0.137 | 0.343 | -0.132 | 0.849 | 0.876 |
| Exenatide | Metformin | 2/494 | 1.215 | 1.372 | -0.253 | 0.507 | 1.468 | 1.479 | 0.321 |
| Exenatide | Sitagliptin | 2/737 | 0.642 | 1.124 | -0.0935 | 0.322 | 0.735 | 1.152 | 0.523 |
| Exenatide | Sulfonylurea | 11/1,019 | -0.178 | 0.609 | 0.453 | 0.389 | -0.631 | 0.723 | 0.382 |
| Exenatide | Thiazolidinedione | 8/1,014 | 0.772 | 0.824 | -0.659 | 0.474 | 1.431 | 0.953 | 0.133 |
| Insulin | Liraglutide | 7/1,977 | -0.223 | 0.732 | -0.117 | 0.301 | -0.106 | 0.791 | 0.893 |
| Insulin | Sitagliptin | 9/955 | -0.243 | 0.683 | 0.0223 | 0.302 | -0.265 | 0.747 | 0.722 |
| Insulin | Thiazolidinedione | 2/274 | 0.0147 | 1.419 | -0.299 | 0.400 | 0.314 | 1.475 | 0.832 |
| Linagliptin | Placebo | 41/4,276 | -0.0842 | 0.320 | 0.337 | 0.367 | -0.421 | 0.489 | 0.389 |
| Linagliptin | Metformin | 6/785 | -0.771 | 0.792 | 0.365 | 0.482 | -1.136 | 0.925 | 0.219 |
| Linagliptin | Sulfonylurea | 33/1,551 | 0.851 | 0.382 | 0.0459 | 0.333 | 0.805 | 0.507 | 0.112 |
| Liraglutide | Placebo | 1,558/10,366 | 0.134 | 0.0555 | 0.0803 | 0.395 | 0.0534 | 0.400 | 0.894 |
| Liraglutide | Sitagliptin | 4/665 | 0.716 | 1.003 | 0.108 | 0.0743 | 0.608 | 1.006 | 0.546 |
| Liraglutide | Sulfonylurea | 13/1,471 | 0.257 | 0.553 | 0.445 | 0.171 | -0.188 | 0.579 | 0.746 |
| Lixisenatide | Placebo | 814/9,053 | . | . | . | . | . | . | . |
| Metformin | Placebo | 2/1,039 | 0.939 | 1.209 | -0.0739 | 0.399 | 1.013 | 1.294 | 0.434 |
| Metformin | Sitagliptin | 6/2,002 | -0.627 | 0.798 | 0.225 | 0.437 | -0.852 | 0.933 | 0.361 |
| Metformin | Thiazolidinedione | 85/3,632 | -0.264 | 1.031 | -0.231 | 0.505 | -0.0324 | 1.151 | 0.978 |
| Metformin | Vildagliptin | 8/1,697 | -1.533 | 0.813 | -0.0794 | 0.544 | -1.454 | 0.978 | 0.137 |
| Saxagliptin | Sulfonylurea | 25/2,346 | 0.619 | 0.415 | 0.226 | 0.177 | 0.392 | 0.451 | 0.384 |
| Sitagliptin | Sulfonylurea | 33/3,843 | 0.357 | 0.367 | 0.308 | 0.178 | 0.0494 | 0.408 | 0.904 |
| Sitagliptin | Thiazolidinedione | 10/1,344 | -0.485 | 0.592 | -0.168 | 0.358 | -0.316 | 0.699 | 0.651 |
| Sulfonylurea | Vildagliptin | 22/2,772 | -0.772 | 0.459 | -0.976 | 0.509 | 0.204 | 0.685 | 0.766 |
| Thiazolidinedione | Vildagliptin | 4/575 | -0.0526 | 1.003 | -0.345 | 0.474 | 0.293 | 1.110 | 0.792 |

| Table B. Consistency analysis Direct vs. Indirect for all-cause mortality | | | | | | | | | |
| --- | --- | --- | --- | --- | --- | --- | --- | --- | --- |
| **Side** | | **Events/Total** | **Direct** | | **Indirect** | | **Difference** | | |
|  |  |  | **Coef.** | **Std.Err.** | **Coef.** | **Std.Err.** | **Coef.** | **Std.Err.** | **P>\|z\|** |
| Placebo | Saxagliptin | 818/19,763 | 0.0891 | 0.0718 | 0.336 | 0.648 | -0.247 | 0.652 | 0.705 |
| Placebo | Sitagliptin | 1,093/16,782 | 0.0188 | 0.0628 | -0.310 | 0.344 | 0.329 | 0.350 | 0.347 |
| Placebo | Thiazolidinedione | 9/2,507 | 0.294 | 0.922 | -0.632 | 0.666 | 0.925 | 1.180 | 0.433 |
| Placebo | Vildagliptin | 14/1,535 | -0.401 | 0.531 | -0.0103 | 0.391 | -0.390 | 0.659 | 0.554 |
| Albiglutide | Placebo | 8/988 | 0.531 | 0.855 | 0.492 | 0.535 | 0.0386 | 1.009 | 0.970 |
| Albiglutide | Insulin | 6/745 | 0.744 | 0.820 | 0.441 | 0.777 | 0.303 | 1.130 | 0.788 |
| Albiglutide | Liraglutide | 1/812 | 1.091 | 1.635 | 0.262 | 0.478 | 0.829 | 1.703 | 0.626 |
| Albiglutide | Sitagliptin | 8/495 | 0.0123 | 0.713 | 0.851 | 0.588 | -0.838 | 0.924 | 0.364 |
| Albiglutide | Thiazolidinedione | 4/548 | 1.122 | 1.125 | -0.255 | 0.783 | 1.377 | 1.382 | 0.319 |
| Alogliptin | Placebo | 327/5,866 | 0.136 | 0.113 | 0.188 | 0.692 | -0.0522 | 0.702 | 0.941 |
| Alogliptin | Sulfonylurea | 8/1,747 | 0.523 | 0.732 | 0.295 | 0.257 | 0.228 | 0.776 | 0.768 |
| Alogliptin | Thiazolidinedione | 2/1,320 | -0.503 | 1.138 | -0.0686 | 0.604 | -0.435 | 1.305 | 0.739 |
| Canagliflozin | Placebo | 32/3,639 | 0.356 | 0.361 | -0.533 | 0.660 | 0.889 | 0.752 | 0.237 |
| Canagliflozin | Sitagliptin | 4/1,856 | -0.354 | 1.046 | 0.213 | 0.337 | -0.567 | 1.099 | 0.606 |
| Canagliflozin | Sulfonylurea | 8/1,450 | -0.403 | 0.818 | 0.519 | 0.410 | -0.922 | 0.915 | 0.314 |
| Dapagliflozin | Placebo | 37/5,007 | -0.0924 | 0.337 | 0.417 | 0.719 | -0.509 | 0.794 | 0.521 |
| Dapagliflozin | Metformin | 2/831 | 0.0307 | 1.157 | 0.427 | 0.577 | -0.396 | 1.293 | 0.759 |
| Dapagliflozin | Sulfonylurea | 7/814 | 0.919 | 0.840 | 0.0191 | 0.395 | 0.900 | 0.928 | 0.332 |
| Dulaglutide | Placebo | 2/700 | -0.629 | 1.478 | 1.067 | 0.790 | -1.695 | 1.720 | 0.324 |
| Dulaglutide | Exenatide | 2/837 | -1.022 | 1.476 | 1.169 | 0.826 | -2.191 | 1.732 | 0.206 |
| Dulaglutide | Insulin | 5/884 | 1.099 | 0.916 | 0.335 | 0.993 | 0.764 | 1.351 | 0.572 |
| Dulaglutide | Sitagliptin | 3/921 | 1.352 | 1.227 | 0.377 | 0.812 | 0.975 | 1.471 | 0.508 |
| Empagliflozin | Placebo | 480/11,894 | 0.394 | 0.0962 | -0.0920 | 0.560 | 0.486 | 0.567 | 0.392 |
| Empagliflozin | Linagliptin | 3/718 | -1.429 | 1.514 | 0.316 | 0.404 | -1.745 | 1.567 | 0.265 |
| Empagliflozin | Metformin | 1/271 | 2.455 | 1.640 | 0.582 | 0.479 | 1.873 | 1.708 | 0.273 |
| Empagliflozin | Sitagliptin | 1/1,342 | 1.176 | 1.216 | 0.382 | 0.113 | 0.794 | 1.221 | 0.516 |
| Empagliflozin | Sulfonylurea | 10/1,545 | -0.0195 | 0.635 | 0.656 | 0.253 | -0.675 | 0.683 | 0.323 |
| Exenatide | Placebo | 6/1,160 | 0.540 | 0.744 | -0.151 | 0.468 | 0.691 | 0.879 | 0.432 |
| Exenatide | Insulin | 8/2,383 | -0.477 | 0.662 | 0.907 | 0.752 | -1.384 | 1.002 | 0.167 |
| Exenatide | Lixisenatide | 2/634 | -0.00633 | 1.416 | -0.0285 | 0.424 | 0.0222 | 1.479 | 0.988 |
| Exenatide | Metformin | 1/494 | 1.056 | 1.560 | 0.285 | 0.626 | 0.771 | 1.689 | 0.648 |
| Exenatide | Sitagliptin | 1/737 | 0.859 | 1.246 | -0.0318 | 0.418 | 0.891 | 1.306 | 0.495 |
| Exenatide | Sulfonylurea | 10/1,019 | 0.00595 | 0.636 | 0.385 | 0.533 | -0.379 | 0.829 | 0.648 |
| Exenatide | Thiazolidinedione | 0/736 | 0.194 | 1.416 | -0.371 | 0.705 | 0.565 | 1.582 | 0.721 |
| Insulin | Sitagliptin | 1/454 | -1.094 | 1.636 | 0.0348 | 0.532 | -1.129 | 1.720 | 0.512 |
| Insulin | Vildagliptin | 1/161 | -1.148 | 1.641 | -0.104 | 0.605 | -1.044 | 1.749 | 0.550 |
| Linagliptin | Placebo | 17/1,890 | 0.207 | 0.470 | 0.127 | 0.652 | 0.0803 | 0.803 | 0.920 |
| Linagliptin | Metformin | 1/433 | 0.243 | 1.586 | 0.573 | 0.614 | -0.329 | 1.706 | 0.847 |
| Linagliptin | Sulfonylurea | 8/1,551 | 0.00130 | 0.709 | 0.540 | 0.497 | -0.539 | 0.866 | 0.534 |
| Liraglutide | Placebo | 829/9,761 | 0.172 | 0.0729 | 0.644 | 0.819 | -0.472 | 0.822 | 0.566 |
| Liraglutide | Sitagliptin | 3/665 | 1.411 | 1.228 | 0.176 | 0.0954 | 1.235 | 1.231 | 0.316 |
| Liraglutide | Sulfonylurea | 2/745 | 0.697 | 1.416 | 0.349 | 0.235 | 0.348 | 1.436 | 0.808 |
| Lixisenatide | Placebo | 440/8,663 | 0.0725 | 0.0987 | 0.0503 | 1.475 | 0.0222 | 1.479 | 0.988 |
| Metformin | Placebo | 2/732 | 0.254 | 1.590 | -0.409 | 0.476 | 0.663 | 1.675 | 0.692 |
| Metformin | Saxagliptin | 2/286 | -0.0564 | 1.419 | -0.280 | 0.481 | 0.224 | 1.499 | 0.881 |
| Metformin | Sitagliptin | 3/2,002 | -0.0443 | 0.929 | -0.441 | 0.530 | 0.397 | 1.086 | 0.715 |
| Metformin | Thiazolidinedione | 71/4,513 | -0.899 | 1.560 | -0.601 | 0.727 | -0.298 | 1.715 | 0.862 |
| Metformin | Vildagliptin | 6/1,103 | -0.604 | 0.799 | -0.433 | 0.617 | -0.172 | 1.010 | 0.865 |
| Saxagliptin | Sulfonylurea | 9/2,346 | -0.129 | 0.675 | 0.119 | 0.244 | -0.248 | 0.717 | 0.729 |
| Sitagliptin | Sulfonylurea | 30/2,757 | 0.898 | 0.425 | -0.103 | 0.263 | 1.001 | 0.500 | **0.0450** |
| Sitagliptin | Thiazolidinedione | 3/1,344 | -0.906 | 0.935 | -0.0619 | 0.615 | -0.844 | 1.107 | 0.446 |
| Sulfonylurea | Vildagliptin | 28/4,171 | -0.138 | 0.384 | -0.644 | 0.491 | 0.506 | 0.624 | 0.417 |
| Thiazolidinedione | Vildagliptin | 1/782 | 0.445 | 1.635 | 0.115 | 0.625 | 0.329 | 1.750 | 0.851 |

# Additional file 1: S11. Summaries of sensitivity analysis

| Drug | Standard analysis | SUCRA rank | Excluding studies with cardiovascular high risk | SUCRA rank | Excluding studies with high risk or renal impairment | SUCRA rank | Excluding studies with one arm <100 patients | SUCRA rank |
| --- | --- | --- | --- | --- | --- | --- | --- | --- |
| MACE | | | | | | | | |
| SGLT2i | 0.70 (0.55,0.90) | 1 | 0.68 (0.48,0.96) | 1 | 0.68 (0.47,0.98) | 1 | 0.71 (0.55,0.91) | 2 |
| INS | 0.71 (0.57,0.90) | 2 | 0.76 (0.55,1.05) | 3 | 0.77 (0.55,1.07) | 3 | 0.71 (0.56,0.90) | 1 |
| GLP1a | 0.76 (0.61,0.94) | 3 | 0.73 (0.56,0.95) | 2 | 0.70 (0.54,0.93) | 2 | 0.75 (0.60,0.94) | 3 |
| DPP4i | 0.77 (0.62,0.96) | 4 | 0.95 (0.69,1.30) | 5 | 0.96 (0.69,1.35) | 6 | 0.77 (0.61,0.96) | 4 |
| PLA | 0.88 (0.65,1.19) | 5 | 1.03 (0.72,1.46) | 7 | 1.02 (0.72,1.45) | 7 | 0.91 (0.67,1.23) | 5 |
| MET | 0.93 (0.75,1.16) | 6 | 0.95 (0.76,1.19) | 6 | 0.95 (0.76,1.19) | 5 | 0.93 (0.75,1.15) | 6 |
| TZD | 0.97 (0.80,1.18) | 7 | 0.96 (0.78,1.18) | 4 | 0.96 (0.77,1.18) | 4 | 0.97 (0.80,1.18) | 7 |
| SU | Reference | 8 | Reference | 8 | Reference | 8 | Reference | 8 |
| All-cause mortality | | | | | | | | |
| SGLT2i | 0.58 (0.41,0.83) | 1 | 0.79 (0.47,1.34) | 3 | 0.88 (0.50,1.57) | 3 | 0.59 (0.41,0.85) | 1 |
| INS | 0.70 (0.50,0.97) | 2 | 0.63 (0.38,1.04) | 1 | 0.61 (0.36,1.04) | 1 | 0.71 (0.51,0.99) | 2 |
| DPP4i | 0.81 (0.59,1.10) | 3 | 0.97 (0.62,1.51) | 5 | 0.93 (0.57,1.53) | 5 | 0.82 (0.59,1.14) | 3 |
| GLP1a | 0.81 (0.60,1.12) | 4 | 0.76 (0.54,1.08) | 2 | 0.81 (0.55,1.19) | 2 | 0.83 (0.60,1.16) | 5 |
| PLA | 0.84 (0.59,1.22) | 5 | 0.97 (0.65,1.44) | 6 | 0.98 (0.66,1.46) | 6 | 0.85 (0.59,1.23) | 4 |
| TZD | 0.91 (0.72,1.15) | 6 | 0.92 (0.72,1.18) | 4 | 0.92 (0.72,1.18) | 4 | 0.91 (0.72,1.16) | 6 |
| SU | Reference | 7 | Reference | 7 | Reference | 7 | Reference | 7 |
| MET | 1.04 (0.81,1.34) | 8 | 1.03 (0.81,1.32) | 8 | 1.03 (0.80,1.32) | 8 | 1.04 (0.81,1.33) | 8 |

| Drug | Standard analysis | SUCRA rank | Excluding studies with high cardiovascular risk | SUCRA rank | Excluding studies with high cardiovascular risk or renal impairment | SUCRA rank | Excluding studies with one arm <100 patients | SUCRA rank |
| --- | --- | --- | --- | --- | --- | --- | --- | --- |
| MACE | | | | | | | | |
| VIL | 0.47 (0.25,0.90) | 1 | 0.50 (0.26,0.96) | 1 | 0.50 (0.26,0.96) | 1 | 0.47 (0.25,0.90) | 1 |
| EMP | 0.53 (0.28,1.04) | 2 | 0.55 (0.28,1.07) | 2 | 0.55 (0.28,1.07) | 2 | 0.53 (0.27,1.03) | 2 |
| LIX | 0.72 (0.55,0.95) | 3 | 0.82 (0.44,1.53) | 11 | 0.82 (0.44,1.53) | 11 | 0.72 (0.55,0.95) | 3 |
| EXE | 0.74 (0.56,0.99) | 4 | 0.64 (0.41,1.01) | 3 | 0.64 (0.40,1.02) | 3 | 0.75 (0.57,1.01) | 5 |
| LIR | 0.73 (0.45,1.17) | 5 | 0.79 (0.49,1.29) | 8 | 0.79 (0.47,1.34) | 8 | 0.67 (0.39,1.17) | 4 |
| ALO | 0.74 (0.46,1.20) | 6 | 0.78 (0.48,1.27) | 7 | 0.81 (0.48,1.35) | 9 | 0.74 (0.46,1.20) | 6 |
| CAN | 0.78 (0.58,1.03) | 7 | 0.76 (0.41,1.40) | 6 | 0.76 (0.41,1.41) | 6 | 0.77 (0.58,1.03) | 7 |
| DAP | 0.75 (0.44,1.29) | 8 | 0.81 (0.47,1.40) | 10 | 0.82 (0.46,1.49) | 10 | 0.78 (0.43,1.39) | 8 |
| DUL | 0.80 (0.47,1.35) | 9 | 0.66 (0.30,1.43) | 5 | 0.67 (0.28,1.59) | 5 | 0.80 (0.46,1.40) | 10 |
| SIT | 0.81 (0.62,1.05) | 10 | 0.80 (0.54,1.19) | 9 | 0.78 (0.49,1.22) | 7 | 0.80 (0.61,1.05) | 9 |
| ALB | 0.83 (0.64,1.06) | 11 | 0.97 (0.69,1.35) | 16 | 0.98 (0.69,1.39) | 16 | 0.82 (0.63,1.07) | 11 |
| SAX | 0.83 (0.63,1.09) | 12 | 0.69 (0.41,1.17) | 4 | 0.63 (0.36,1.10) | 4 | 0.83 (0.63,1.10) | 12 |
| PLA | 0.87 (0.64,1.17) | 13 | 1.01 (0.71,1.44) | 17 | 1.01 (0.71,1.44) | 17 | 0.89 (0.66,1.22) | 15 |
| MET | 0.84 (0.63,1.13) | 14 | 1.11 (0.31,3.95) | 14 | 1.13 (0.32,4.01) | 15 | 0.84 (0.62,1.14) | 13 |
| INS | 0.90 (0.51,1.59) | 15 | 0.91 (0.51,1.62) | 12 | 0.91 (0.51,1.62) | 12 | 0.89 (0.50,1.58) | 14 |
| LIN | 0.93 (0.75,1.17) | 16 | 0.95 (0.76,1.18) | 13 | 0.95 (0.76,1.18) | 13 | 0.93 (0.74,1.16) | 16 |
| TZD | 0.97 (0.80,1.18) | 17 | 0.96 (0.77,1.18) | 15 | 0.96 (0.77,1.18) | 14 | 0.97 (0.80,1.18) | 17 |
| SU | Reference | 18 | Reference | 18 | 1.41 (0.65,3.06) | 18 | Reference | 18 |
| All-cause mortality | | | | | | | | |
| EXE | 0.57 (0.38,0.85) | 1 | 0.78 (0.35,1.72) | 10 | 0.87 (0.38,2.00) | 10 | 0.58 (0.38,0.89) | 1 |
| ALO | 0.54 (0.23,1.30) | 2 | 0.52 (0.22,1.27) | 2 | 0.43 (0.15,1.28) | 1 | 0.55 (0.23,1.32) | 3 |
| EMP | 0.46 (0.13,1.59) | 3 | 0.45 (0.13,1.55) | 1 | 0.47 (0.13,1.65) | 3 | 0.46 (0.13,1.60) | 2 |
| LIX | 0.70 (0.47,1.03) | 4 | 0.61 (0.14,2.61) | 5 | 0.63 (0.14,2.74) | 4 | 0.71 (0.47,1.08) | 4 |
| LIR | 0.68 (0.31,1.49) | 5 | 0.75 (0.34,1.67) | 9 | 0.71 (0.30,1.71) | 5 | 0.77 (0.31,1.93) | 9 |
| CAN | 0.73 (0.48,1.10) | 6 | 0.92 (0.30,2.86) | 12 | 0.92 (0.29,2.85) | 14 | 0.75 (0.48,1.15) | 5 |
| VIL | 0.70 (0.40,1.23) | 7 | 0.73 (0.41,1.30) | 7 | 0.77 (0.42,1.42) | 8 | 0.72 (0.41,1.28) | 7 |
| DAP | 0.71 (0.36,1.42) | 8 | 1.04 (0.40,2.72) | 16 | 1.15 (0.37,3.56) | 17 | 0.71 (0.34,1.49) | 6 |
| MET | 0.77 (0.51,1.17) | 9 | 0.50 (0.13,1.88) | 3 | 0.48 (0.12,1.85) | 2 | 0.79 (0.51,1.22) | 8 |
| DUL | 0.81 (0.42,1.58) | 10 | 0.62 (0.28,1.40) | 4 | 0.72 (0.28,1.85) | 6 | 0.98 (0.46,2.09) | 15 |
| INS | 0.83 (0.41,1.68) | 11 | 0.82 (0.40,1.68) | 11 | 0.82 (0.40,1.69) | 9 | 0.83 (0.41,1.69) | 10 |
| ALB | 0.83 (0.57,1.20) | 12 | 0.92 (0.58,1.48) | 14 | 0.89 (0.53,1.49) | 11 | 0.85 (0.58,1.25) | 12 |
| SIT | 0.84 (0.58,1.22) | 13 | 0.73 (0.42,1.25) | 6 | 0.91 (0.44,1.85) | 12 | 0.86 (0.58,1.28) | 13 |
| PLA | 0.87 (0.60,1.25) | 14 | 0.96 (0.65,1.43) | 15 | 0.98 (0.66,1.45) | 15 | 0.86 (0.59,1.24) | 11 |
| TZD | 0.91 (0.72,1.16) | 15 | 0.92 (0.71,1.18) | 13 | 0.92 (0.71,1.18) | 13 | 0.91 (0.72,1.16) | 14 |
| SAX | 0.91 (0.62,1.34) | 16 | 0.74 (0.33,1.63) | 8 | 0.74 (0.31,1.78) | 7 | 0.94 (0.62,1.41) | 16 |
| SU | Reference | 17 | Reference | 17 | Reference | 16 | Reference | 17 |
| LIN | 1.03 (0.81,1.32) | 18 | 1.03 (0.80,1.32) | 18 | 1.03 (0.80,1.32) | 18 | 1.03 (0.80,1.32) | 18 |

**Reference list of included studies**

**1.** Zannad F, Cannon CP, Cushman WC, et al. Heart failure and mortality outcomes in patients with type 2 diabetes taking alogliptin versus placebo in EXAMINE: a multicentre, randomised, double-blind trial. *Lancet.* 2015;385(9982):2067-2076.

**2.** White WB, Cannon CP, Heller SR, et al. Alogliptin after acute coronary syndrome in patients with type 2 diabetes. *N Engl J Med.* 2013;369(14):1327-1335.

**3.** Del PS, Camisasca R, Wilson C, Fleck P. Durability of the efficacy and safety of alogliptin compared with glipizide in type 2 diabetes mellitus: a 2-year study. *Diabetes Obes Metab.* 2014;16(12):1239-1246.

**4.** Pratley RE, Fleck P, Wilson C. Efficacy and safety of initial combination therapy with alogliptin plus metformin versus either as monotherapy in drug-naive patients with type 2 diabetes: a randomized, double-blind, 6-month study. *Diabetes Obes Metab.* 2014;16(7):613-621.

**5.** Rosenstock J, Wilson C, Fleck P. Alogliptin versus glipizide monotherapy in elderly type 2 diabetes mellitus patients with mild hyperglycaemia: a prospective, double-blind, randomized, 1-year study. *Diabetes Obes Metab.* 2013;15(10):906-914.

**6.** DeFronzo RA, Burant CF, Fleck P, Wilson C, Mekki Q, Pratley RE. Efficacy and tolerability of the DPP-4 inhibitor alogliptin combined with pioglitazone, in metformin-treated patients with type 2 diabetes. *J Clin Endocrinol Metab.* 2012;97(5):1615-1622.

**7.** Bosi E, Ellis GC, Wilson CA, Fleck PR. Alogliptin as a third oral antidiabetic drug in patients with type 2 diabetes and inadequate glycaemic control on metformin and pioglitazone: a 52-week, randomized, double-blind, active-controlled, parallel-group study. *Diabetes Obes Metab.* 2011;13(12):1088-1096.

**8.** Kaku K, Itayasu T, Hiroi S, Hirayama M, Seino Y. Efficacy and safety of alogliptin added to pioglitazone in Japanese patients with type 2 diabetes: a randomized, double-blind, placebo-controlled trial with an open-label long-term extension study. *Diabetes Obes Metab.* 2011;13(11):1028-1035.

**9.** Nauck MA, Ellis GC, Fleck PR, Wilson CA, Mekki Q. Efficacy and safety of adding the dipeptidyl peptidase-4 inhibitor alogliptin to metformin therapy in patients with type 2 diabetes inadequately controlled with metformin monotherapy: a multicentre, randomised, double-blind, placebo-controlled study. *Int J Clin Pract.* 2009;63(1):46-55.

**10.** Pratley RE, Reusch JE, Fleck PR, Wilson CA, Mekki Q. Efficacy and safety of the dipeptidyl peptidase-4 inhibitor alogliptin added to pioglitazone in patients with type 2 diabetes: a randomized, double-blind, placebo-controlled study. *Curr Med Res Opin.* 2009;25(10):2361-2371.

**11.** Rosenstock J, Rendell MS, Gross JL, Fleck PR, Wilson CA, Mekki Q. Alogliptin added to insulin therapy in patients with type 2 diabetes reduces HbA(1C) without causing weight gain or increased hypoglycaemia. *Diabetes Obes Metab.* 2009;11(12):1145-1152.

**12.** Rosenstock J, Inzucchi SE, Seufert J, Fleck PR, Wilson CA, Mekki Q. Initial combination therapy with alogliptin and pioglitazone in drug-naive patients with type 2 diabetes. *Diabetes Care.* 2010;33(11):2406-2408.

**13.** Bajaj M, Gilman R, Patel S, Kempthorne-Rawson J, Lewis-D'Agostino D, Woerle HJ. Linagliptin improved glycaemic control without weight gain or hypoglycaemia in patients with type 2 diabetes inadequately controlled by a combination of metformin and pioglitazone: a 24-week randomized, double-blind study. *Diabet Med.* 2014;31(12):1505-1514.

**14.** Barnett AH, Huisman H, Jones R, von Eynatten M, Patel S, Woerle HJ. Linagliptin for patients aged 70 years or older with type 2 diabetes inadequately controlled with common antidiabetes treatments: a randomised, double-blind, placebo-controlled trial. *Lancet.* 2013;382(9902):1413-1423.

**15.** McGill JB, Sloan L, Newman J, et al. Long-term efficacy and safety of linagliptin in patients with type 2 diabetes and severe renal impairment: a 1-year, randomized, double-blind, placebo-controlled study. *Diabetes Care.* 2013;36(2):237-244.

**16.** Yki-Jarvinen H, Rosenstock J, Duran-Garcia S, et al. Effects of adding linagliptin to basal insulin regimen for inadequately controlled type 2 diabetes: a >/=52-week randomized, double-blind study. *Diabetes Care.* 2013;36(12):3875-3881.

**17.** Gallwitz B, Rosenstock J, Rauch T, et al. 2-year efficacy and safety of linagliptin compared with glimepiride in patients with type 2 diabetes inadequately controlled on metformin: a randomised, double-blind, non-inferiority trial. *Lancet.* 2012;380(9840):475-483.

**18.** Gomis R, Espadero RM, Jones R, Woerle HJ, Dugi KA. Efficacy and safety of initial combination therapy with linagliptin and pioglitazone in patients with inadequately controlled type 2 diabetes: a randomized, double-blind, placebo-controlled study. *Diabetes Obes Metab.* 2011;13(7):653-661.

**19.** Haak T, Meinicke T, Jones R, Weber S, von Eynatten M, Woerle HJ. Initial combination of linagliptin and metformin improves glycaemic control in type 2 diabetes: a randomized, double-blind, placebo-controlled study. *Diabetes Obes Metab.* 2012;14(6):565-574.

**20.** DeFronzo RA, Lewin A, Patel S, et al. Combination of empagliflozin and linagliptin as second-line therapy in subjects with type 2 diabetes inadequately controlled on metformin. *Diabetes Care.* 2015;38(3):384-393.

**21.** Lewin A, DeFronzo RA, Patel S, et al. Initial combination of empagliflozin and linagliptin in subjects with type 2 diabetes. *Diabetes Care.* 2015;38(3):394-402.

**22.** Inagaki N, Watada H, Murai M, et al. Linagliptin provides effective, well-tolerated add-on therapy to pre-existing oral antidiabetic therapy over 1 year in Japanese patients with type 2 diabetes. *Diabetes Obes Metab.* 2013;15(9):833-843.

**23.** Owens DR, Swallow R, Dugi KA, Woerle HJ. Efficacy and safety of linagliptin in persons with type 2 diabetes inadequately controlled by a combination of metformin and sulphonylurea: a 24-week randomized study. *Diabet Med.* 2011;28(11):1352-1361.

**24.** Taskinen MR, Rosenstock J, Tamminen I, et al. Safety and efficacy of linagliptin as add-on therapy to metformin in patients with type 2 diabetes: a randomized, double-blind, placebo-controlled study. *Diabetes Obes Metab.* 2011;13(1):65-74.

**25.** Schernthaner G, Duran-Garcia S, Hanefeld M, et al. Efficacy and tolerability of saxagliptin compared with glimepiride in elderly patients with type 2 diabetes: a randomized, controlled study (GENERATION). *Diabetes Obes Metab.* 2015;17(7):630-638.

**26.** Barnett AH, Charbonnel B, Li J, Donovan M, Fleming D, Iqbal N. Saxagliptin add-on therapy to insulin with or without metformin for type 2 diabetes mellitus: 52-week safety and efficacy. *Clin Drug Investig.* 2013;33(10):707-717.

**27.** Barnett AH, Charbonnel B, Donovan M, Fleming D, Chen R. Effect of saxagliptin as add-on therapy in patients with poorly controlled type 2 diabetes on insulin alone or insulin combined with metformin. *Curr Med Res Opin.* 2012;28(4):513-523.

**28.** Goke B, Gallwitz B, Eriksson JG, Hellqvist A, Gause-Nilsson I. Saxagliptin vs. glipizide as add-on therapy in patients with type 2 diabetes mellitus inadequately controlled on metformin alone: long-term (52-week) extension of a 52-week randomised controlled trial. *Int J Clin Pract.* 2013;67(4):307-316.

**29.** Goke B, Gallwitz B, Eriksson J, Hellqvist A, Gause-Nilsson I. Saxagliptin is non-inferior to glipizide in patients with type 2 diabetes mellitus inadequately controlled on metformin alone: a 52-week randomised controlled trial. *Int J Clin Pract.* 2010;64(12):1619-1631.

**30.** Rosenstock J, Gross JL, Aguilar-Salinas C, et al. Long-term 4-year safety of saxagliptin in drug-naive and metformin-treated patients with Type 2 diabetes. *Diabet Med.* 2013;30(12):1472-1476.

**31.** Rosenstock J, Aguilar-Salinas C, Klein E, Nepal S, List J, Chen R. Effect of saxagliptin monotherapy in treatment-naive patients with type 2 diabetes. *Curr Med Res Opin.* 2009;25(10):2401-2411.

**32.** Scirica BM, Bhatt DL, Braunwald E, et al. Saxagliptin and cardiovascular outcomes in patients with type 2 diabetes mellitus. *N Engl J Med.* 2013;369(14):1317-1326.

**33.** Pan CY, Yang W, Tou C, Gause-Nilsson I, Zhao J. Efficacy and safety of saxagliptin in drug-naive Asian patients with type 2 diabetes mellitus: a randomized controlled trial. *Diabetes Metab Res Rev.* 2012;28(3):268-275.

**34.** Yang W, Pan CY, Tou C, Zhao J, Gause-Nilsson I. Efficacy and safety of saxagliptin added to metformin in Asian people with type 2 diabetes mellitus: a randomized controlled trial. *Diabetes Res Clin Pract.* 2011;94(2):217-224.

**35.** Chacra AR, Tan GH, Apanovitch A, Ravichandran S, List J, Chen R. Saxagliptin added to a submaximal dose of sulphonylurea improves glycaemic control compared with uptitration of sulphonylurea in patients with type 2 diabetes: a randomised controlled trial. *Int J Clin Pract.* 2009;63(9):1395-1406.

**36.** DeFronzo RA, Hissa MN, Garber AJ, et al. The efficacy and safety of saxagliptin when added to metformin therapy in patients with inadequately controlled type 2 diabetes with metformin alone. *Diabetes Care.* 2009;32(9):1649-1655.

**37.** Hollander PL, Li J, Frederich R, Allen E, Chen R. Safety and efficacy of saxagliptin added to thiazolidinedione over 76 weeks in patients with type 2 diabetes mellitus. *Diab Vasc Dis Res.* 2011;8(2):125-135.

**38.** Hollander P, Li J, Allen E, Chen R. Saxagliptin added to a thiazolidinedione improves glycemic control in patients with type 2 diabetes and inadequate control on thiazolidinedione alone. *J Clin Endocrinol Metab.* 2009;94(12):4810-4819.

**39.** Pfutzner A, Paz-Pacheco E, Allen E, Frederich R, Chen R. Initial combination therapy with saxagliptin and metformin provides sustained glycaemic control and is well tolerated for up to 76 weeks. *Diabetes Obes Metab.* 2011;13(6):567-576.

**40.** Jadzinsky M, Pfutzner A, Paz-Pacheco E, Xu Z, Allen E, Chen R. Saxagliptin given in combination with metformin as initial therapy improves glycaemic control in patients with type 2 diabetes compared with either monotherapy: a randomized controlled trial. *Diabetes Obes Metab.* 2009;11(6):611-622.

**41.** Nowicki M, Rychlik I, Haller H, et al. Long-term treatment with the dipeptidyl peptidase-4 inhibitor saxagliptin in patients with type 2 diabetes mellitus and renal impairment: a randomised controlled 52-week efficacy and safety study. *Int J Clin Pract.* 2011;65(12):1230-1239.

**42.** Hermans MP, Delibasi T, Farmer I, et al. Effects of saxagliptin added to sub-maximal doses of metformin compared with uptitration of metformin in type 2 diabetes: the PROMPT study. *Curr Med Res Opin.* 2012;28(10):1635-1645.

**43.** Haering HU, Merker L, Christiansen AV, et al. Empagliflozin as add-on to metformin plus sulphonylurea in patients with type 2 diabetes. *Diabetes Res Clin Pract.* 2015;110(1):82-90.

**44.** Roden M, Merker L, Christiansen AV, et al. Safety, tolerability and effects on cardiometabolic risk factors of empagliflozin monotherapy in drug-naive patients with type 2 diabetes: a double-blind extension of a Phase III randomized controlled trial. *Cardiovasc Diabetol.* 2015;14:154.

**45.** Green JB, Bethel MA, Armstrong PW, et al. Effect of Sitagliptin on Cardiovascular Outcomes in Type 2 Diabetes. *N Engl J Med.* 2015;373(3):232-242.

**46.** Henry RR, Staels B, Fonseca VA, et al. Efficacy and safety of initial combination treatment with sitagliptin and pioglitazone--a factorial study. *Diabetes Obes Metab.* 2014;16(3):223-230.

**47.** Leiter LA, Carr MC, Stewart M, et al. Efficacy and safety of the once-weekly GLP-1 receptor agonist albiglutide versus sitagliptin in patients with type 2 diabetes and renal impairment: a randomized phase III study. *Diabetes Care.* 2014;37(10):2723-2730.

**48.** Weinstock RS, Guerci B, Umpierrez G, Nauck MA, Skrivanek Z, Milicevic Z. Safety and efficacy of once-weekly dulaglutide versus sitagliptin after 2 years in metformin-treated patients with type 2 diabetes (AWARD-5): a randomized, phase III study. *Diabetes Obes Metab.* 2015;17(9):849-858.

**49.** Nauck M, Weinstock RS, Umpierrez GE, Guerci B, Skrivanek Z, Milicevic Z. Efficacy and safety of dulaglutide versus sitagliptin after 52 weeks in type 2 diabetes in a randomized controlled trial (AWARD-5). *Diabetes Care.* 2014;37(8):2149-2158.

**50.** Schernthaner G, Gross JL, Rosenstock J, et al. Canagliflozin compared with sitagliptin for patients with type 2 diabetes who do not have adequate glycemic control with metformin plus sulfonylurea: a 52-week randomized trial. *Diabetes Care.* 2013;36(9):2508-2515.

**51.** Roden M, Weng J, Eilbracht J, et al. Empagliflozin monotherapy with sitagliptin as an active comparator in patients with type 2 diabetes: a randomised, double-blind, placebo-controlled, phase 3 trial. *Lancet Diabetes Endocrinol.* 2013;1(3):208-219.

**52.** Arjona FJ, Corry D, Mogensen CE, et al. Efficacy and safety of sitagliptin in patients with type 2 diabetes and ESRD receiving dialysis: a 54-week randomized trial. *Am J Kidney Dis.* 2013;61(4):579-587.

**53.** Arjona FJ, Marre M, Barzilai N, et al. Efficacy and safety of sitagliptin versus glipizide in patients with type 2 diabetes and moderate-to-severe chronic renal insufficiency. *Diabetes Care.* 2013;36(5):1067-1073.

**54.** Dobs AS, Goldstein BJ, Aschner P, et al. Efficacy and safety of sitagliptin added to ongoing metformin and rosiglitazone combination therapy in a randomized placebo-controlled 54-week trial in patients with type 2 diabetes. *J Diabetes.* 2013;5(1):68-79.

**55.** Fonseca V, Staels B, Morgan JN, et al. Efficacy and safety of sitagliptin added to ongoing metformin and pioglitazone combination therapy in a randomized, placebo-controlled, 26-week trial in patients with type 2 diabetes. *J Diabetes Complications.* 2013;27(2):177-183.

**56.** Philis-Tsimikas A, Del PS, Satman I, et al. Effect of insulin degludec versus sitagliptin in patients with type 2 diabetes uncontrolled on oral antidiabetic agents. *Diabetes Obes Metab.* 2013;15(8):760-766.

**57.** Aschner P, Chan J, Owens DR, et al. Insulin glargine versus sitagliptin in insulin-naive patients with type 2 diabetes mellitus uncontrolled on metformin (EASIE): a multicentre, randomised open-label trial. *Lancet.* 2012;379(9833):2262-2269.

**58.** Arechavaleta R, Seck T, Chen Y, et al. Efficacy and safety of treatment with sitagliptin or glimepiride in patients with type 2 diabetes inadequately controlled on metformin monotherapy: a randomized, double-blind, non-inferiority trial. *Diabetes Obes Metab.* 2011;13(2):160-168.

**59.** Pratley R, Nauck M, Bailey T, et al. One year of liraglutide treatment offers sustained and more effective glycaemic control and weight reduction compared with sitagliptin, both in combination with metformin, in patients with type 2 diabetes: a randomised, parallel-group, open-label trial. *Int J Clin Pract.* 2011;65(4):397-407.

**60.** Pratley RE, Nauck M, Bailey T, et al. Liraglutide versus sitagliptin for patients with type 2 diabetes who did not have adequate glycaemic control with metformin: a 26-week, randomised, parallel-group, open-label trial. *Lancet.* 2010;375(9724):1447-1456.

**61.** Bergenstal RM, Wysham C, Macconell L, et al. Efficacy and safety of exenatide once weekly versus sitagliptin or pioglitazone as an adjunct to metformin for treatment of type 2 diabetes (DURATION-2): a randomised trial. *Lancet.* 2010;376(9739):431-439.

**62.** Seck T, Nauck M, Sheng D, et al. Safety and efficacy of treatment with sitagliptin or glipizide in patients with type 2 diabetes inadequately controlled on metformin: a 2-year study. *Int J Clin Pract.* 2010;64(5):562-576.

**63.** Nauck MA, Meininger G, Sheng D, Terranella L, Stein PP. Efficacy and safety of the dipeptidyl peptidase-4 inhibitor, sitagliptin, compared with the sulfonylurea, glipizide, in patients with type 2 diabetes inadequately controlled on metformin alone: a randomized, double-blind, non-inferiority trial. *Diabetes Obes Metab.* 2007;9(2):194-205.

**64.** Vilsboll T, Rosenstock J, Yki-Jarvinen H, et al. Efficacy and safety of sitagliptin when added to insulin therapy in patients with type 2 diabetes. *Diabetes Obes Metab.* 2010;12(2):167-177.

**65.** Raz I, Chen Y, Wu M, et al. Efficacy and safety of sitagliptin added to ongoing metformin therapy in patients with type 2 diabetes. *Curr Med Res Opin.* 2008;24(2):537-550.

**66.** Williams-Herman D, Johnson J, Teng R, et al. Efficacy and safety of sitagliptin and metformin as initial combination therapy and as monotherapy over 2 years in patients with type 2 diabetes. *Diabetes Obes Metab.* 2010;12(5):442-451.

**67.** Williams-Herman D, Johnson J, Teng R, et al. Efficacy and safety of initial combination therapy with sitagliptin and metformin in patients with type 2 diabetes: a 54-week study. *Curr Med Res Opin.* 2009;25(3):569-583.

**68.** Goldstein BJ, Feinglos MN, Lunceford JK, Johnson J, Williams-Herman DE. Effect of initial combination therapy with sitagliptin, a dipeptidyl peptidase-4 inhibitor, and metformin on glycemic control in patients with type 2 diabetes. *Diabetes Care.* 2007;30(8):1979-1987.

**69.** Hartley P, Shentu Y, Betz-Schiff P, et al. Efficacy and Tolerability of Sitagliptin Compared with Glimepiride in Elderly Patients with Type 2 Diabetes Mellitus and Inadequate Glycemic Control: A Randomized, Double-Blind, Non-Inferiority Trial. *Drugs Aging.* 2015;32(6):469-476.

**70.** Ahren B, Johnson SL, Stewart M, et al. HARMONY 3: 104-week randomized, double-blind, placebo- and active-controlled trial assessing the efficacy and safety of albiglutide compared with placebo, sitagliptin, and glimepiride in patients with type 2 diabetes taking metformin. *Diabetes Care.* 2014;37(8):2141-2148.

**71.** Lavalle-Gonzalez FJ, Januszewicz A, Davidson J, et al. Efficacy and safety of canagliflozin compared with placebo and sitagliptin in patients with type 2 diabetes on background metformin monotherapy: a randomised trial. *Diabetologia.* 2013;56(12):2582-2592.

**72.** Russell-Jones D, Cuddihy RM, Hanefeld M, et al. Efficacy and safety of exenatide once weekly versus metformin, pioglitazone, and sitagliptin used as monotherapy in drug-naive patients with type 2 diabetes (DURATION-4): a 26-week double-blind study. *Diabetes Care.* 2012;35(2):252-258.

**73.** Yoon KH, Shockey GR, Teng R, et al. Effect of initial combination therapy with sitagliptin, a dipeptidyl peptidase-4 inhibitor, and pioglitazone on glycemic control and measures of beta-cell function in patients with type 2 diabetes. *Int J Clin Pract.* 2011;65(2):154-164.

**74.** Aschner P, Katzeff HL, Guo H, et al. Efficacy and safety of monotherapy of sitagliptin compared with metformin in patients with type 2 diabetes. *Diabetes Obes Metab.* 2010;12(3):252-261.

**75.** Hermansen K, Kipnes M, Luo E, Fanurik D, Khatami H, Stein P. Efficacy and safety of the dipeptidyl peptidase-4 inhibitor, sitagliptin, in patients with type 2 diabetes mellitus inadequately controlled on glimepiride alone or on glimepiride and metformin. *Diabetes Obes Metab.* 2007;9(5):733-745.

**76.** Ferrannini E, Berk A, Hantel S, et al. Long-term safety and efficacy of empagliflozin, sitagliptin, and metformin: an active-controlled, parallel-group, randomized, 78-week open-label extension study in patients with type 2 diabetes. *Diabetes Care.* 2013;36(12):4015-4021.

**77.** Yang W, Xing X, Lv X, et al. Vildagliptin added to sulfonylurea improves glycemic control without hypoglycemia and weight gain in Chinese patients with type 2 diabetes mellitus. *J Diabetes.* 2015;7(2):174-181.

**78.** Strain WD, Lukashevich V, Kothny W, Hoellinger MJ, Paldanius PM. Individualised treatment targets for elderly patients with type 2 diabetes using vildagliptin add-on or lone therapy (INTERVAL): a 24 week, randomised, double-blind, placebo-controlled study. *Lancet.* 2013;382(9890):409-416.

**79.** Pan C, Xing X, Han P, et al. Efficacy and tolerability of vildagliptin as add-on therapy to metformin in Chinese patients with type 2 diabetes mellitus. *Diabetes Obes Metab.* 2012;14(8):737-744.

**80.** Bolli G, Dotta F, Colin L, Minic B, Goodman M. Comparison of vildagliptin and pioglitazone in patients with type 2 diabetes inadequately controlled with metformin. *Diabetes Obes Metab.* 2009;11(6):589-595.

**81.** Bolli G, Dotta F, Rochotte E, Cohen SE. Efficacy and tolerability of vildagliptin vs. pioglitazone when added to metformin: a 24-week, randomized, double-blind study. *Diabetes Obes Metab.* 2008;10(1):82-90.

**82.** Bosi E, Dotta F, Jia Y, Goodman M. Vildagliptin plus metformin combination therapy provides superior glycaemic control to individual monotherapy in treatment-naive patients with type 2 diabetes mellitus. *Diabetes Obes Metab.* 2009;11(5):506-515.

**83.** Ferrannini E, Fonseca V, Zinman B, et al. Fifty-two-week efficacy and safety of vildagliptin vs. glimepiride in patients with type 2 diabetes mellitus inadequately controlled on metformin monotherapy. *Diabetes Obes Metab.* 2009;11(2):157-166.

**84.** Matthews DR, Dejager S, Ahren B, et al. Vildagliptin add-on to metformin produces similar efficacy and reduced hypoglycaemic risk compared with glimepiride, with no weight gain: results from a 2-year study. *Diabetes Obes Metab.* 2010;12(9):780-789.

**85.** Schweizer A, Dejager S, Bosi E. Comparison of vildagliptin and metformin monotherapy in elderly patients with type 2 diabetes: a 24-week, double-blind, randomized trial. *Diabetes Obes Metab.* 2009;11(8):804-812.

**86.** Bosi E, Camisasca RP, Collober C, Rochotte E, Garber AJ. Effects of vildagliptin on glucose control over 24 weeks in patients with type 2 diabetes inadequately controlled with metformin. *Diabetes Care.* 2007;30(4):890-895.

**87.** Fonseca V, Schweizer A, Albrecht D, Baron MA, Chang I, Dejager S. Addition of vildagliptin to insulin improves glycaemic control in type 2 diabetes. *Diabetologia.* 2007;50(6):1148-1155.

**88.** Schweizer A, Couturier A, Foley JE, Dejager S. Comparison between vildagliptin and metformin to sustain reductions in HbA(1c) over 1 year in drug-naive patients with Type 2 diabetes. *Diabet Med.* 2007;24(9):955-961.

**89.** Forst T, Koch C, Dworak M. Vildagliptin versus insulin in patients with type 2 diabetes mellitus inadequately controlled with sulfonylurea: results from a randomized, 24 week study. *Curr Med Res Opin.* 2015;31(6):1079-1084.

**90.** Kothny W, Shao Q, Groop PH, Lukashevich V. One-year safety, tolerability and efficacy of vildagliptin in patients with type 2 diabetes and moderate or severe renal impairment. *Diabetes Obes Metab.* 2012;14(11):1032-1039.

**91.** Lukashevich V, Schweizer A, Shao Q, Groop PH, Kothny W. Safety and efficacy of vildagliptin versus placebo in patients with type 2 diabetes and moderate or severe renal impairment: a prospective 24-week randomized placebo-controlled trial. *Diabetes Obes Metab.* 2011;13(10):947-954.

**92.** Foley JE, Sreenan S. Efficacy and safety comparison between the DPP-4 inhibitor vildagliptin and the sulfonylurea gliclazide after two years of monotherapy in drug-naive patients with type 2 diabetes. *Horm Metab Res.* 2009;41(12):905-909.

**93.** Rosenstock J, Baron MA, Dejager S, Mills D, Schweizer A. Comparison of vildagliptin and rosiglitazone monotherapy in patients with type 2 diabetes: a 24-week, double-blind, randomized trial. *Diabetes Care.* 2007;30(2):217-223.

**94.** Nauck MA, Stewart MW, Perkins C, et al. Efficacy and safety of once-weekly GLP-1 receptor agonist albiglutide (HARMONY 2): 52 week primary endpoint results from a randomised, placebo-controlled trial in patients with type 2 diabetes mellitus inadequately controlled with diet and exercise. *Diabetologia.* 2016;59(2):266-274.

**95.** Home PD, Shamanna P, Stewart M, et al. Efficacy and tolerability of albiglutide versus placebo or pioglitazone over 1 year in people with type 2 diabetes currently taking metformin and glimepiride: HARMONY 5. *Diabetes Obes Metab.* 2015;17(2):179-187.

**96.** Reusch J, Stewart MW, Perkins CM, et al. Efficacy and safety of once-weekly glucagon-like peptide 1 receptor agonist albiglutide (HARMONY 1 trial): 52-week primary endpoint results from a randomized, double-blind, placebo-controlled trial in patients with type 2 diabetes mellitus not controlled on pioglitazone, with or without metformin. *Diabetes Obes Metab.* 2014;16(12):1257-1264.

**97.** Rosenstock J, Fonseca VA, Gross JL, et al. Advancing basal insulin replacement in type 2 diabetes inadequately controlled with insulin glargine plus oral agents: a comparison of adding albiglutide, a weekly GLP-1 receptor agonist, versus thrice-daily prandial insulin lispro. *Diabetes Care.* 2014;37(8):2317-2325.

**98.** Weissman PN, Carr MC, Ye J, et al. HARMONY 4: randomised clinical trial comparing once-weekly albiglutide and insulin glargine in patients with type 2 diabetes inadequately controlled with metformin with or without sulfonylurea. *Diabetologia.* 2014;57(12):2475-2484.

**99.** Pratley RE, Nauck MA, Barnett AH, et al. Once-weekly albiglutide versus once-daily liraglutide in patients with type 2 diabetes inadequately controlled on oral drugs (HARMONY 7): a randomised, open-label, multicentre, non-inferiority phase 3 study. *Lancet Diabetes Endocrinol.* 2014;2(4):289-297.

**100.** Blonde L, Jendle J, Gross J, et al. Once-weekly dulaglutide versus bedtime insulin glargine, both in combination with prandial insulin lispro, in patients with type 2 diabetes (AWARD-4): a randomised, open-label, phase 3, non-inferiority study. *Lancet.* 2015;385(9982):2057-2066.

**101.** Ferdinand KC, White WB, Calhoun DA, et al. Effects of the once-weekly glucagon-like peptide-1 receptor agonist dulaglutide on ambulatory blood pressure and heart rate in patients with type 2 diabetes mellitus. *Hypertension.* 2014;64(4):731-737.

**102.** Umpierrez G, Tofe PS, Perez MF, Shurzinske L, Pechtner V. Efficacy and safety of dulaglutide monotherapy versus metformin in type 2 diabetes in a randomized controlled trial (AWARD-3). *Diabetes Care.* 2014;37(8):2168-2176.

**103.** Wysham C, Blevins T, Arakaki R, et al. Efficacy and safety of dulaglutide added onto pioglitazone and metformin versus exenatide in type 2 diabetes in a randomized controlled trial (AWARD-1). *Diabetes Care.* 2014;37(8):2159-2167.

**104.** Buse JB, Han J, Miller S, MacConell L, Pencek R, Wintle M. Addition of exenatide BID to insulin glargine: a post-hoc analysis of the effect on glycemia and weight across a range of insulin titration. *Curr Med Res Opin.* 2014;30(7):1209-1218.

**105.** Buse JB, Bergenstal RM, Glass LC, et al. Use of twice-daily exenatide in Basal insulin-treated patients with type 2 diabetes: a randomized, controlled trial. *Ann Intern Med.* 2011;154(2):103-112.

**106.** Simo R, Guerci B, Schernthaner G, et al. Long-term changes in cardiovascular risk markers during administration of exenatide twice daily or glimepiride: results from the European exenatide study. *Cardiovasc Diabetol.* 2015;14:116.

**107.** Gallwitz B, Guzman J, Dotta F, et al. Exenatide twice daily versus glimepiride for prevention of glycaemic deterioration in patients with type 2 diabetes with metformin failure (EUREXA): an open-label, randomised controlled trial. *Lancet.* 2012;379(9833):2270-2278.

**108.** Xu W, Bi Y, Sun Z, et al. Comparison of the effects on glycaemic control and beta-cell function in newly diagnosed type 2 diabetes patients of treatment with exenatide, insulin or pioglitazone: a multicentre randomized parallel-group trial (the CONFIDENCE study). *J Intern Med.* 2015;277(1):137-150.

**109.** Diamant M, Nauck MA, Shaginian R, et al. Glucagon-like peptide 1 receptor agonist or bolus insulin with optimized basal insulin in type 2 diabetes. *Diabetes Care.* 2014;37(10):2763-2773.

**110.** Diamant M, Van Gaal L, Guerci B, et al. Exenatide once weekly versus insulin glargine for type 2 diabetes (DURATION-3): 3-year results of an open-label randomised trial. *Lancet Diabetes Endocrinol.* 2014;2(6):464-473.

**111.** Diamant M, Van Gaal L, Stranks S, et al. Safety and efficacy of once-weekly exenatide compared with insulin glargine titrated to target in patients with type 2 diabetes over 84 weeks. *Diabetes Care.* 2012;35(4):683-689.

**112.** Diamant M, Van Gaal L, Stranks S, et al. Once weekly exenatide compared with insulin glargine titrated to target in patients with type 2 diabetes (DURATION-3): an open-label randomised trial. *Lancet.* 2010;375(9733):2234-2243.

**113.** Nauck MA, Duran S, Kim D, et al. A comparison of twice-daily exenatide and biphasic insulin aspart in patients with type 2 diabetes who were suboptimally controlled with sulfonylurea and metformin: a non-inferiority study. *Diabetologia.* 2007;50(2):259-267.

**114.** Inagaki N, Atsumi Y, Oura T, Saito H, Imaoka T. Efficacy and safety profile of exenatide once weekly compared with insulin once daily in Japanese patients with type 2 diabetes treated with oral antidiabetes drug(s): results from a 26-week, randomized, open-label, parallel-group, multicenter, noninferiority study. *Clin Ther.* 2012;34(9):1892-1908.

**115.** Gallwitz B, Bohmer M, Segiet T, et al. Exenatide twice daily versus premixed insulin aspart 70/30 in metformin-treated patients with type 2 diabetes: a randomized 26-week study on glycemic control and hypoglycemia. *Diabetes Care.* 2011;34(3):604-606.

**116.** Liutkus J, Rosas GJ, Norwood P, et al. A placebo-controlled trial of exenatide twice-daily added to thiazolidinediones alone or in combination with metformin. *Diabetes Obes Metab.* 2010;12(12):1058-1065.

**117.** Bergenstal R, Lewin A, Bailey T, Chang D, Gylvin T, Roberts V. Efficacy and safety of biphasic insulin aspart 70/30 versus exenatide in subjects with type 2 diabetes failing to achieve glycemic control with metformin and a sulfonylurea. *Curr Med Res Opin.* 2009;25(1):65-75.

**118.** Davies MJ, Donnelly R, Barnett AH, Jones S, Nicolay C, Kilcoyne A. Exenatide compared with long-acting insulin to achieve glycaemic control with minimal weight gain in patients with type 2 diabetes: results of the Helping Evaluate Exenatide in patients with diabetes compared with Long-Acting insulin (HEELA) study. *Diabetes Obes Metab.* 2009;11(12):1153-1162.

**119.** Buse JB, Henry RR, Han J, Kim DD, Fineman MS, Baron AD. Effects of exenatide (exendin-4) on glycemic control over 30 weeks in sulfonylurea-treated patients with type 2 diabetes. *Diabetes Care.* 2004;27(11):2628-2635.

**120.** Buse JB, Nauck M, Forst T, et al. Exenatide once weekly versus liraglutide once daily in patients with type 2 diabetes (DURATION-6): a randomised, open-label study. *Lancet.* 2013;381(9861):117-124.

**121.** Buse JB, Rosenstock J, Sesti G, et al. Liraglutide once a day versus exenatide twice a day for type 2 diabetes: a 26-week randomised, parallel-group, multinational, open-label trial (LEAD-6). *Lancet.* 2009;374(9683):39-47.

**122.** Marso SP, Daniels GH, Brown-Frandsen K, et al. Liraglutide and Cardiovascular Outcomes in Type 2 Diabetes. *N Engl J Med.* 2016.

**123.** D'Alessio D, Haring HU, Charbonnel B, et al. Comparison of insulin glargine and liraglutide added to oral agents in patients with poorly controlled type 2 diabetes. *Diabetes Obes Metab.* 2015;17(2):170-178.

**124.** Gough SC, Bode B, Woo V, et al. Efficacy and safety of a fixed-ratio combination of insulin degludec and liraglutide (IDegLira) compared with its components given alone: results of a phase 3, open-label, randomised, 26-week, treat-to-target trial in insulin-naive patients with type 2 diabetes. *Lancet Diabetes Endocrinol.* 2014;2(11):885-893.

**125.** Mathieu C, Rodbard HW, Cariou B, et al. A comparison of adding liraglutide versus a single daily dose of insulin aspart to insulin degludec in subjects with type 2 diabetes (BEGIN: VICTOZA ADD-ON). *Diabetes Obes Metab.* 2014;16(7):636-644.

**126.** Garber A, Henry RR, Ratner R, Hale P, Chang CT, Bode B. Liraglutide, a once-daily human glucagon-like peptide 1 analogue, provides sustained improvements in glycaemic control and weight for 2 years as monotherapy compared with glimepiride in patients with type 2 diabetes. *Diabetes Obes Metab.* 2011;13(4):348-356.

**127.** Bode BW, Testa MA, Magwire M, et al. Patient-reported outcomes following treatment with the human GLP-1 analogue liraglutide or glimepiride in monotherapy: results from a randomized controlled trial in patients with type 2 diabetes. *Diabetes Obes Metab.* 2010;12(7):604-612.

**128.** Garber A, Henry R, Ratner R, et al. Liraglutide versus glimepiride monotherapy for type 2 diabetes (LEAD-3 Mono): a randomised, 52-week, phase III, double-blind, parallel-treatment trial. *Lancet.* 2009;373(9662):473-481.

**129.** Nauck M, Frid A, Hermansen K, et al. Efficacy and safety comparison of liraglutide, glimepiride, and placebo, all in combination with metformin, in type 2 diabetes: the LEAD (liraglutide effect and action in diabetes)-2 study. *Diabetes Care.* 2009;32(1):84-90.

**130.** Davies MJ, Bergenstal R, Bode B, et al. Efficacy of Liraglutide for Weight Loss Among Patients With Type 2 Diabetes: The SCALE Diabetes Randomized Clinical Trial. *JAMA.* 2015;314(7):687-699.

**131.** Pfeffer MA, Claggett B, Diaz R, et al. Lixisenatide in Patients with Type 2 Diabetes and Acute Coronary Syndrome. *N Engl J Med.* 2015;373(23):2247-2257.

**132.** Rosenstock J, Hanefeld M, Shamanna P, et al. Beneficial effects of once-daily lixisenatide on overall and postprandial glycemic levels without significant excess of hypoglycemia in type 2 diabetes inadequately controlled on a sulfonylurea with or without metformin (GetGoal-S). *J Diabetes Complications.* 2014;28(3):386-392.

**133.** Yu PC, Han P, Liu X, et al. Lixisenatide treatment improves glycaemic control in Asian patients with type 2 diabetes mellitus inadequately controlled on metformin with or without sulfonylurea: a randomized, double-blind, placebo-controlled, 24-week trial (GetGoal-M-Asia). *Diabetes Metab Res Rev.* 2014;30(8):726-735.

**134.** Pinget M, Goldenberg R, Niemoeller E, Muehlen-Bartmer I, Guo H, Aronson R. Efficacy and safety of lixisenatide once daily versus placebo in type 2 diabetes insufficiently controlled on pioglitazone (GetGoal-P). *Diabetes Obes Metab.* 2013;15(11):1000-1007.

**135.** Riddle MC, Aronson R, Home P, et al. Adding once-daily lixisenatide for type 2 diabetes inadequately controlled by established basal insulin: a 24-week, randomized, placebo-controlled comparison (GetGoal-L). *Diabetes Care.* 2013;36(9):2489-2496.

**136.** Riddle MC, Forst T, Aronson R, et al. Adding once-daily lixisenatide for type 2 diabetes inadequately controlled with newly initiated and continuously titrated basal insulin glargine: a 24-week, randomized, placebo-controlled study (GetGoal-Duo 1). *Diabetes Care.* 2013;36(9):2497-2503.

**137.** Seino Y, Min KW, Niemoeller E, Takami A. Randomized, double-blind, placebo-controlled trial of the once-daily GLP-1 receptor agonist lixisenatide in Asian patients with type 2 diabetes insufficiently controlled on basal insulin with or without a sulfonylurea (GetGoal-L-Asia). *Diabetes Obes Metab.* 2012;14(10):910-917.

**138.** Bolli GB, Munteanu M, Dotsenko S, et al. Efficacy and safety of lixisenatide once daily vs. placebo in people with Type 2 diabetes insufficiently controlled on metformin (GetGoal-F1). *Diabet Med.* 2014;31(2):176-184.

**139.** Rosenstock J, Raccah D, Koranyi L, et al. Efficacy and safety of lixisenatide once daily versus exenatide twice daily in type 2 diabetes inadequately controlled on metformin: a 24-week, randomized, open-label, active-controlled study (GetGoal-X). *Diabetes Care.* 2013;36(10):2945-2951.

**140.** Bode B, Stenlof K, Harris S, et al. Long-term efficacy and safety of canagliflozin over 104 weeks in patients aged 55-80 years with type 2 diabetes. *Diabetes Obes Metab.* 2015;17(3):294-303.

**141.** Bode B, Stenlof K, Sullivan D, Fung A, Usiskin K. Efficacy and safety of canagliflozin treatment in older subjects with type 2 diabetes mellitus: a randomized trial. *Hosp Pract (1995).* 2013;41(2):72-84.

**142.** Leiter LA, Yoon KH, Arias P, et al. Canagliflozin provides durable glycemic improvements and body weight reduction over 104 weeks versus glimepiride in patients with type 2 diabetes on metformin: a randomized, double-blind, phase 3 study. *Diabetes Care.* 2015;38(3):355-364.

**143.** Cefalu WT, Leiter LA, Yoon KH, et al. Efficacy and safety of canagliflozin versus glimepiride in patients with type 2 diabetes inadequately controlled with metformin (CANTATA-SU): 52 week results from a randomised, double-blind, phase 3 non-inferiority trial. *Lancet.* 2013;382(9896):941-950.

**144.** Stenlof K, Cefalu WT, Kim KA, et al. Long-term efficacy and safety of canagliflozin monotherapy in patients with type 2 diabetes inadequately controlled with diet and exercise: findings from the 52-week CANTATA-M study. *Curr Med Res Opin.* 2014;30(2):163-175.

**145.** Stenlof K, Cefalu WT, Kim KA, et al. Efficacy and safety of canagliflozin monotherapy in subjects with type 2 diabetes mellitus inadequately controlled with diet and exercise. *Diabetes Obes Metab.* 2013;15(4):372-382.

**146.** Yale JF, Bakris G, Cariou B, et al. Efficacy and safety of canagliflozin over 52 weeks in patients with type 2 diabetes mellitus and chronic kidney disease. *Diabetes Obes Metab.* 2014;16(10):1016-1027.

**147.** Yale JF, Bakris G, Cariou B, et al. Efficacy and safety of canagliflozin in subjects with type 2 diabetes and chronic kidney disease. *Diabetes Obes Metab.* 2013;15(5):463-473.

**148.** Wilding JP, Charpentier G, Hollander P, et al. Efficacy and safety of canagliflozin in patients with type 2 diabetes mellitus inadequately controlled with metformin and sulphonylurea: a randomised trial. *Int J Clin Pract.* 2013;67(12):1267-1282.

**149.** Forst T, Guthrie R, Goldenberg R, et al. Efficacy and safety of canagliflozin over 52 weeks in patients with type 2 diabetes on background metformin and pioglitazone. *Diabetes Obes Metab.* 2014;16(5):467-477.

**150.** Neal B, Perkovic V, de Zeeuw D, et al. Efficacy and safety of canagliflozin, an inhibitor of sodium-glucose cotransporter 2, when used in conjunction with insulin therapy in patients with type 2 diabetes. *Diabetes Care.* 2015;38(3):403-411.

**151.** Bailey CJ, Morales VE, Woo V, Tang W, Ptaszynska A, List JF. Efficacy and safety of dapagliflozin monotherapy in people with Type 2 diabetes: a randomized double-blind placebo-controlled 102-week trial. *Diabet Med.* 2015;32(4):531-541.

**152.** Ferrannini E, Ramos SJ, Salsali A, Tang W, List JF. Dapagliflozin monotherapy in type 2 diabetic patients with inadequate glycemic control by diet and exercise: a randomized, double-blind, placebo-controlled, phase 3 trial. *Diabetes Care.* 2010;33(10):2217-2224.

**153.** Cefalu WT, Leiter LA, de Bruin TW, Gause-Nilsson I, Sugg J, Parikh SJ. Dapagliflozin's Effects on Glycemia and Cardiovascular Risk Factors in High-Risk Patients With Type 2 Diabetes: A 24-Week, Multicenter, Randomized, Double-Blind, Placebo-Controlled Study With a 28-Week Extension. *Diabetes Care.* 2015;38(7):1218-1227.

**154.** Del PS, Nauck M, Duran-Garcia S, et al. Long-term glycaemic response and tolerability of dapagliflozin versus a sulphonylurea as add-on therapy to metformin in patients with type 2 diabetes: 4-year data. *Diabetes Obes Metab.* 2015;17(6):581-590.

**155.** Nauck MA, Del PS, Duran-Garcia S, et al. Durability of glycaemic efficacy over 2 years with dapagliflozin versus glipizide as add-on therapies in patients whose type 2 diabetes mellitus is inadequately controlled with metformin. *Diabetes Obes Metab.* 2014;16(11):1111-1120.

**156.** Nauck MA, Del PS, Meier JJ, et al. Dapagliflozin versus glipizide as add-on therapy in patients with type 2 diabetes who have inadequate glycemic control with metformin: a randomized, 52-week, double-blind, active-controlled noninferiority trial. *Diabetes Care.* 2011;34(9):2015-2022.

**157.** Jabbour SA, Hardy E, Sugg J, Parikh S. Dapagliflozin is effective as add-on therapy to sitagliptin with or without metformin: a 24-week, multicenter, randomized, double-blind, placebo-controlled study. *Diabetes Care.* 2014;37(3):740-750.

**158.** Kohan DE, Fioretto P, Tang W, List JF. Long-term study of patients with type 2 diabetes and moderate renal impairment shows that dapagliflozin reduces weight and blood pressure but does not improve glycemic control. *Kidney Int.* 2014;85(4):962-971.

**159.** Leiter LA, Cefalu WT, de Bruin TW, Gause-Nilsson I, Sugg J, Parikh SJ. Dapagliflozin added to usual care in individuals with type 2 diabetes mellitus with preexisting cardiovascular disease: a 24-week, multicenter, randomized, double-blind, placebo-controlled study with a 28-week extension. *J Am Geriatr Soc.* 2014;62(7):1252-1262.

**160.** Wilding JP, Woo V, Rohwedder K, Sugg J, Parikh S. Dapagliflozin in patients with type 2 diabetes receiving high doses of insulin: efficacy and safety over 2 years. *Diabetes Obes Metab.* 2014;16(2):124-136.

**161.** Wilding JP, Woo V, Soler NG, et al. Long-term efficacy of dapagliflozin in patients with type 2 diabetes mellitus receiving high doses of insulin: a randomized trial. *Ann Intern Med.* 2012;156(6):405-415.

**162.** Bailey CJ, Gross JL, Hennicken D, Iqbal N, Mansfield TA, List JF. Dapagliflozin add-on to metformin in type 2 diabetes inadequately controlled with metformin: a randomized, double-blind, placebo-controlled 102-week trial. *BMC Med.* 2013;11:43.

**163.** Bailey CJ, Iqbal N, T'Joen C, List JF. Dapagliflozin monotherapy in drug-naive patients with diabetes: a randomized-controlled trial of low-dose range. *Diabetes Obes Metab.* 2012;14(10):951-959.

**164.** Henry RR, Murray AV, Marmolejo MH, Hennicken D, Ptaszynska A, List JF. Dapagliflozin, metformin XR, or both: initial pharmacotherapy for type 2 diabetes, a randomised controlled trial. *Int J Clin Pract.* 2012;66(5):446-456.

**165.** Ljunggren O, Bolinder J, Johansson L, et al. Dapagliflozin has no effect on markers of bone formation and resorption or bone mineral density in patients with inadequately controlled type 2 diabetes mellitus on metformin. *Diabetes Obes Metab.* 2012;14(11):990-999.

**166.** Rosenstock J, Vico M, Wei L, Salsali A, List JF. Effects of dapagliflozin, an SGLT2 inhibitor, on HbA(1c), body weight, and hypoglycemia risk in patients with type 2 diabetes inadequately controlled on pioglitazone monotherapy. *Diabetes Care.* 2012;35(7):1473-1478.

**167.** Strojek K, Yoon KH, Hruba V, Elze M, Langkilde AM, Parikh S. Effect of dapagliflozin in patients with type 2 diabetes who have inadequate glycaemic control with glimepiride: a randomized, 24-week, double-blind, placebo-controlled trial. *Diabetes Obes Metab.* 2011;13(10):928-938.

**168.** Zinman B, Wanner C, Lachin JM, et al. Empagliflozin, Cardiovascular Outcomes, and Mortality in Type 2 Diabetes. *N Engl J Med.* 2015;373(22):2117-2128.

**169.** Kovacs CS, Seshiah V, Merker L, et al. Empagliflozin as Add-on Therapy to Pioglitazone With or Without Metformin in Patients With Type 2 Diabetes Mellitus. *Clin Ther.* 2015;37(8):1773-1788.

**170.** Araki E, Tanizawa Y, Tanaka Y, et al. Long-term treatment with empagliflozin as add-on to oral antidiabetes therapy in Japanese patients with type 2 diabetes mellitus. *Diabetes Obes Metab.* 2015;17(7):665-674.

**171.** Barnett AH, Mithal A, Manassie J, et al. Efficacy and safety of empagliflozin added to existing antidiabetes treatment in patients with type 2 diabetes and chronic kidney disease: a randomised, double-blind, placebo-controlled trial. *Lancet Diabetes Endocrinol.* 2014;2(5):369-384.

**172.** Ridderstrale M, Andersen KR, Zeller C, Kim G, Woerle HJ, Broedl UC. Comparison of empagliflozin and glimepiride as add-on to metformin in patients with type 2 diabetes: a 104-week randomised, active-controlled, double-blind, phase 3 trial. *Lancet Diabetes Endocrinol.* 2014;2(9):691-700.

**173.** Rosenstock J, Jelaska A, Frappin G, et al. Improved glucose control with weight loss, lower insulin doses, and no increased hypoglycemia with empagliflozin added to titrated multiple daily injections of insulin in obese inadequately controlled type 2 diabetes. *Diabetes Care.* 2014;37(7):1815-1823.

**174.** Giles TD, Elkayam U, Bhattacharya M, Perez A, Miller AB. Comparison of pioglitazone vs glyburide in early heart failure: insights from a randomized controlled study of patients with type 2 diabetes and mild cardiac disease. *Congest Heart Fail.* 2010;16(3):111-117.

**175.** Home PD, Pocock SJ, Beck-Nielsen H, et al. Rosiglitazone evaluated for cardiovascular outcomes--an interim analysis. *N Engl J Med.* 2007;357(1):28-38.

**176.** Mazzone T, Meyer PM, Feinstein SB, et al. Effect of pioglitazone compared with glimepiride on carotid intima-media thickness in type 2 diabetes: a randomized trial. *JAMA.* 2006;296(21):2572-2581.

**177.** Intensive blood-glucose control with sulphonylureas or insulin compared with conventional treatment and risk of complications in patients with type 2 diabetes (UKPDS 33). UK Prospective Diabetes Study (UKPDS) Group. *Lancet.* 1998;352(9131):837-853.

**178.** Hamann A, Garcia-Puig J, Paul G, Donaldson J, Stewart M. Comparison of fixed-dose rosiglitazone/metformin combination therapy with sulphonylurea plus metformin in overweight individuals with Type 2 diabetes inadequately controlled on metformin alone. *Exp Clin Endocrinol Diabetes.* 2008;116(1):6-13.

**179.** Hong J, Zhang Y, Lai S, et al. Effects of metformin versus glipizide on cardiovascular outcomes in patients with type 2 diabetes and coronary artery disease. *Diabetes Care.* 2013;36(5):1304-1311.

**180.** Chou HS, Truitt KE, Moberly JB, et al. A 26-week, placebo- and pioglitazone-controlled monotherapy study of rivoglitazone in subjects with type 2 diabetes mellitus. *Diabetes Obes Metab.* 2012;14(11):1000-1009.

**181.** Gerstein HC, Ratner RE, Cannon CP, et al. Effect of rosiglitazone on progression of coronary atherosclerosis in patients with type 2 diabetes mellitus and coronary artery disease: the assessment on the prevention of progression by rosiglitazone on atherosclerosis in diabetes patients with cardiovascular history trial. *Circulation.* 2010;121(10):1176-1187.

**182.** Tolman KG, Freston JW, Kupfer S, Perez A. Liver safety in patients with type 2 diabetes treated with pioglitazone: results from a 3-year, randomized, comparator-controlled study in the US. *Drug Saf.* 2009;32(9):787-800.

**183.** Nissen SE, Nicholls SJ, Wolski K, et al. Comparison of pioglitazone vs glimepiride on progression of coronary atherosclerosis in patients with type 2 diabetes: the PERISCOPE randomized controlled trial. *JAMA.* 2008;299(13):1561-1573.

**184.** Bakris GL, Ruilope LM, McMorn SO, et al. Rosiglitazone reduces microalbuminuria and blood pressure independently of glycemia in type 2 diabetes patients with microalbuminuria. *J Hypertens.* 2006;24(10):2047-2055.

**185.** Jain R, Osei K, Kupfer S, Perez AT, Zhang J. Long-term safety of pioglitazone versus glyburide in patients with recently diagnosed type 2 diabetes mellitus. *Pharmacotherapy.* 2006;26(10):1388-1395.

**186.** Kahn SE, Haffner SM, Heise MA, et al. Glycemic durability of rosiglitazone, metformin, or glyburide monotherapy. *N Engl J Med.* 2006;355(23):2427-2443.

**187.** Rosenstock J, Rood J, Cobitz A, Biswas N, Chou H, Garber A. Initial treatment with rosiglitazone/metformin fixed-dose combination therapy compared with monotherapy with either rosiglitazone or metformin in patients with uncontrolled type 2 diabetes. *Diabetes Obes Metab.* 2006;8(6):650-660.

**188.** Charbonnel B, Schernthaner G, Brunetti P, et al. Long-term efficacy and tolerability of add-on pioglitazone therapy to failing monotherapy compared with addition of gliclazide or metformin in patients with type 2 diabetes. *Diabetologia.* 2005;48(6):1093-1104.

**189.** Dailey GR, Noor MA, Park JS, Bruce S, Fiedorek FT. Glycemic control with glyburide/metformin tablets in combination with rosiglitazone in patients with type 2 diabetes: a randomized, double-blind trial. *Am J Med.* 2004;116(4):223-229.

**190.** Schernthaner G, Matthews DR, Charbonnel B, Hanefeld M, Brunetti P. Efficacy and safety of pioglitazone versus metformin in patients with type 2 diabetes mellitus: a double-blind, randomized trial. *J Clin Endocrinol Metab.* 2004;89(12):6068-6076.

**191.** Fonseca V, Rosenstock J, Patwardhan R, Salzman A. Effect of metformin and rosiglitazone combination therapy in patients with type 2 diabetes mellitus: a randomized controlled trial. *JAMA.* 2000;283(13):1695-1702.

**192.** Horton ES, Clinkingbeard C, Gatlin M, Foley J, Mallows S, Shen S. Nateglinide alone and in combination with metformin improves glycemic control by reducing mealtime glucose levels in type 2 diabetes. *Diabetes Care.* 2000;23(11):1660-1665.

**193.** Johnston PS, Lebovitz HE, Coniff RF, Simonson DC, Raskin P, Munera CL. Advantages of alpha-glucosidase inhibition as monotherapy in elderly type 2 diabetic patients. *J Clin Endocrinol Metab.* 1998;83(5):1515-1522.

**194.** DeFronzo RA, Goodman AM. Efficacy of metformin in patients with non-insulin-dependent diabetes mellitus. The Multicenter Metformin Study Group. *N Engl J Med.* 1995;333(9):541-549.
